# *TECPR2* Associated Neuroaxonal Dystrophy in Spanish Water Dogs

**DOI:** 10.1371/journal.pone.0141824

**Published:** 2015-11-10

**Authors:** Kerstin Hahn, Cecilia Rohdin, Vidhya Jagannathan, Peter Wohlsein, Wolfgang Baumgärtner, Frauke Seehusen, Ingo Spitzbarth, Rodrigo Grandon, Cord Drögemüller, Karin Hultin Jäderlund

**Affiliations:** 1 University of Veterinary Medicine Hannover, Department of Pathology, Hannover, Germany; 2 University Animal Hospital, Swedish University of Agricultural Sciences, Uppsala, Sweden; 3 Institute of Genetics, Vetsuisse Faculty, University of Bern, Bern, Switzerland; 4 Department of Biomedical Sciences and Veterinary Public Health, Division of Pathology, Pharmacology and Toxicology, Swedish University of Agricultural Sciences, Uppsala, Sweden; 5 Department of Companion Animal Clinical Sciences, Norwegian University of Life Sciences, Oslo, Norway; 6 Center for Systems Neuroscience, Hannover, Germany; 7 Anicura, Albano Small Animal Hospital, Danderyd, Sweden; Cambridge University, UNITED KINGDOM

## Abstract

Clinical, pathological and genetic examination revealed an as yet uncharacterized juvenile-onset neuroaxonal dystrophy (NAD) in Spanish water dogs. Affected dogs presented with various neurological deficits including gait abnormalities and behavioral deficits. Histopathology demonstrated spheroid formation accentuated in the grey matter of the cerebral hemispheres, the cerebellum, the brain stem and in the sensory pathways of the spinal cord. Iron accumulation was absent. Ultrastructurally spheroids contained predominantly closely packed vesicles with a double-layered membrane, which were characterized as autophagosomes using immunohistochemistry. The family history of the four affected dogs suggested an autosomal recessive inheritance. SNP genotyping showed a single genomic region of extended homozygosity of 4.5 Mb in the four cases on CFA 8. Linkage analysis revealed a maximal parametric LOD score of 2.5 at this region. By whole genome re-sequencing of one affected dog, a perfectly associated, single, non-synonymous coding variant in the canine tectonin beta-propeller repeat-containing protein 2 (*TECPR2)* gene affecting a highly conserved region was detected (c.4009C>T or p.R1337W). This canine NAD form displays etiologic parallels to an inherited *TECPR2* associated type of human hereditary spastic paraparesis (HSP). In contrast to the canine NAD, the spinal cord lesions in most types of human HSP involve the sensory and the motor pathways. Furthermore, the canine NAD form reveals similarities to cases of human NAD defined by widespread spheroid formation without iron accumulation in the basal ganglia. Thus *TECPR2* should also be considered as candidate gene for human NAD. Immunohistochemistry and the ultrastructural findings further support the assumption, that *TECPR2* regulates autophagosome accumulation in the autophagic pathways. Consequently, this report provides the first genetic characterization of juvenile canine NAD, describes the histopathological features associated with the *TECPR2* mutation and provides evidence to emphasize the association between failure of autophagy and neurodegeneration.

## Introduction

Neuroaxonal dystrophies (NAD) in humans and animals represent a group of rare, heterogeneous inherited neurodegenerative conditions with clinical and pathological overlapping manifestations [1], [2]. Although they all share the characteristic pathologic feature, i.e. the development of spheroids, there is variation in the clinical and the neurological signs, the progression of the disease and lesion distribution between, but also within species. Besides presenting as a primary central nervous system (CNS) disorder, NAD-like findings may occur associated with aging and secondary to several metabolic-toxic conditions [3]. The nomenclature of primary human NADs is complex due to the classification of the subtypes according to i) historical terminations, ii) underlying genetic mutations, iii) the presence or absence of iron accumulations in the basal ganglia, or iv) the age of onset and clinical symptoms (**S1 Table**). The prevalent genetic associations for human NAD comprise the autosomal recessive inherited mutations in pantothenate kinase 2 (*PANK2*; OMIM 606157), phospholipase A2, group VI (*PLA2G6*; OMIM 603604), and chromosome 19 open reading frame 12 (*C19orf12*; OMIM 614297), whereas other types of NAD are less frequent [4], [5], [6], [7]. Recently, one X-linked variant associated with a mutation in the autophagy related WD repeat domain 45 (*WDR45*; OMIM 300526) gene was reported, representing the first direct link between the autophagy machinery and neurodegeneration [8]. However, numerous idiopathic types of late infantile, juvenile, and adult NAD are genetically not classified [9]. The histological hallmark of NAD is defined by localized axonal swellings (spheroids) with distal axonal atrophy and secondary myelin degradation [9], [10]. Spheroids containing protein aggregations, membranous vesicular structures, mitochondria, and/or neurofilaments are also found in amyotrophic lateral sclerosis, Huntington’s disease, Alzheimer’s and familial Parkinson’s disease as well as human hereditary spastic paraparesis (HSP) [11], [12], [13], [14], [15], [16]. In all these neurodegenerative conditions, impairment of autophagy is discussed as a crucial pathomechanism [17].

Autophagy, which is part of the normal cell homeostasis, is involved in the basal constitutive turnover of cytosolic components and is activated by stress signals such as nutrient starvation and oxidative stress. The first step in this process implies the sequestration of damaged organelles, long-lived proteins, and protein aggregations into double-membrane vesicles called autophagosomes. Fusion of autophagosome and lysosome provides further degradation and subsequent release of amino acids and other molecules into the cytoplasm [18]. Autophagy is especially important for the metabolic homeostasis of neurons as post-mitotic cells with a high energy demand [17]. Due to genetic associations and corresponding experimental studies, the relevance of this pathway and its implication as a therapeutic target in the treatment of neurodegenerative diseases is a current topic in neuroscience.

Canine NAD has previously been reported in Collie sheep dogs, Rottweiler dogs, Jack Russel Terriers and Papillon dogs. However, only one form of canine NAD with fetal onset in laboratory dogs (Schnauzer-Beagle crossbreed) has been characterized at the molecular level [2], [19]. The affected gene encodes mitofusin 2 (MFN2; OMIA 000715–9615), a protein mediating mitochondrial fusion but also clearance of damaged mitochondria via selective autophagy [19]. The present study reports positional cloning of a *TECPR2* missense mutation causing NAD in Spanish water dogs, with evidence of disturbances of the neuronal autophagy pathway.

## Results

### Clinical and histopathological characterization of NAD in the Spanish water dog

Four Spanish water dogs presented with slowly progressing neurological signs starting between six and eleven months of age. Owners reported gait abnormalities, behavioral changes (dullness, nervousness, vocalization) and incontinence alone or in combination with uncontrolled defecation. All dogs showed a mild head tilt, generalized mild cerebellar ataxia with hypermetria of the thoracic limbs and absent to depressed patellar reflexed. Additionally, affected dogs displayed varying degrees of compulsory pacing, proprioceptive deficits, decreased menace, visual deficits, positional nystagmus and decreased muscle tone (**S1 Video).** Based on the neurological examination the disorder is characterized by a multifocal, predominantly sensory, localization. Complete blood count and chemistry profile were within reference ranges. Analysis of the cerebrospinal fluid showed normal cell counts and protein values. All affected dogs were euthanized at the owners request between 12 and 23 months of age.

**Fig 1:**
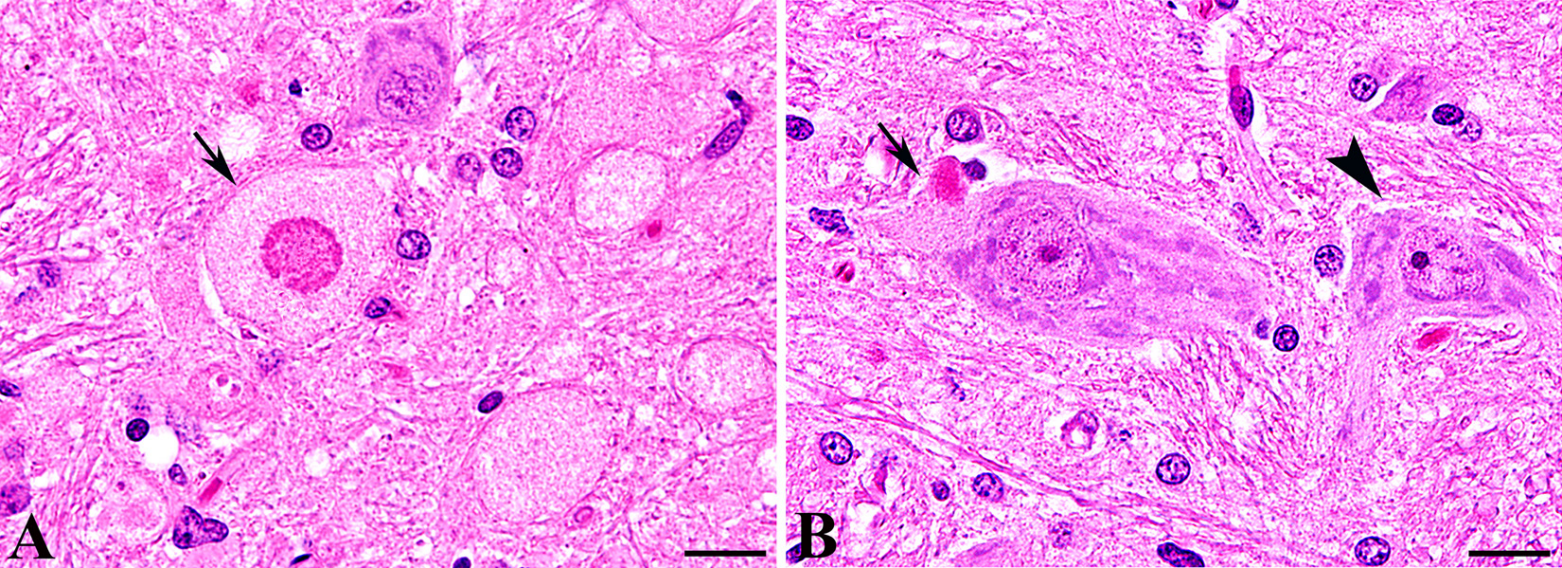
Histology of NAD in Spanish water dogs. (**A**, **B**) Histology of the brain stem (cuneate nucleus) stained with hematoxylin and eosin revealed numerous large granular axonal swellings (spheroids; arrow). Note the hypereosinophilic central target-like core structure of distinct spheroids (**A**). Single neurons displayed an accumulation of a finely or coarse granular, intensely eosinophilic material associated with the soma (arrow) displacing the Nissl substance. A high proportion of neurons adjacent to affected areas displayed a normal morphology with equally distributed Nissl substance (arrowhead, **B**).

In all affected dogs pathomorphological findings were restricted to the CNS. In the brain, neuronal loss and spheroid formation of variable degree was present, accentuated within the grey matter including the cerebral hemispheres, the cerebellum, and the brain stem. The lesions were most prominent and consistently found in the dorsolateral nuclei of the brain stem, including the cuneate and vestibular nuclei and the nucleus of the spinal tract of the trigeminal nerve. Sporadic spheroids were also present in the white matter of the brain. In the spinal cord neuronal loss and/or spheroid formation was restricted to the sensory pathways including the dorsal horn as well as the gracile and the cuneate fasciculi. In the spinal cord of diseased animals the number of spheroids ranged between 38 and 103 spheroids per mm^2^ in the gray matter and between 1 to 1.5 spheroids per mm^2^ in the white matter. No spheroids were found in the spinal cord of four age matched control beagle dogs. The spheroids in diseased Spanish water dogs represented demarcated nodular swellings with accumulations of a finely granular eosinophilic material. Several spheroids displayed a central hypereosinophilic target-like core structure (**Fig 1A**). Similar structural alterations were additionally detected in few neurons associated with the soma (**Fig 1B**). Using Turnbull’s blue and Prussian blue staining, no iron deposition was observed within the CNS (**S1 Fig**). No spheroid formation was diagnosed in the peripheral nerves.

### NAD in Spanish water dogs maps to a 4.5 Mb region on CFA 8

**Fig 2:**
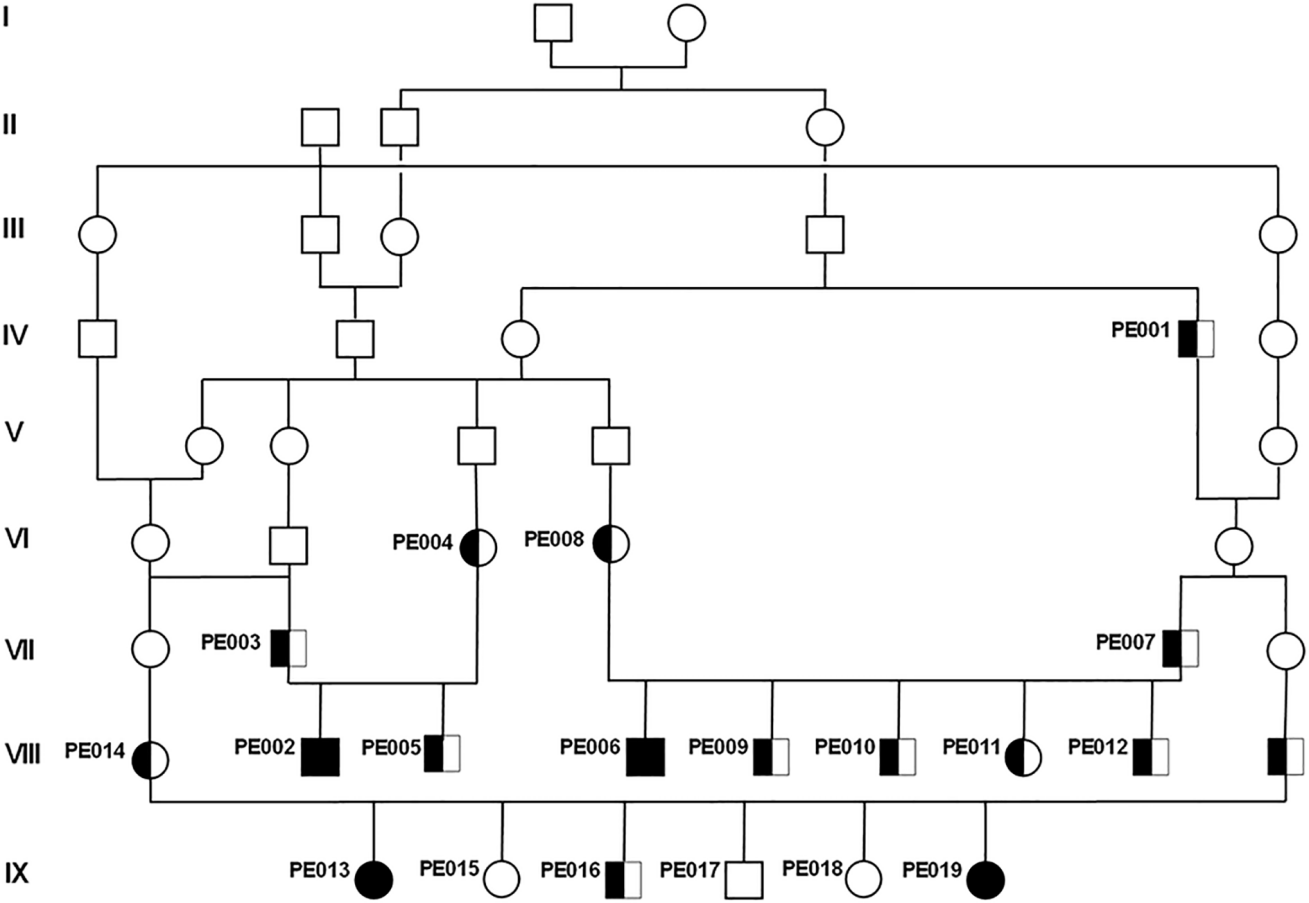
Pedigree of the collected Spanish water dogs with NAD. Note the inbreeding loops and the likely common ancestors appearing 8 to 9 generations ago. Only for the numbered animals DNA was available. Affected animals are shown with black symbols; genotyped carriers of the causative mutation are indicated with half-filled symbols; females are shown as circles and males as squares.

To characterize a possible gene defect, blood samples from the four diseased dogs and 15 related dogs were collected (**Fig 2**). Both, female and male offspring were affected. Parents of affected dogs showed no clinical signs of the disease. The four affected dogs could be traced back to common ancestors (**Fig 2**). The pedigree therefore indicated a monogenic autosomal recessive inheritance of NAD. Under this scenario the NAD affected dogs were considered to be identical by descent (IBD) for the causative mutation and flanking chromosomal segments. A homozygosity mapping approach was therefore applied to determine the position of the mutation in the canine genome. More than 170 000 evenly spaced SNPs of the four affected and 13 phenotypically healthy Spanish water dogs were genotyped. The four cases were analyzed for extended regions of homozygosity with simultaneous allele sharing. A total of four genomic regions were identified being IBD in the genotyped cases. Only on CFA 8, in a region containing 288 SNP markers corresponding to a 4.47 Mb interval from 67.54 to 72.01 Mb all diseased dogs were homozygous in contrast to the 13 phenotypical healthy controls. The other three regions of shared homozygosity among the four cases were located on CFA 13, 25, and 32 and contained only 69, 55, and 3 SNPs corresponding to 0.88, 0.71, and 0.03 kb, respectively. For linkage analysis, the pedigree was split into two sub-pedigrees because of inbreeding loops and missing DNA samples. The estimated maximal parametric LOD score of 2.5 at 70 Mb on CFA 8 suggested linkage of NAD to the candidate region identified before (**S2 Fig**).

### A *TECPR2* missense mutation is associated with NAD in Spanish water dogs

A total of 58 genes and loci are annotated in the critical interval on CFA 8. To obtain a comprehensive overview of all variants in the critical interval the whole genome of one NAD affected Spanish water dog was sequenced. The recessive inheritance and the fatal effect of the mutation suggested a loss of function mutation affecting splicing, expression or the coding sequence of the gene responsible for this type of NAD. Therefore, special emphasis was paid to variants located within the exons or within the splice sites of the annotated genes in the targeted region of the canine genome. SNP and indel variants were characterized with respect to the reference genome of a presumably non-affected Boxer (CanFam 3.1). Additionally, the genotypes of the affected dog were aligned with 119 dog genomes of various breeds that had been sequenced in the course of other ongoing studies. The pedigree analysis and the large size of the IBD segment indicated a relatively young origin of the mutation. Thus it was hypothesized that the mutant allele of the causative variant should be completely absent in all other dog breeds except the Spanish water dogs. It was considered unlikely that the mutant allele would have been introgressed into any other breeds outside the Spanish water dog. Within the critical interval on CFA 8, 63 private variants were noticed, of which only a single one was predicted to affect the coding sequence of an annotated gene. Only the affected dog had the homozygous variant genotype and all other 119 sequenced dogs carried the homozygous wildtype genotype. This remaining private non-synonymous variant in the tectonin beta-propeller repeat-containing protein 2 gene (*TECPR2* c.4009C>T) was genotyped in larger cohorts of dogs including the family members, unrelated controls of Spanish water dogs, and dogs of related breeds like other Iberian dog breeds (**Table 1**). The *TECPR2* variant remained perfectly associated with the NAD phenotype in more than 300 Spanish water dogs. Within the family material, only affected dogs were homozygous *TT* and available parents and grandparents were heterozygous *CT*. The variant was absent from a selection of dogs from other breeds.

**Table 1:** Association of the *TECPR2* missense mutation with the NAD phenotype in Spanish water dogs.

	*TECPR2* (c.4009C>T)		
	*CC*	*CT*	*TT*
**Spanish water dog (Perro de Agua Español)**			
Cases			4
Relatives	3	12	
Controls	261	21	
**Related dog breeds**			
Barbet	20		
Briard	5		
Cão da Serra de Aires	15		
Portugese Waterdog (Cão de Água Português)	3		
Lagotto Romagnolo	67		
Standard Poodle	13		
**Other breeds**	146		
**Total**	533	33	4

### The TECPR2 mutation affects a highly conserved domain

**Fig 3:**
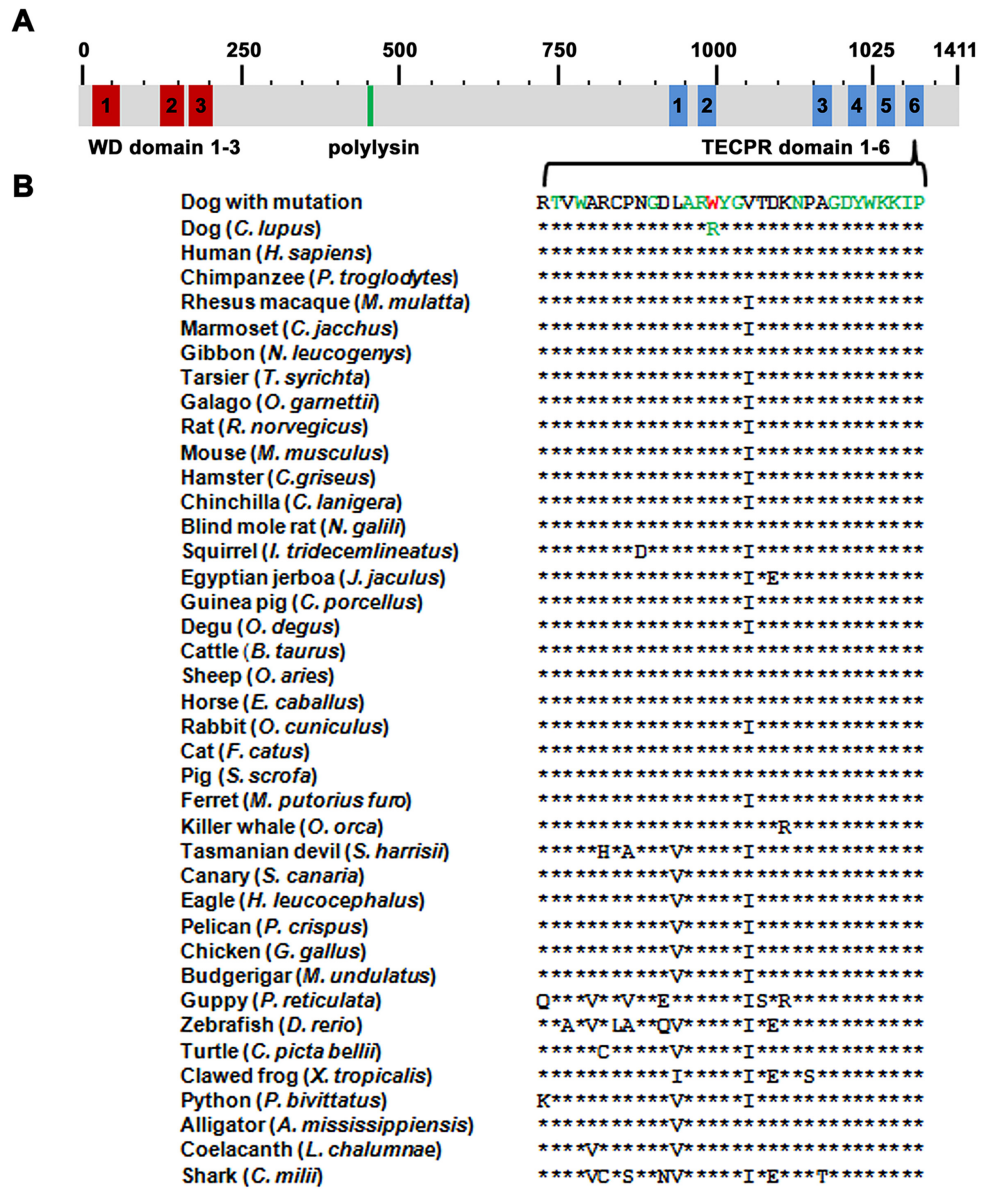
TECPR2 domain structure and p.R1337W mutation associated with NAD in Spanish water dogs. (**A**) TECPR2 possesses three N-terminal WD (tryptophan-aspartic-acid dipeptide) repeats (red), a polylysine tract (green), and six C-terminal tectonin beta-propeller repeat (TECPR) domains (blue). (**B**) The arginine at position 1337 that is substituted by a tryptophan residue (red) is located in the sixth TECPR domain. Note that the mutation affects a conserved amino acid residue in all known TECPR2 orthologs. Highly conserved residues are marked in green.

The NAD associated mutation in Spanish water dogs is located within the tectonin beta propeller repeat domaine 6 of the TECPR2 protein (**Fig 3A and 3B**) resulting on protein level in an exchange of the basic amino acid arginine against the nonpolar, aromatic tryptophan (p.R1337W). Multiple species protein alignment showed that the wildtype residue at the affected position is conserved across all known TECPR2 orthologs in vertebrates including the zebrafish (*Danio rerio*; **Fig 3B**). Alignment with the related TECPR1 paralogs revealed that the affected protein motif is highly conserved including in nematodes and the fruit fly paralog (**S3 Fig**). Software based analysis of the NAD associated TECPR2 amino acid exchange characterized the mutation as destabilizing (PoPMuSiC) and highly damaging (PolyPhen 2), whereas no effect on the secondary structure and disorder were predicted (NetTurnP, NetSurfP, CFSSP, Phyre 2).

### The TECPR2 mutation results in autophagosome accumulation within axons

**Fig 4:**
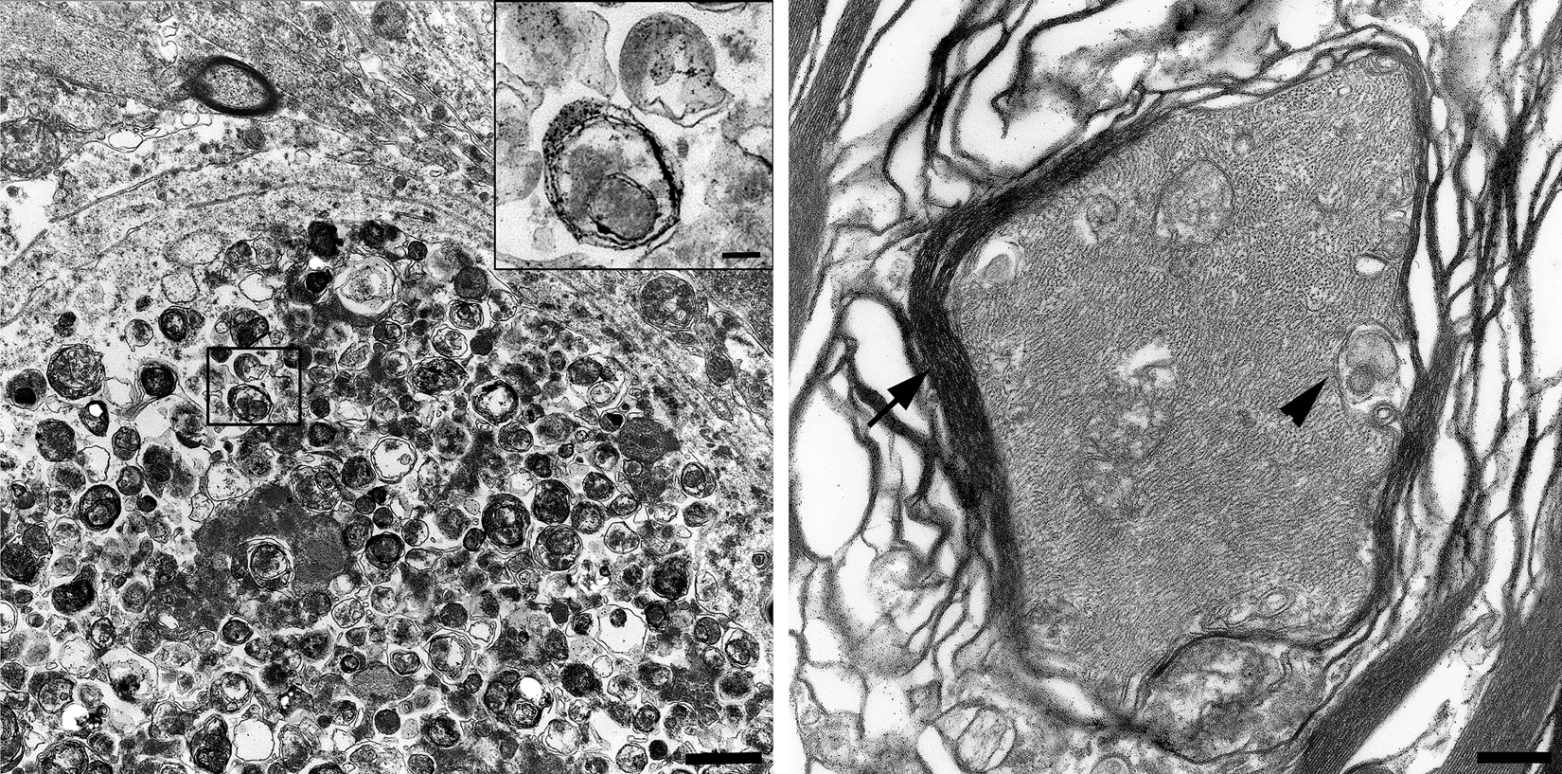
Transmission electron microscopy of spinal cord spheroids compared to a normally structured axon. Ultrastructurally, spheroids lacked myelin sheaths and contained closely packed accumulations of membrane-bound vacuolar structures. High numbers of vacuoles were rimmed by a double layered membrane separated by an electron-lucent cleft and defined as autophagosomes (insert; **A**). The axons of a healthy, age matched Beagle dog is surrounded by a thick myelin sheath (arrowhead). Isolated vacuolar structures, interpreted as mitochondria (arrow) are present between neurofilaments (**B**). (A) Bar: overview: 1 μm; insert 0.5 μm. (B) Bar: 2.3 μm.

Transmission electron microscopy of the spheroids revealed intraaxonal accumulations of closely packed vacuoles rimmed by two parallel membrane layers separated by an electron-lucent cleft, consistent with the morphology of autophagosomes (**Fig 4A**), **[20].** Furthermore, a loss of the myelin sheets around the axonal dilatations was present. In the axons of a control animal, only single vacuolar structures, interpreted as mitochondria are found (**Fig 4B**).

**Fig 5:**
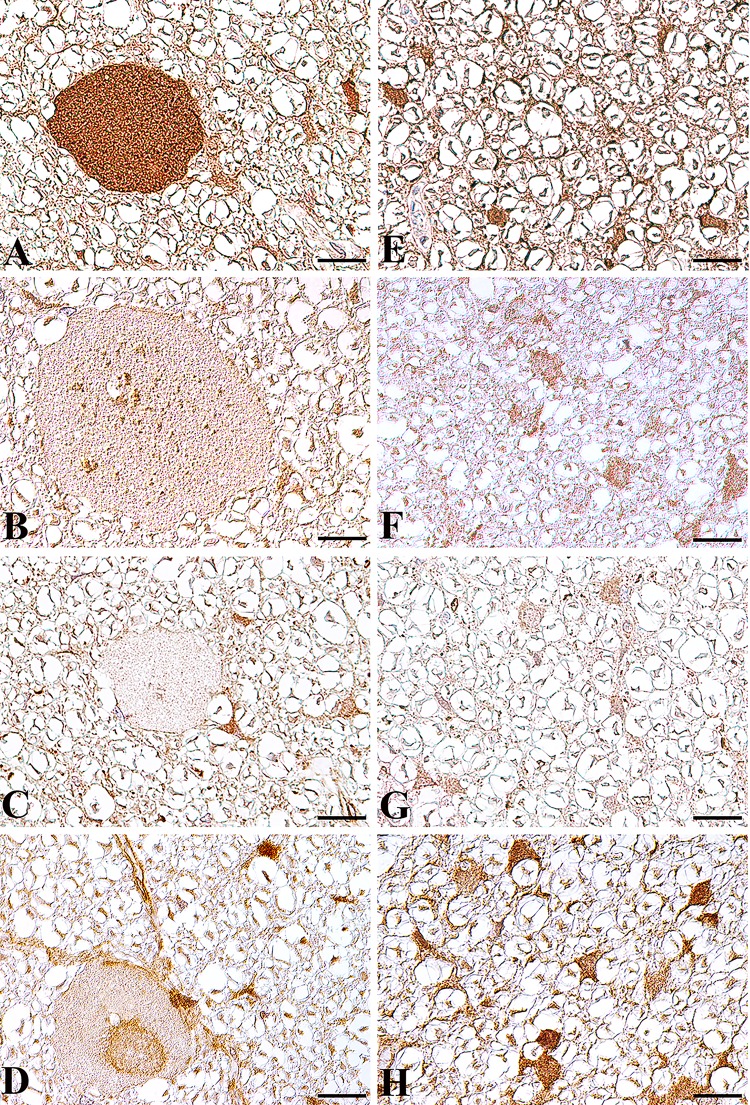
Expression of autophagosome, lysosome and late endosome associated proteins in the spinal cord white matter of an NAD affected Spanish water dog (A-D) and an age matched control Beagle dog (E-H). **(A)** In the affected Spanish water dog, immunohistochemistry using a LC3B specific antibody demonstrated an autophagosome accumulation within spheroids. Only few LAMP2 positive lysosomes **(B)** and RAB7 stained late endosomes **(C)** are present within spheroids. No TECPR2 accumulations are found within spheroids **(D).** In diseased Spanish water dogs, LC3B, LAMP2 and TECPR2 are strongly expressed in glial cells and axons without spheroid formation. In the age matched healthy Beagle dog, LC3B **(E)**, LAMP2 **(F)** and TECPR2 **(H)** positive staining is found in axons and glial cells. RAB 7 **(G)** positive staining is restricted to glial cells. Bar: 20 μm. A-H: Nomarski differential interference-contrast optic.

Immunohistochemistry using antibodies against the autophagosomal protein microtubule-associated protein 1A/1B-light chain 3 B (LC3B), the lysosome-associated membrane protein 2 (LAMP2), and the late endosome marker RAB7 confirmed an accumulation of autophagosomes within spheroids **(Fig 5A)**, whereas only few lysosomes (**Fig 5B**) and late endosomes **(Fig 5C)** are present. Using an antibody directed against human TECPR2, no TECPR2 expression was found within spheroids (**Fig 5D**), whereas neurons and glial cells stained positive in affected and control animals (**S4 Fig**). This suggests that the TECPR2 mutation does not affect TECPR2 expression, but implicates impaired TECPR2 function. However, the specificity of the TECPR2 antibody used was not tested by western blot.

## Discussion

**Fig 6:**
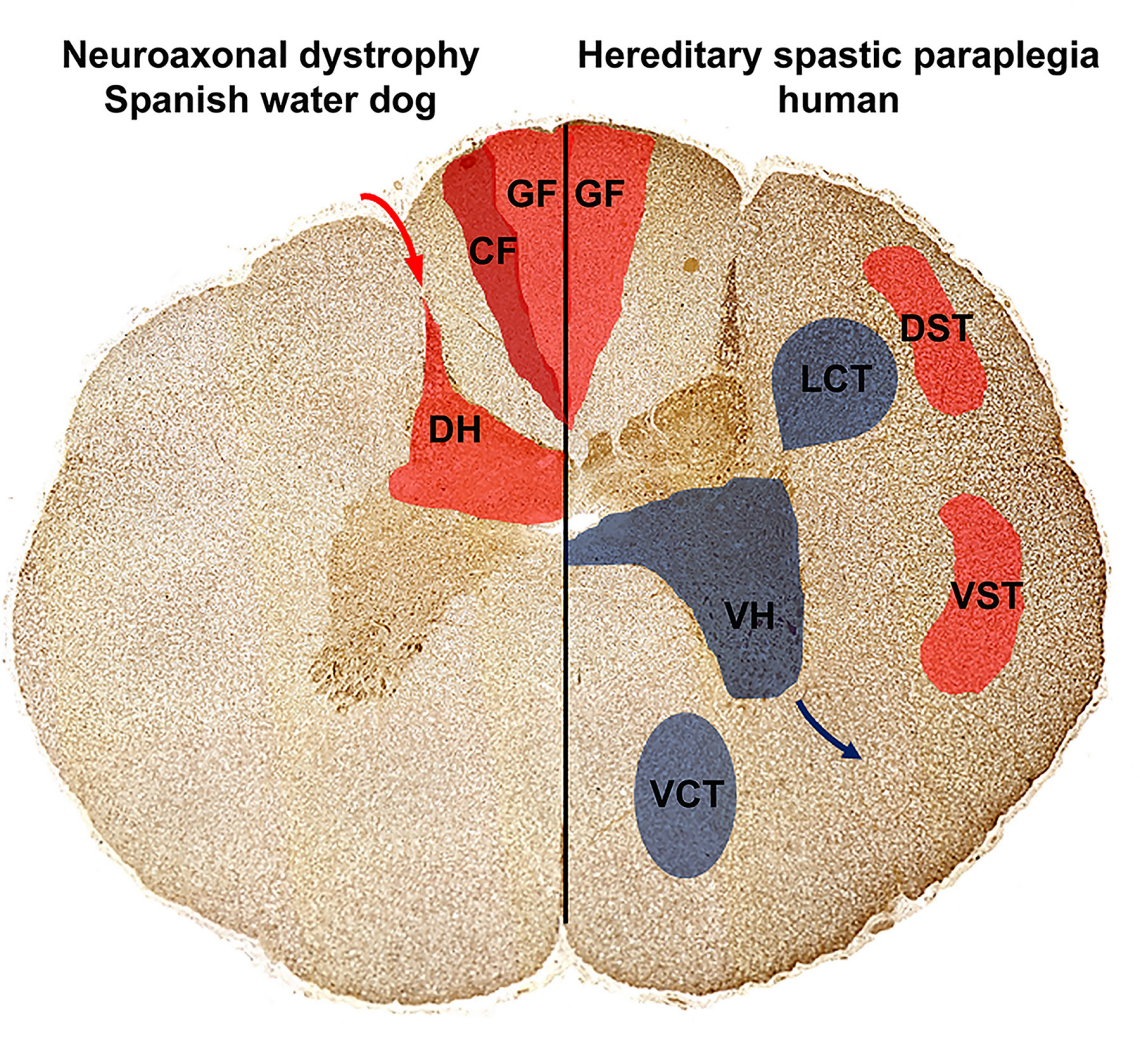
Distribution of cervical and thoracic spinal cord spheroids in Spanish water dogs with NAD compared to mostly affected areas in human hereditary spastic paraparesis (HSP). In NAD affected Spanish water dogs (left), spheroids and neuronal loss were restricted to the sensory, ascending pathways localized to the grey matter of the spinal cord dorsal horn and single large spheroids were detected within the cuneate and gracile fasciculus. This might explain the clinical signs as gait disturbances, proprioceptive deficits, decreased spinal reflexes and urinary incontinence. In other human forms of NAD, HSP and *Pla2g6* knock-out mice (right), spinal cord spheroid formation is accentuated in the sensory pathways including the gracile fasciculus as well as the corticospinal tracts. Furthermore, also the descending motor pathways including the ventral horns as well as the descending pyramidal tracts are affected. Note that spinal cord histology of TECPR2 associated HSP in humans is unknown. Ascending, sensory pathways (red; transmission of sensory signals from the periphery (red arrow) via dorsal horn (DH) towards the brain): Dorsal funiculus composing of gracile fasciculus (GF) and cuneate fasciculus (CF); Spinocerebellar tracts with: dorsal spinocerebellar tract (DST) and ventral spinocerebellar tract (VST); Descending, motor pathways (blue; signal transmission via the ventral horn (VH) neurons towards the muscles; blue arrow): Pyramidal tracts with lateral corticospinal tract (LCT) and ventral corticospinal tract (VCT).

The positional cloning study identified a missense mutation in *TECPR2* as the very likely cause for a previously uncharacterized juvenile-onset form of canine NAD in Spanish water dogs. The affected TECPR2 protein is involved in autophagy, but its detailed function in this pathway is largely unknown [21], [22]. A recent study described a *TECPR2* mutation causing hereditary spastic paraparesis (HSP) in humans, designated as SPG49 (OMIM 615031), [21]. Human patients affected by the *TECPR2* mutation presented with hypotonia during their second year of life and some individuals developed a progressive spasticity until the end of the first decade of life [21]. Histopathology of the human SPG49 patients was only performed from muscle biopsies that were unremarkable similar to the absence of lesions in the muscles from NAD affected dogs [21]. Clinically affected diseased dogs displayed ataxia and paresis characterized by loss of muscle tone and decreased to absent patellar reflexes with no signs of spasticity or muscle atrophy. The distribution of the pathologic lesions was widespread in the CNS and involvement of the afferent pathways in the spinal cord was consistent with the clinical picture indicating predominantly sensory deficits (**Fig 6**). However, all affected dogs in this study were euthanized and the natural progression of NAD in the Spanish water dog needs to be further studied and characterized. The neuropathology of SPG 49 patients is not yet described. However, in the spinal cord of different murine HSP and various types of human HSP autopsy cases, lesions were consistently found in the gracile fasciculus of the cervical spinal cord, but also in the corticospinal tract and descending motor pathways of the thoracic segment [16]. The loss of motor signal transmission from the brain to the body periphery via the descending motor pathways and the spinal cord ventral horn neurons results in loss of motor functions, respectively, as muscle weakness and/or spasticity due to increased spinal reflexes **(Fig 6)** [16].

Interestingly, similar clinical findings and spinal cord lesions corresponding to NAD in the Spanish water dog have been described in humans. One human report describes tetraparesis, hyporeflexia and visual disturbances with disease onset at 18 months of age. [23]. Histopathology revealed absence of iron accumulations, spheroid formation accentuated in the brain stem and spinal cord, restricted to the dorsal horns, whereas peripheral nerves were unaffected [23]. This case was described as an atypical form of NAD, but the genetic association is unknown. Consequently, TECPR2 might represent a potential candidate gene for late infantile or juvenile cases of NAD in humans that are not associated with a mutation in *PLA2G6* and are characterized by the lack of iron accumulations, absence or progressive development of spasticity, visual disturbances and absence of peripheral nerve lesions. In this regard, it has to be considered that *PLA2G6* associated NAD involves the dorsal and ventral horns of the spinal cord as well as peripheral nerves, as demonstrated in humans and murine *PLA2G6* models (http://www.informatics.jax.org/disease/256600), [24], [25], [26].

Except of cases with underlying *PLA2G6* mutations the accumulation of iron in defined brain regions is frequently present in human NAD [27]. Iron deposition was not reported in SPG49 patients comparable to NAD affected Spanish water dogs [21]. In humans, it is suggested that iron deposition may be lacking in the early phase of neurodegeneration with brain iron accumulation (NBIA) [1], [28]. Since the NAD affected Spanish water dogs were euthanized aged between 1 and 2 years, iron depositions at later stages of the disease cannot be excluded. Recently, a novel NBIA type associated with a mutation in *WDR45* was reported and defined as “beta-propeller protein-associated neurodegeneration” (BPAN) [8], [29]. The WDR45 protein possesses such as TECPR2 an N-terminal WD domain and is suggested to regulate autophagosome accumulation [30]. Necropsy of a BPAN patient revealed large numbers of spheroids and prominent iron deposits [29]. The presence of spheroids implicating axonal degeneration and the absence of iron deposits in NAD affected Spanish water dogs and also *Atg7* knock-out mice suggests that the iron deposits represent a secondary event in autophagy related neurodegeneration [31].

On the molecular level, SPG49 was associated with a single base deletion within exon 16 of *TECPR2* that introduces a frame shift, results in a premature stop codon and leads to subsequent proteasomal degradation of the truncated protein [21]. In the diseased Spanish water dogs, TECPR2 accumulations were not detected in spheroids using immunohistochemistry. This finding suggests that the neuropathology, especially the axonal swellings and vacuolar accumulations, was not the consequence of axonal TECPR2 aggregations, but result from deficits in TECPR2 function. The functional relevance of the arginine residue mutated in canine NAD is supported by its evolutionary high interspecies conservation, even in nematodes and the fruit fly paralog TECPR1. Similar to TECPR1, TECPR2 was suggested to regulate autophagosome maturation and accumulation [21], [22], [32]. Spheroids in NAD affected Spanish water dogs contained high proportions of autophagosomes, whereas only few late endosomes and lysosomes were found. This observation supports that TECPR2 regulates autophagosome accumulation also in neurons. In general, this finding may result from increased autophagosome production, disturbances of vesicle transport and/or impairments of autophagosome fusion with late endosomes or lysosomes. TECPR1 was demonstrated to provide autophagosome-lysosome fusion [32]. However, in neurons the fusion between autophagosomes and lysosomes is suggested to occur in the soma [33]. Histology of NAD affected Spanish water dogs identified primary axonal lesions, whereas only a few neuronal cell bodies were affected. Consequently, disturbed autophagosome-lysosome fusion seems not the main pathomechanism in *TECPR2* mutation associated autophagosome accumulation. Additionally, neuronal loss and spheroid formation were restricted to specific brain and spinal cord localizations. This finding indicates that specific neuronal subpopulations depend on TECPR2 function or autophagy in general, whereas compensatory mechanisms may exist in other neurons.

NAD and HSP both represent neurodegenerative diseases with overlapping clinical and histopathological features. A distinct classification without knowledge of the underlying genetics remains challenging [1]. After the identification of *WDR45* mutations linking autophagy and neurodegeneration, the association of other NAD or HSP related genes including *SPG11* (OMIM 604360), *SPG15* (OMIM 270700), and *SPG60* (OMIM 612167) was demonstrated [8], [34], [35], [36], [37]. Further NAD and HSP associated genes encode proteins involved in mitochondrial and lipid metabolism, axonal transport, endoplasmatic reticulum (ER) morphology, ER protein quality control, ER-associated protein degradation as well as endosome or membrane trafficking and vesicle formation [7], [16]. Interestingly, all these pathways are closely linked to autophagy [38], [39], [40], [41], [42]. Therefore, autophagy modulation has to be anticipated for numerous other NAD, NBIA, and especially SPG associated genes. Thus, HSP and NAD pathogenesis may primary involve disturbances of different subcellular compartments, which generally affect the autophagy pathway. Consequently, the analysis of the functions of NAD, NBIA, and SPG associated proteins as well as their interactions might provide critical clues for a deeper understanding of neuronal autophagy and autophagy associated neurodegeneration.

Summarized, a disease causing mutation in the canine *TECPR2*, a known human HSP associated gene, was identified as the highly likely cause of NAD in Spanish water dogs. In addition, *TECPR2* may represent a suitable candidate for canine and human NAD cases with unknown genetic etiology. However, the identification of new NAD and HSP candidate genes enables an early diagnosis but therapy is still restricted to symptomatic and palliative treatments. This study shows that the identification of disease associated mutations in dogs adds valuable insights into the understanding of specific pathomechanisms of similar diseases in humans. Specifically canine axon length makes dogs a more suitable translational model for human studies in comparison to mice. Therefore, NAD affected Spanish water dogs represent a valuable large animal model enabling not only detailed studies on TECPR2 function but also on the relevance of autophagy in neuronal maintenance.

## Materials and Methods

### Ethics Statement

Affected Spanish water dogs were examined with the consent of their owners and under ethical approval from the Uppsala Animal Experiment Ethics Board, Sweden. The tissue of the control Beagle dogs used for electron microscopy and immunohistochemistry derived from the archive of the Department of Pathology, University of Veterinary Medicine Hannover, Germany.

### Clinical characterization of NAD

Three affected dogs underwent a complete neurological examination according to standardized protocols by a veterinary neurologist because of gait abnormalities, behavioral changes, and incontinence with an insidious onset between six and eleven months of age. The fourth dog was examined by another veterinarian; however a video-recording showing the dog’s gait was available for evaluation. A complete blood count and serum chemistry profile were analyzed in all affected dogs. A cerebrospinal fluid sample was collected from the cisterna magna in two of the affected dogs. One dog was anesthetized with propofol intravenously for inspection of the vocal folds. Due to the progression of clinical signs affected dogs were euthanized within a few months to over a year after clinical signs became obvious to the owners.

### Pathological examination

Complete necropsies were performed of three affected dogs. In the fourth case, only the brain was available for examination. Pathological examination was performed according to standardized procedures at the Department of Pathology, Pharmacology and Toxicology, Swedish University of Agricultural Sciences, Uppsala. Representative samples of all organs and tissues were collected, fixed in 10% neutral buffered formalin, and routinely processed in paraffin wax. Five μm thick tissue sections were stained with haematoxylin and eosin.

### Electron microscopy

For transmission electron microscopy, sections of formalin fixed cervical spinal cord and brain stem were fixed for 24 h in 2.5% glutaraldehyde solution, rinsed with 0.1% sodium cacodylate buffer (pH 7.2), postfixed in 1% osmium tetroxide, and embedded in EPON 812 (SERVA Electrophoresis GmbH, Heidelberg, Germany) as described [43]. Sections were stained with lead citrate and uranyl acetate and investigated using an EM 10c (Carl Zeiss Jena GmbH, Oberkochen, Germany).

### Immunohistochemistry

Immunohistochemistry was performed as described [44, 45] on formalin-fixed, paraffin-embedded tissue sections using rabbit polyclonal antibodies against LAMP2 (ab18528, dilution 1: 100, Abcam, Cambridge, United Kingdome), LC3B (ab48394, dilution 1:800, Abcam), RAB7 (ab77993, dilution 1:50, Abcam) and the human TECPR2 specific antibody (HPA000658; dilution 1:50; Sigma-Aldrich Chemie GmbH, Taufkirchen, Germany). PBS was used for antibody dilution. To block endogenous peroxidase, tissue sections were treated with 0.5% H_2_O_2_ diluted in 80% ethanol, heated in sodium citrate buffer, and incubated with 20% goat serum to block non-specific binding sites. Subsequently, sections were incubated with the primary antibodies overnight at 4°C. Control sections were incubated with normal rabbit serum (R4505; dilution: 1:3000, Sigma-Aldrich). Biotinylated goat-anti-rabbit IgG (BA-1000; dilution: 1:200; Vector Laboratories, Burlingame, CA, USA) was used as secondary antibody. As detection system, the avidin-biotin-peroxidase complex (ABC) method (Vector Laboratories) was applied using 3,3′-diamino-benzidine tetrahydrochloride (DAB) as chromogen. Sections were slightly counterstained with Mayer's hematoxylin and mounted.

### Animals

Blood samples from four NAD-affected Spanish water dogs, their healthy littermates (n = 9), sires (n = 2), dams (n = 3), a single grand sire, as well as of 263 unrelated control dogs of this breed were collected. Genomic DNA was isolated from blood using the Nucleon Bacc2 kit (GE Healthcare, Glattbrugg, Switzerland) and the DNeasy blood and tissue kit (Qiagen, Hombrechtikon, Switzerland) according to manufacturer instructions. DNA samples of phenotypically related dog breeds like Barbet (n = 20), Briard (n = 5), Cão da Serra de Aires (n = 15), Cão de Água Português (Portuguese Waterdog) (n = 3), Lagotto Romagnolo (n = 67), Standard Poodles (n = 12), as well as 146 control dogs of various other breeds were collected during the course of other research projects at the Institute of Genetics, University of Bern, Switzerland.

### Genetic mapping

Genomic DNA from four cases and thirteen related phenotypically healthy dogs was genotyped on the Illumina CanineHD BeadChip (Illumina, San Diego, USA) containing more than 170,000 evenly spaced and validated SNPs derived from the CanFam3.1 assembly. To identify extended homozygous regions with allele sharing across cases and controls, the following PLINK software commands were used:—maf 0,—max-maf 1.0,—geno 0.01,—hwe 0,—mind 0.15,—homozyg,—homozyg-match 1,—homozyg-group [46]. All given positions correspond to the CanFam3.1 genome assembly. Multipoint parametric linkage analyses were performed with MERLIN software version 1.1.2 [47]. For parametric linkage, LOD scores were calculated under both, homogeneity and heterogeneity, under the assumption of NAD segregating as a biallelic autosomal recessive trait, with complete penetrance. The frequency of the disease allele in the considered population is unknown and there is no data available that would make it possible to estimate the frequency in a reliable manner. For the calculations a frequency of 0.01 for the mutated allele was assumed.

### Whole genome sequencing of one affected Spanish water dog

A standard fragment library was prepared with 300 bp insert size and one lane of illumina HiSeq2000 paired-end reads (2×100 bp) was collected. 189 million 100 bp paired-end reads were collected corresponding to roughly 10x coverage of the genome. The reads were mapped to the dog reference genome using the Burrows-Wheeler Aligner (BWA) version 0.5.9-r16 with default settings [48]. PCR duplicates were labelled with Picard tools (http://sourceforge.net/projects/picard/). Local realignment was performed using the Genome Analysis Tool Kit (GATK version v2.3–6) to perform and to produce a cleaned BAM file [49]. The genome data has been made freely available under accession no. PRJEB7903 at the European Nucleotide Archive. Variant calls were then made with the unified genotyper module of GATK. Variant data for each sample were obtained in variant call format (version 4.0) as raw calls for all samples and sites flagged using the variant filtration module of GATK. Variant calls that failed to pass the following filters were labelled accordingly in the call set: (i) Hard to Validate MQ0 ≥4 & ((MQ0/(1.0 * DP)) >0.1); (ii) strand bias (low Quality scores) QUAL <30.0 || (Quality by depth) QD <5.0 || (homopolymer runs) HRun >5 || (strand bias) SB >0.00; (iii) SNP cluster window size 10. The snpEFF software [50] together with the CanFam 3.1 annotation was used to predict the functional effects of detected variants. The following snpEFF categories of variants were considered as non-synonymous: NON_SYNONYMOUS_CODING, CODON_DELETION, CODON_INSERTION, CODON_CHANGE_PLUS_CODON_DELETION, CODON_CHANGE_PLUS_CODON_INSERTION, FRAME_SHIFT, EXON_DELETED, START_GAINED, START_LOST, STOP_GAINED, STOP_LOST, SPLICE_SITE_ACCEPTOR, SPLICE_SITE_DONOR.

### Mutation analysis of canine *TECPR2*

Primers for the amplification of the *TECPR2* variant (forward GACAGACGGACACCCTGTTC, reverse CAGATCCACCACCCTCAATC) were designed using Primer3 software (http://frodo.wi.mit.edu/cgi-bin/primer3/primer3_www.cgi) after masking repetitive sequences with RepeatMasker (http://www.repeatmasker.org). For sequencing, a 490 bp PCR product was amplified using AmpliTaqGold360 DNA polymerase (LifeTechnologies, Darmstadt, Germany). The subsequent re-sequencing of the PCR products was performed after rAPid alkaline phosphatase (Roche, Basel, Switzerland) and exonuclease I (New England Biolabs, Frankfurt, Germany) treatment with the ABI BigDye Terminator Sequencing Kit 3.1 (LifeTechnologies) on an ABI 3730 genetic analyzer. Sequence data were analyzed with Sequencher 4.9 (GeneCodes, Ann Arbor, USA).

### Protein sequence analysis

Multiple sequence alignment was performed with ClustalW (http://www.ebi.ac.uk/Tools/msa/clustalw2/). GeneBank accession numbers used for DNA, RNA alignments were: i) for TECPR2: NC_006590, XM_005623828.1; XP_005623885.1 (*canis lupus familiaris*). Further accession numbers used for the TECPR2 and TECPR1 protein alignments are listed in **S2 Table**. The Peroxin-23 sequence is available from UniProtKB/Swiss-Prot: Q9VWB0.2 (*Drosophila melanogaster*). Impact of the mutations on the protein stability and structure was predicted with the following software tools: PolyPhen2 [51], NetTurnP [52], NetSurfP [53], the Chou&Fasman secondary structure prediction server CFSSP [54], PoPMuSiC [55], and Phyre2 [56].

## Supporting Information

S1 Fig Iron staining of the basal ganglia/ Globus pallidus of a NAD affected Spanish water dog. Using Turnbull’s blue (**A**) and Prussian blue staining (**B**), no iron deposition was detected in the basal ganglia. Positive control stain (granulation tissue with numerous haemosiderophages) for Turnbull’s blue (**C**) and Prussian blue (**D**). Bar: 20 μm.
(TIF)

S2 Fig Linkage analysis of NAD in Spanish water dogs. Graphical LOD score statistics for NAD are shown per dog chromosome.
(PDF)

S3 Fig Alignment of TECPR2 (sixth propeller domain) and TECPR1 (ninth propeller domain) in vertebrate and non-vertebrate species. The arginine residue that is substituted by a tryptophan (p.R1337W) in NAD affected Spanish water dogs is highly conserved in the TECPR1 orthologs. The homology of the sixth and ninth propeller domain of TECPR2 and TECPR1 further indicates the functional relevance of this C-terminal propeller domain.
(PDF)

S4 Fig TECPR2 immunohistochemistry of the spinal cord using an human TECPR2 specific antibody, the avidin-biotin-peroxidase complex method, the chromogen 3,3′-diamino-benzidine tetrahydrochloride, and Mayer's hematoxylin as counterstain. TECPR2 expression was detected in neurons in the grey matter of affected dogs (**A**) and age-matched control Beagle dogs (**B**). Bar: 20 μm.
(TIF)

S1 Table Classification of inherited or idiopathic neuroaxonal dystrophies in humans. (PDF)

S2 Table GeneBank accession numbers used for the TECPR2 and TECPR1 alignments. (PDF)

S1 Video Clinical presentation of a NAD affected Spanish water dog aged 23 months. (MP4)
