# Supplementary figures and images for: TECPR2 Associated Neuroaxonal Dystrophy in Spanish Water Dogs
Source: PLoS One. 2015 Nov 10;10(11):e0141824. doi: 10.1371/journal.pone.0141824 (PMC4640708; doi:10.1371/journal.pone.0141824)

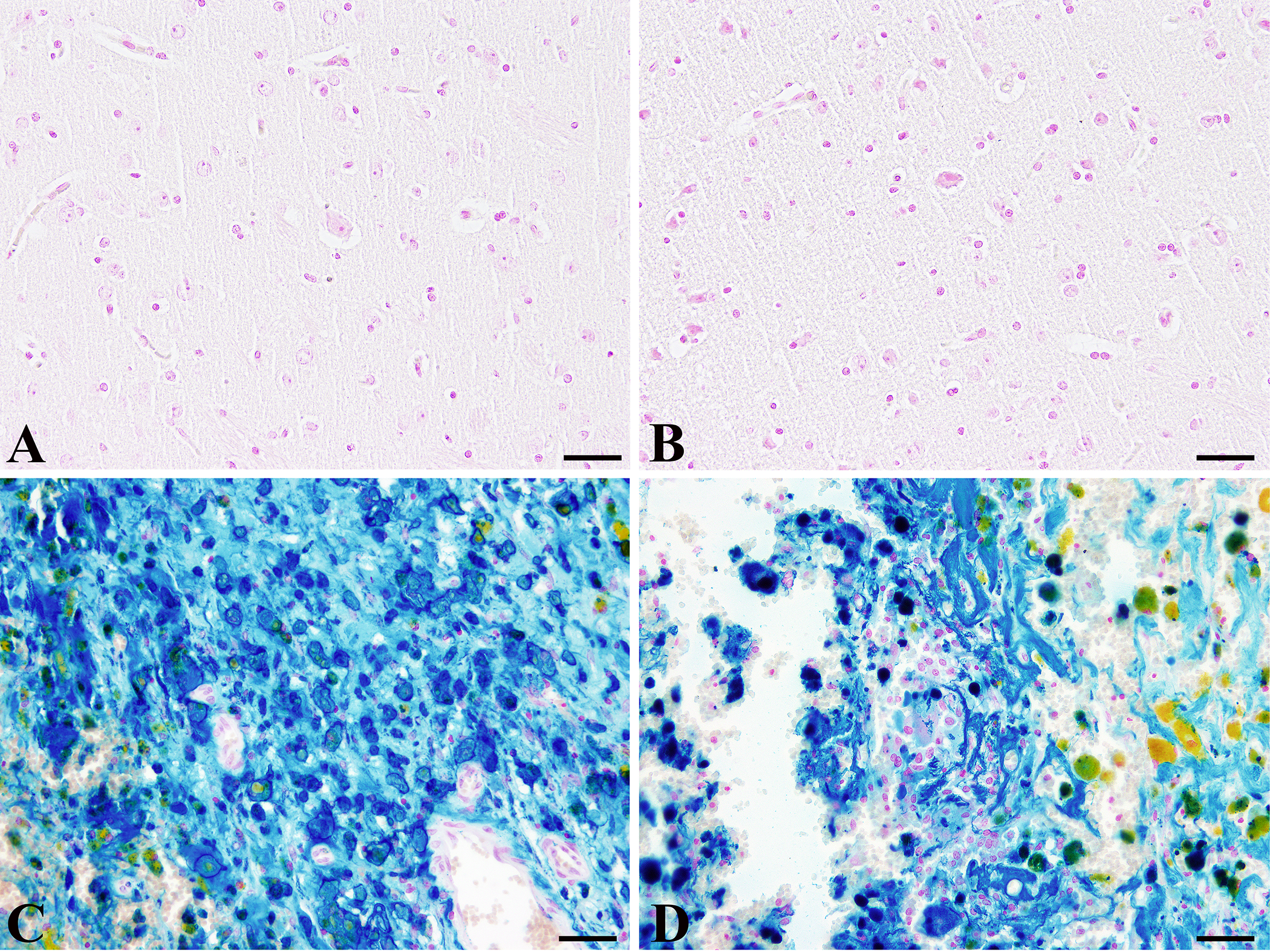

Supplement: S1 Fig — Using Turnbull’s blue (A) and Prussian blue staining (B), no iron deposition was detected in the basal ganglia. Positive control stain (granulation tissue with numerous haemosiderophages) for Turnbull’s blue (C) and Prussian blue (D). Bar: 20 μm. (TIF) [file pone.0141824.s001.tif]

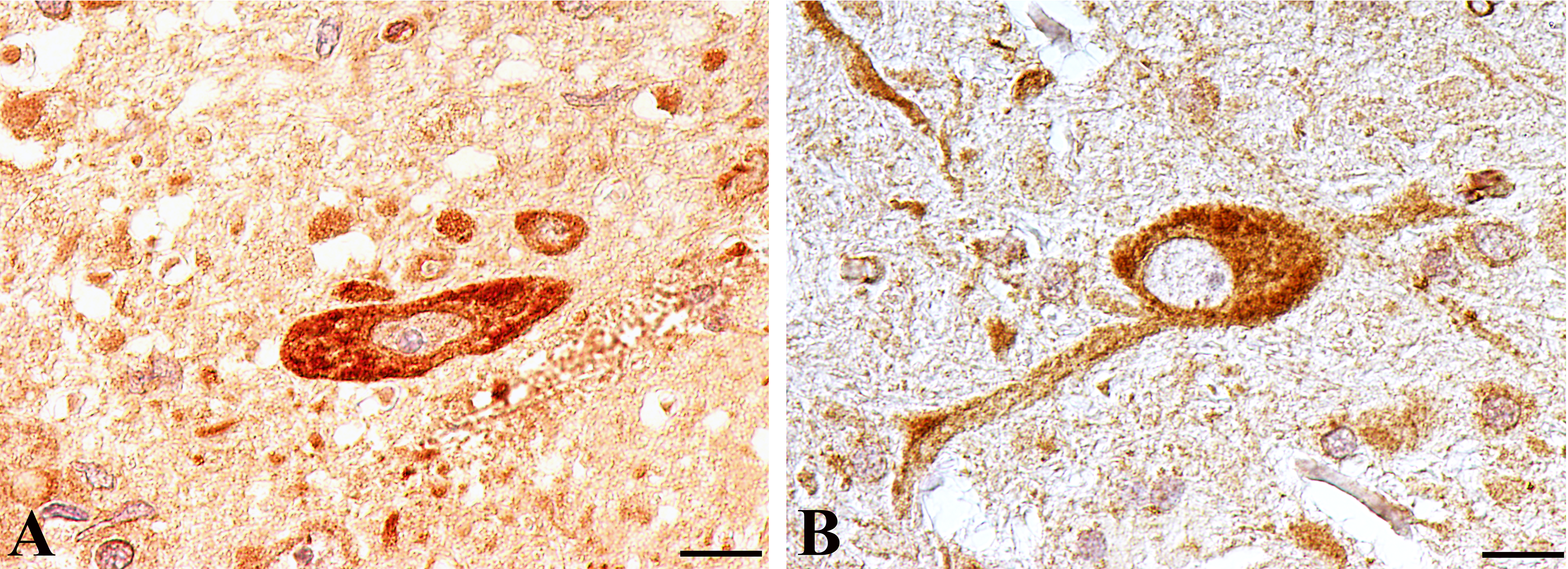

Supplement: S4 Fig — TECPR2 expression was detected in neurons in the grey matter of affected dogs (A) and age-matched control Beagle dogs (B). Bar: 20 μm. (TIF) [file pone.0141824.s004.tif]
