# Supplementary material for: TECPR2 Associated Neuroaxonal Dystrophy in Spanish Water Dogs
Source: PLoS One. 2015 Nov 10;10(11):e0141824. doi: 10.1371/journal.pone.0141824 (PMC4640708; doi:10.1371/journal.pone.0141824)
Supplement: S2 Fig — Graphical LOD score statistics for NAD are shown per dog chromosome. (PDF) [file pone.0141824.s002.pdf]

Parametric Analysis for recessive

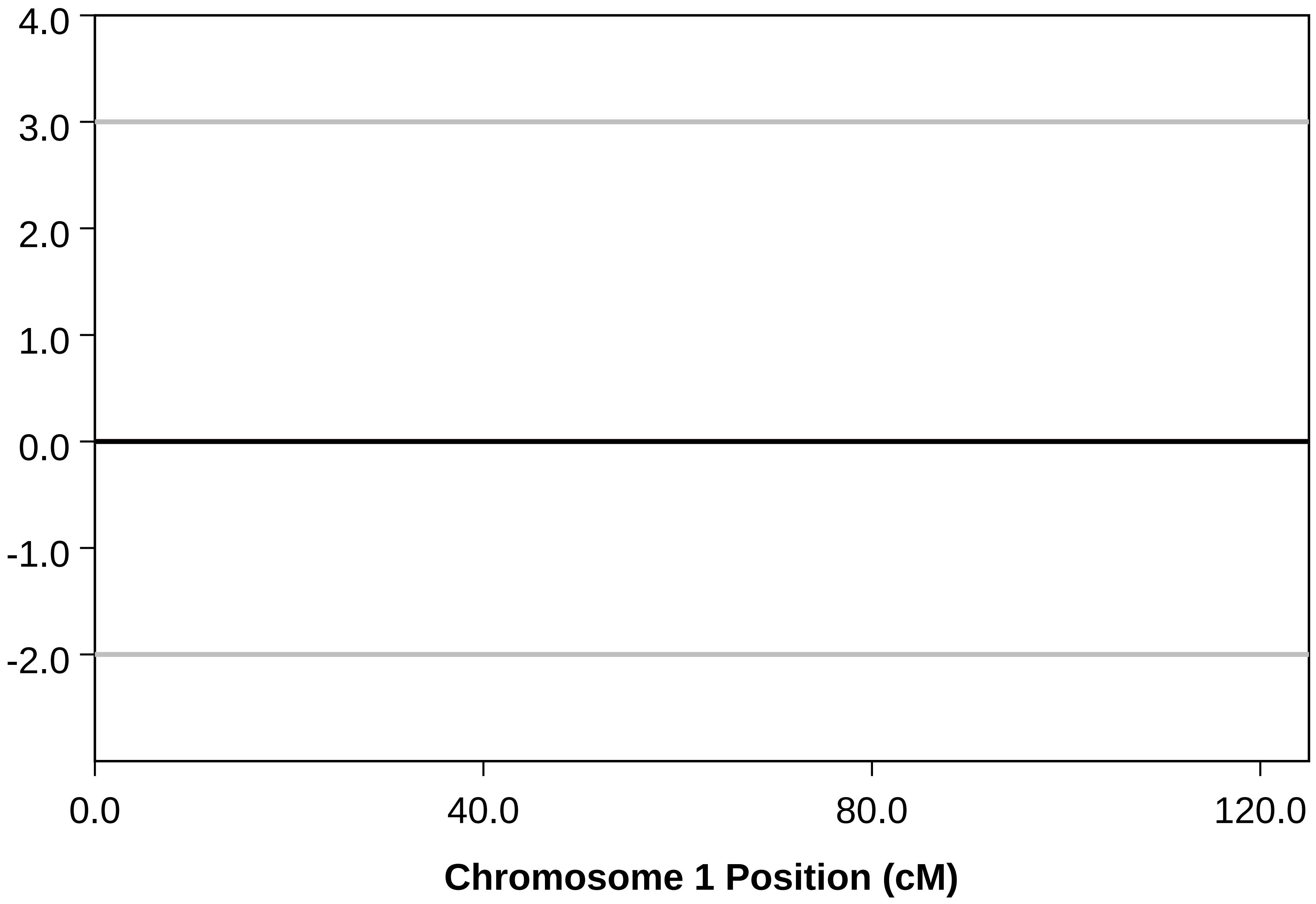

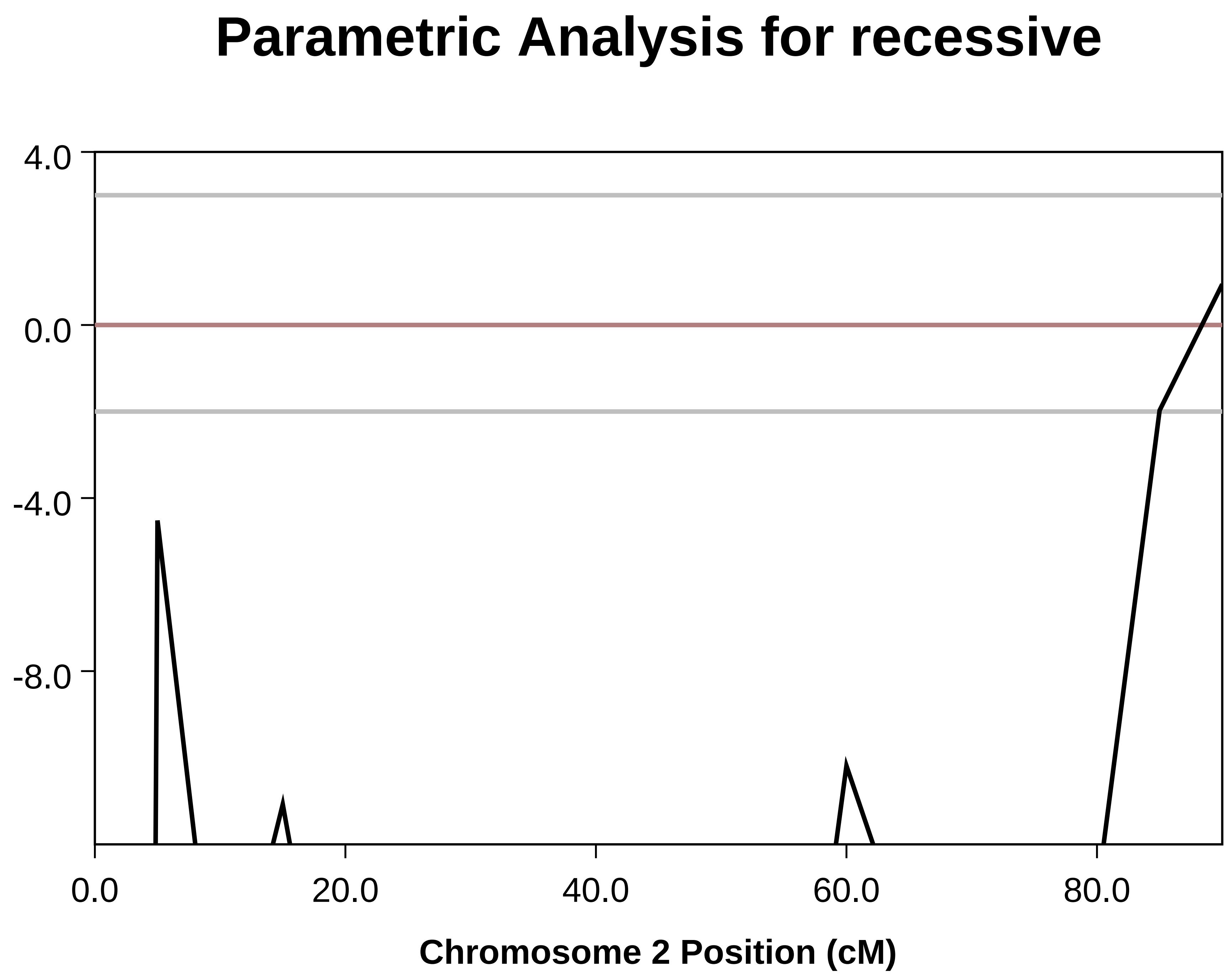
Parametric Analysis for recessive
4.0
0.0
-4.0
-8.0
0.0
20.0
40.0
60.0
80.0
Chromosome 2 Position (cM)

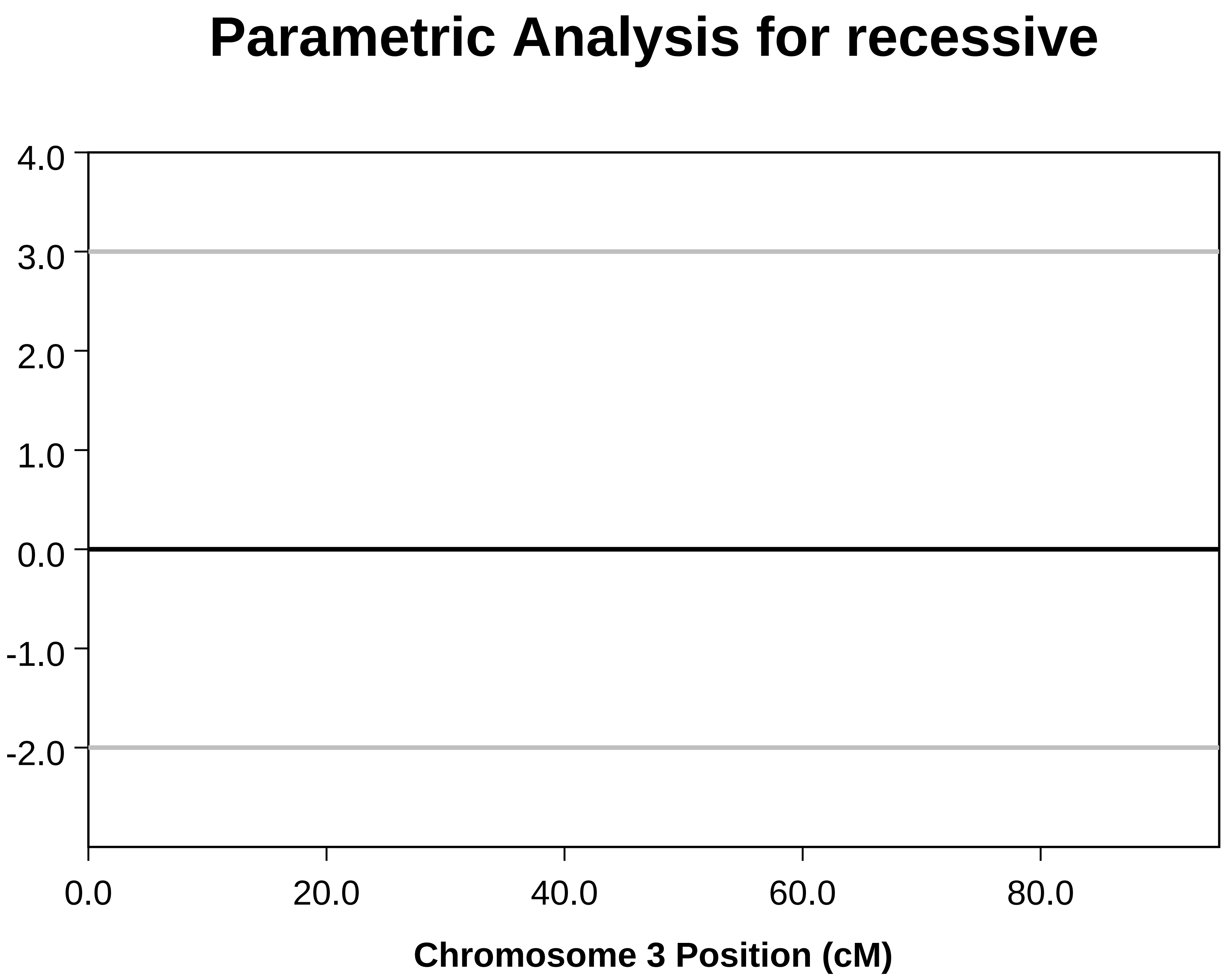
Parametric Analysis for recessive
4.0
3.0
2.0
1.0
0.0
-1.0
-2.0
0.0
20.0
40.0
60.0
80.0
Chromosome 3 Position (cM)

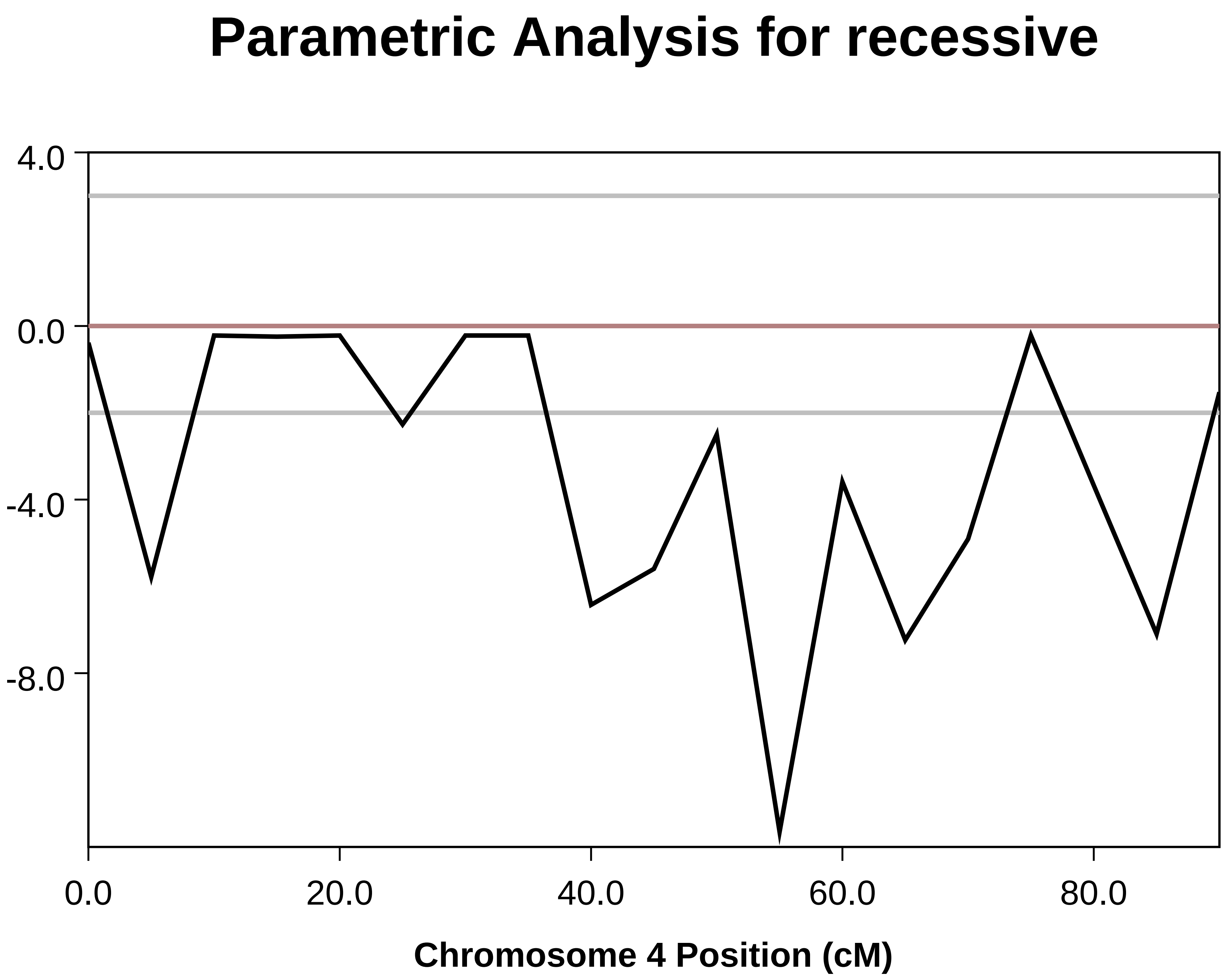
Parametric Analysis for recessive
4.0
0.0
-4.0
-8.0
0.0
20.0
40.0
60.0
80.0
Chromosome 4 Position (cM)

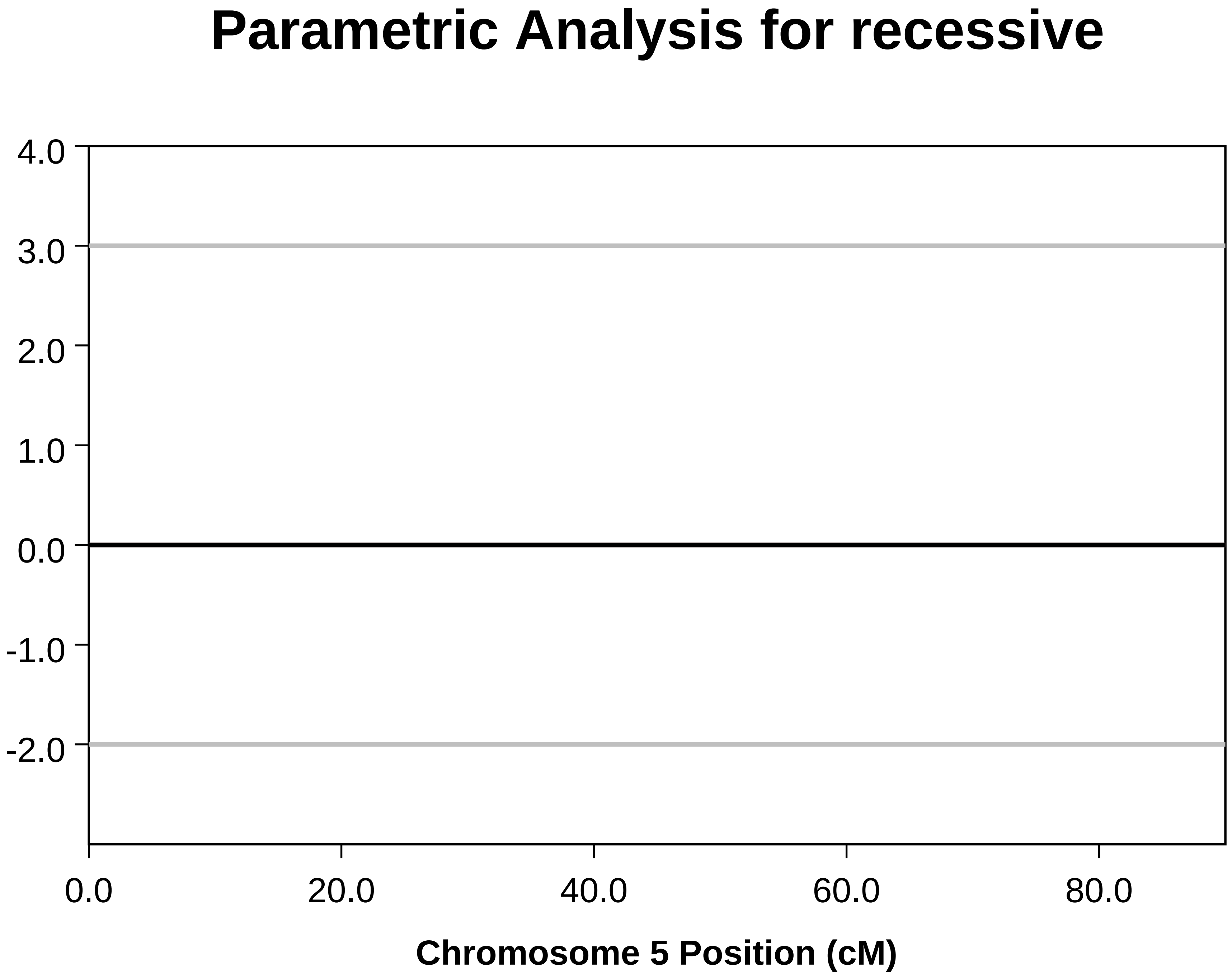
Parametric Analysis for recessive
4.0
3.0
2.0
1.0
0.0
-1.0
-2.0
0.0
20.0
40.0
60.0
80.0
Chromosome 5 Position (cM)

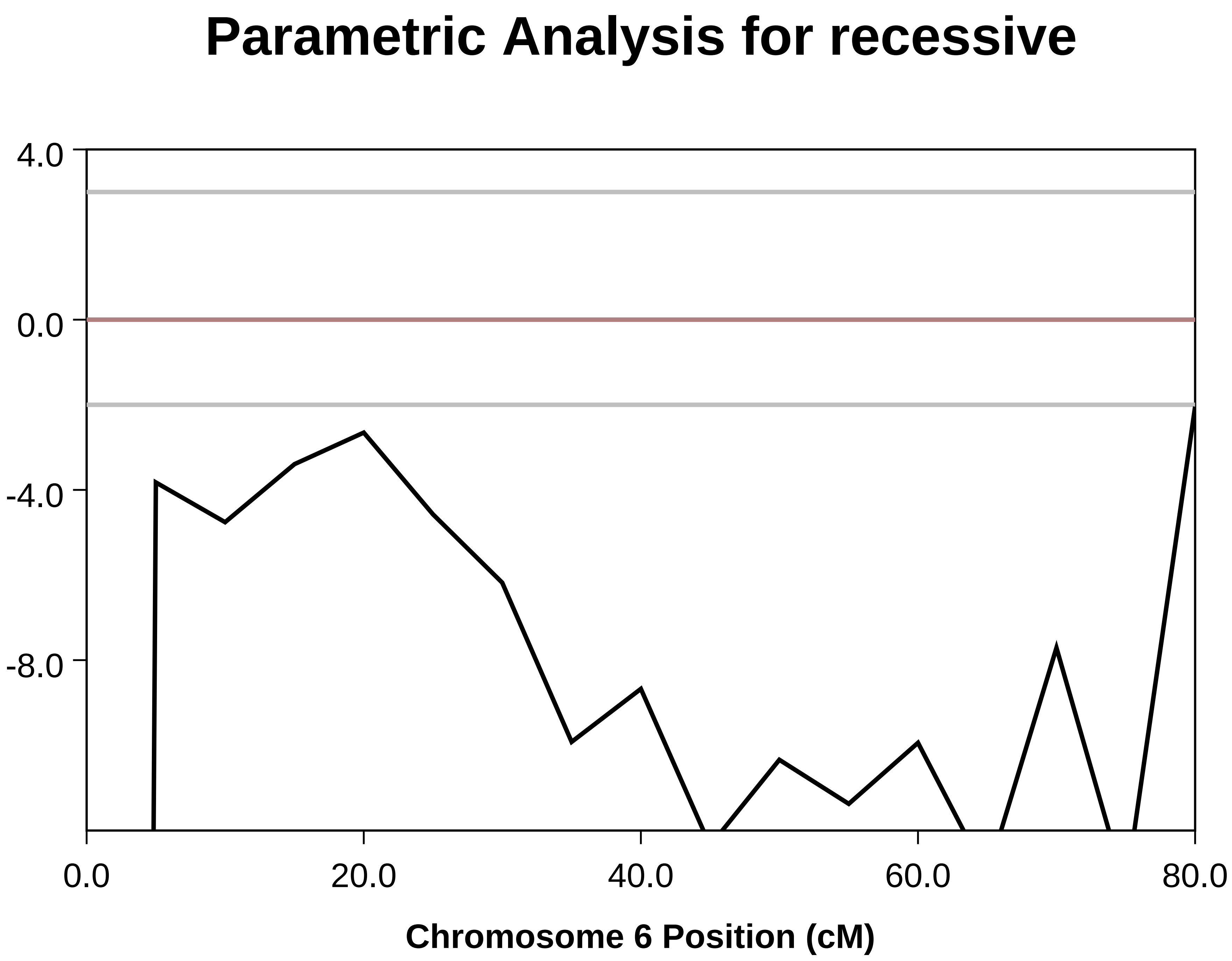
Parametric Analysis for recessive
4.0
0.0
-4.0
-8.0
0.0
20.0
40.0
60.0
80.0
Chromosome 6 Position (cM)

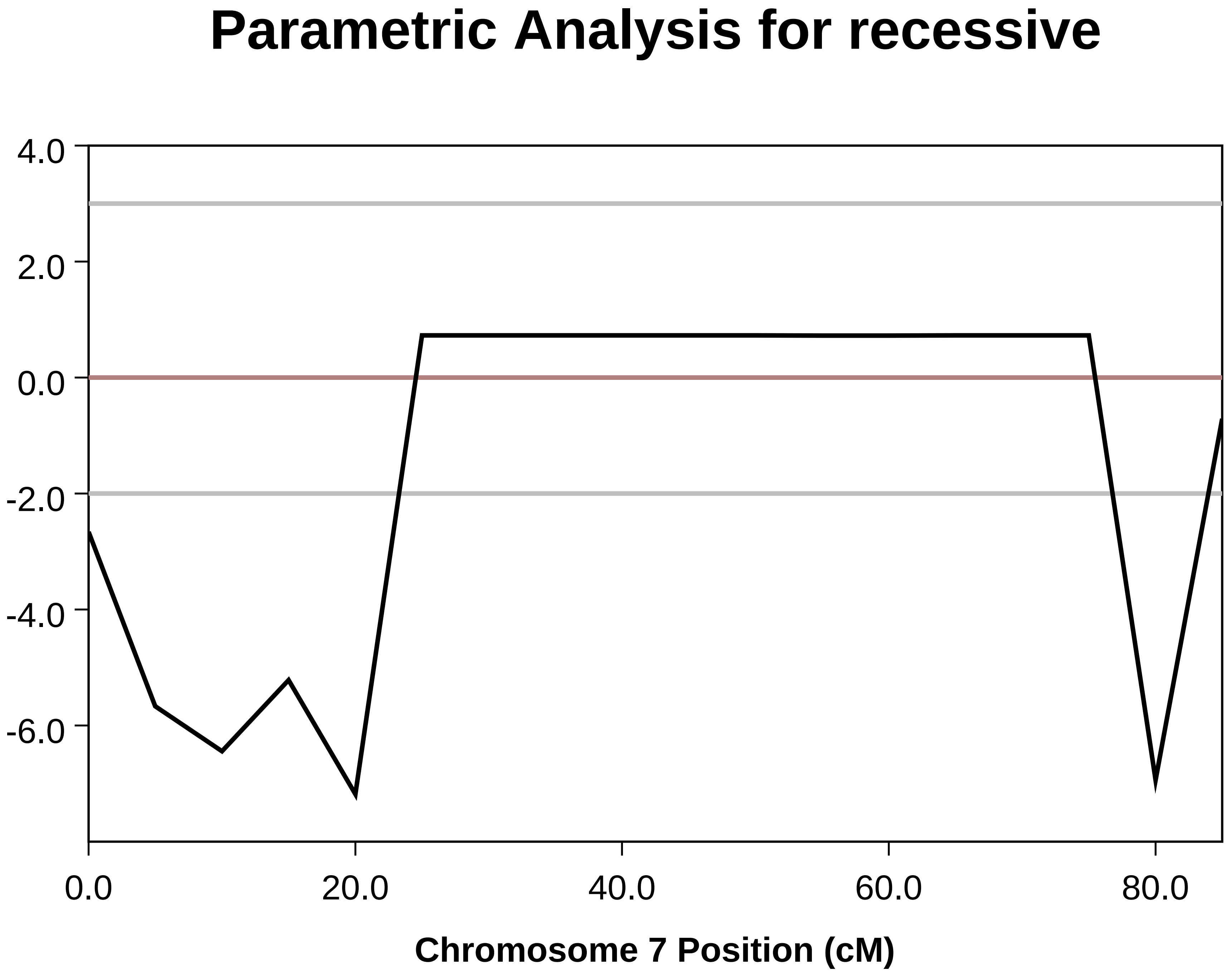

Parametric Analysis for recessive
4.0
2.0
0.0
-2.0
-4.0
-6.0
0.0
20.0
40.0
60.0
80.0
Chromosome 7 Position (cM)

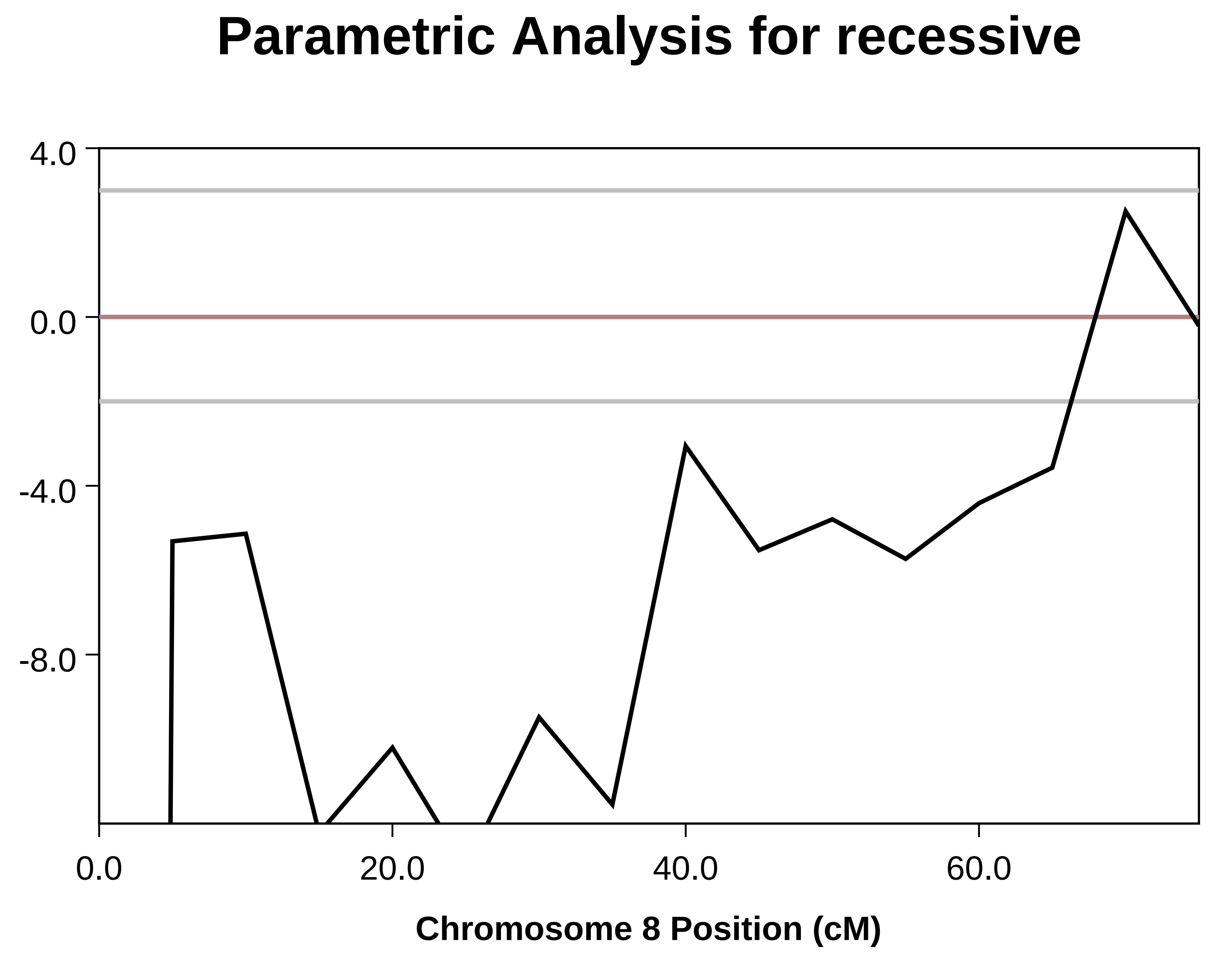
Parametric Analysis for recessive
4.0
0.0
-4.0
-8.0
0.0
20.0
40.0
60.0
Chromosome 8 Position (cM)

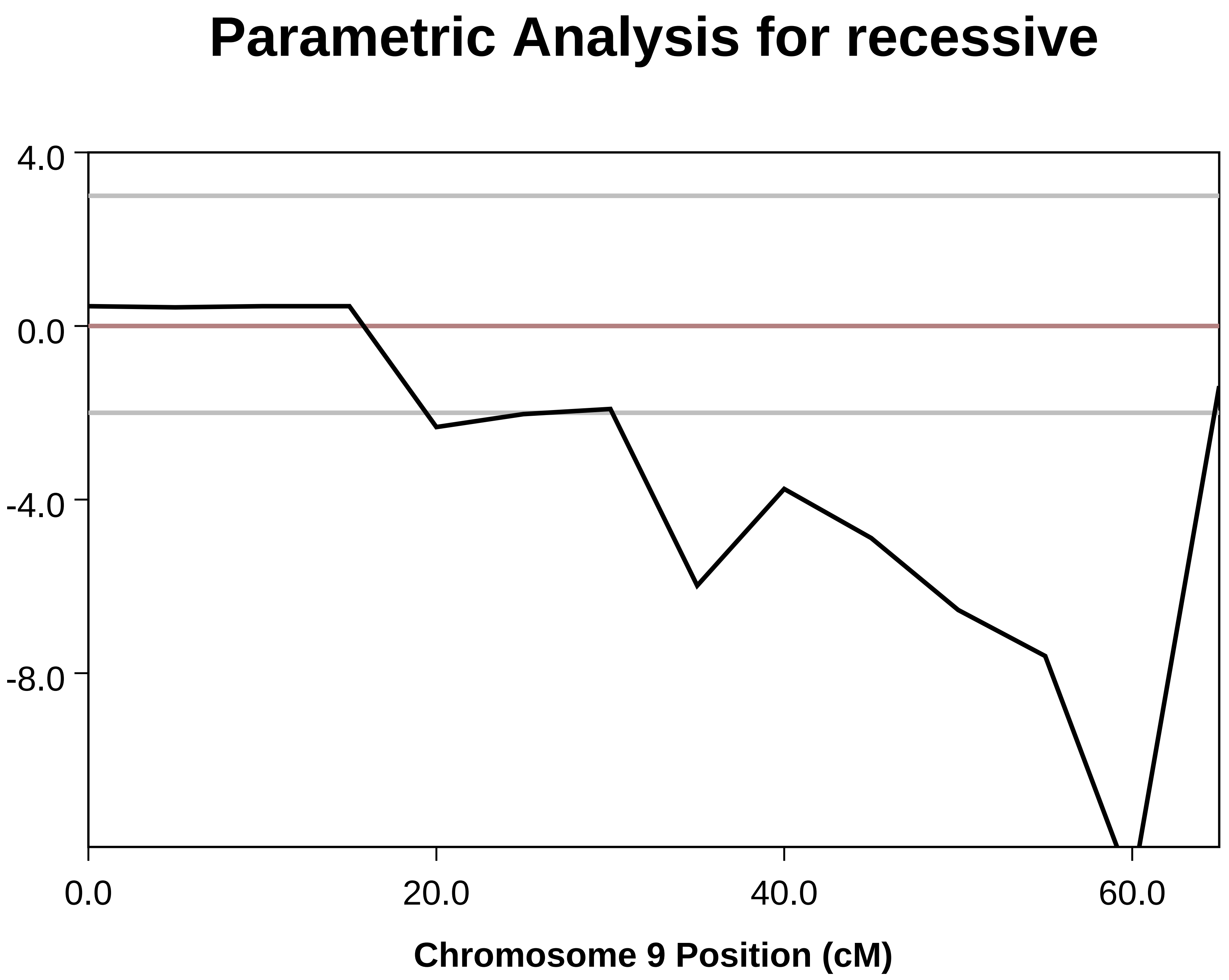
Parametric Analysis for recessive
4.0
0.0
-4.0
-8.0
0.0
20.0
40.0
60.0
Chromosome 9 Position (cM)

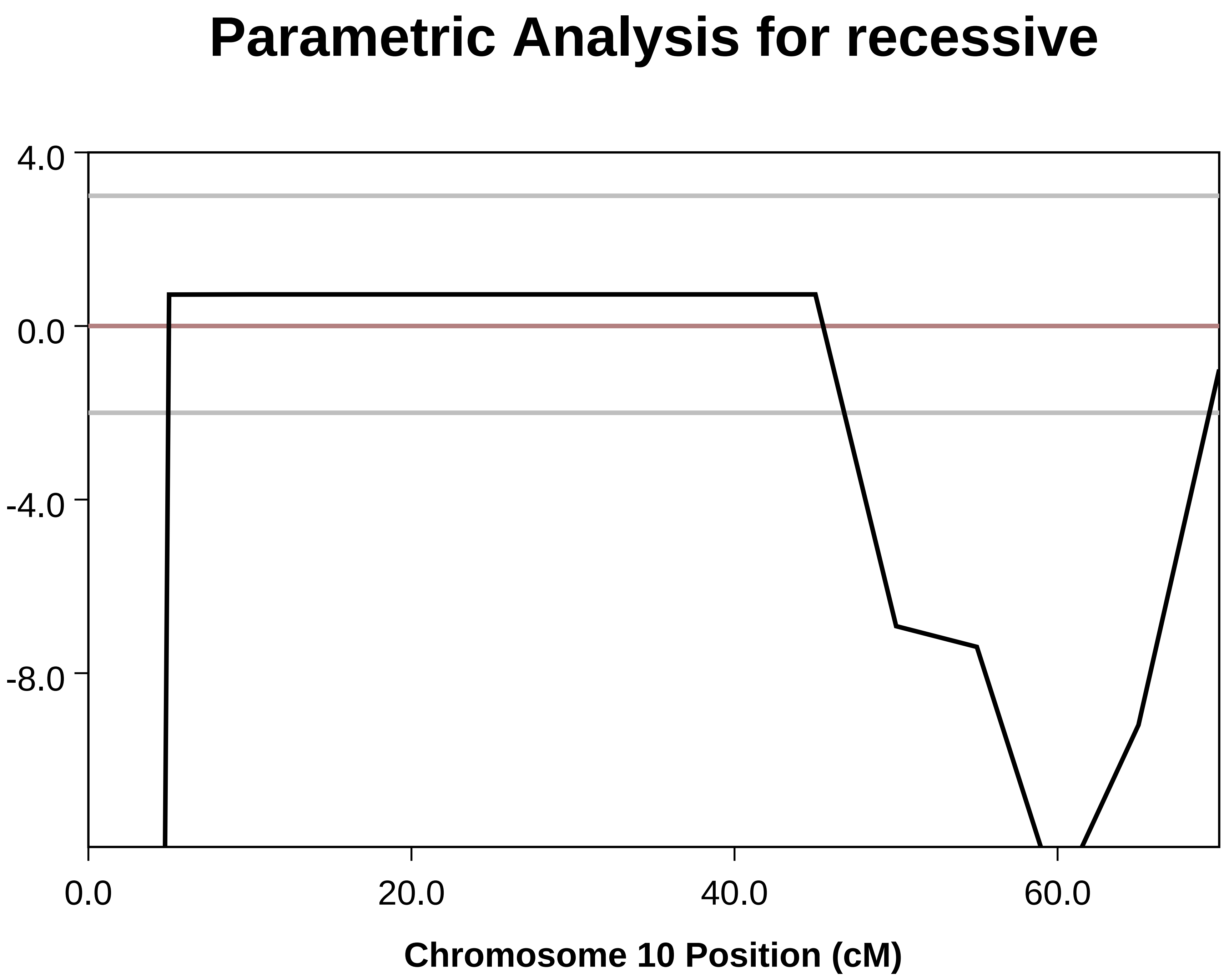

Parametric Analysis for recessive
4.0
0.0
-4.0
-8.0
0.0
20.0
40.0
60.0
Chromosome 10 Position (cM)

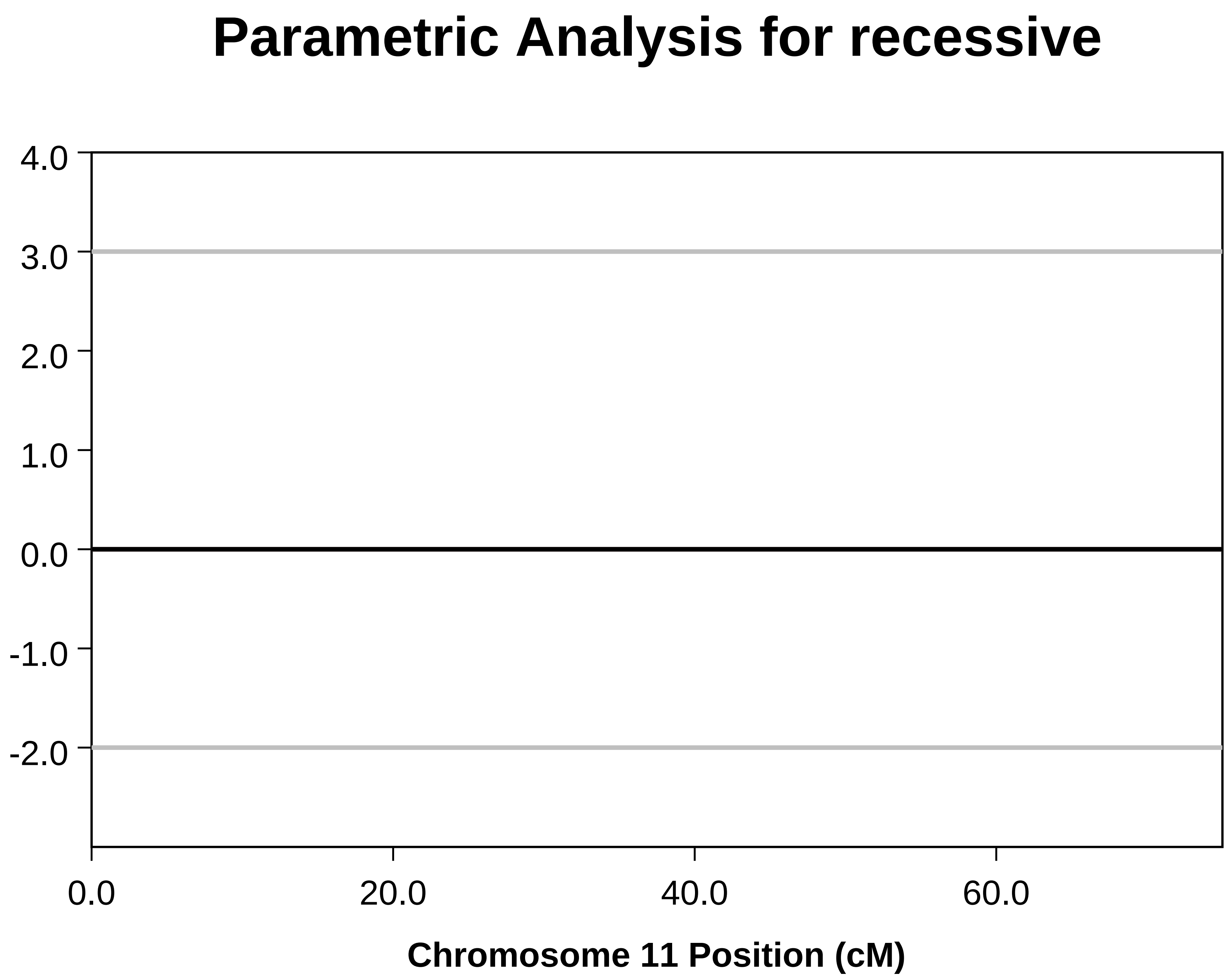
Parametric Analysis for recessive
4.0
3.0
2.0
1.0
0.0
-1.0
-2.0
0.0
20.0
40.0
60.0
Chromosome 11 Position (cM)

Parametric Analysis for recessive

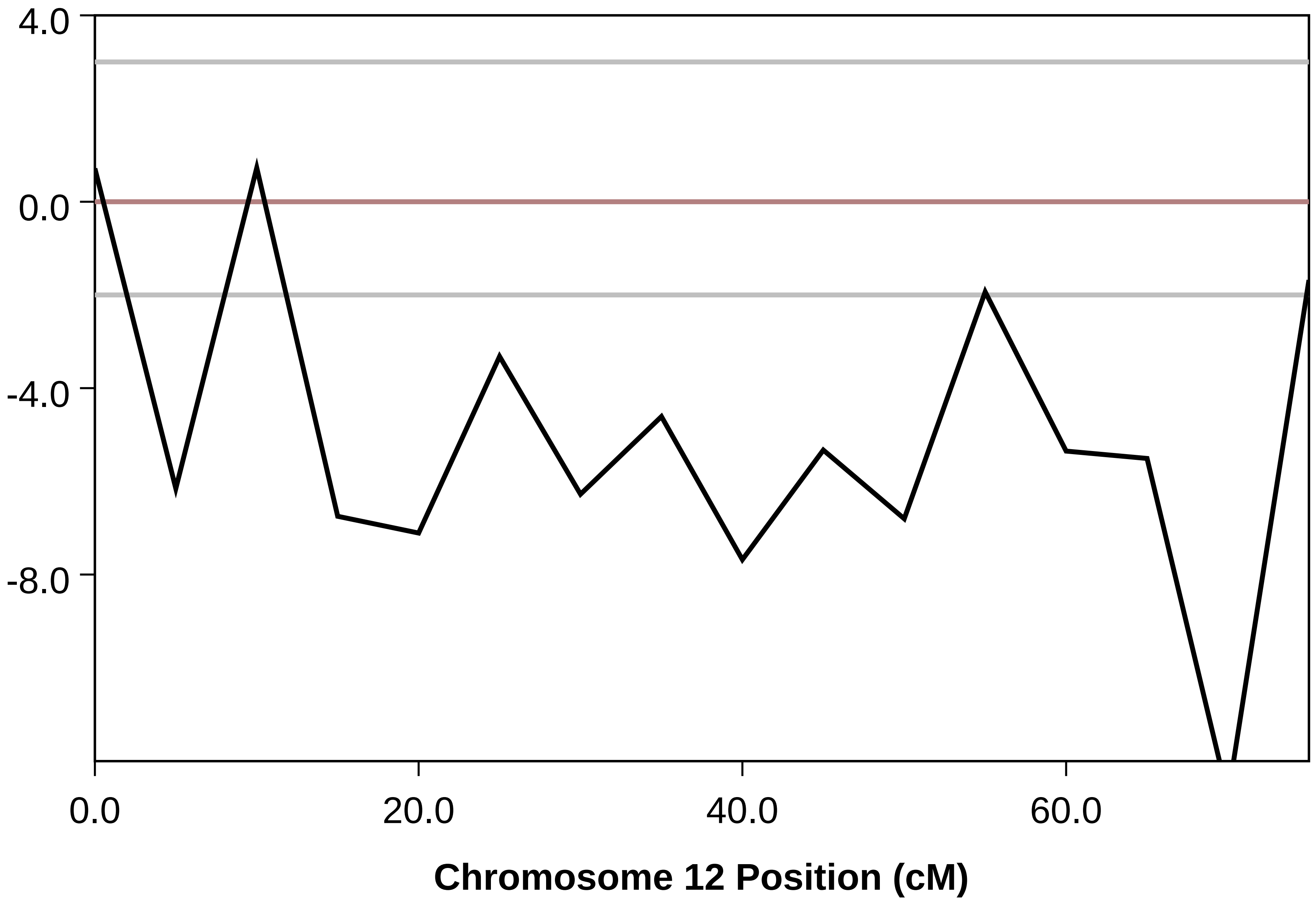

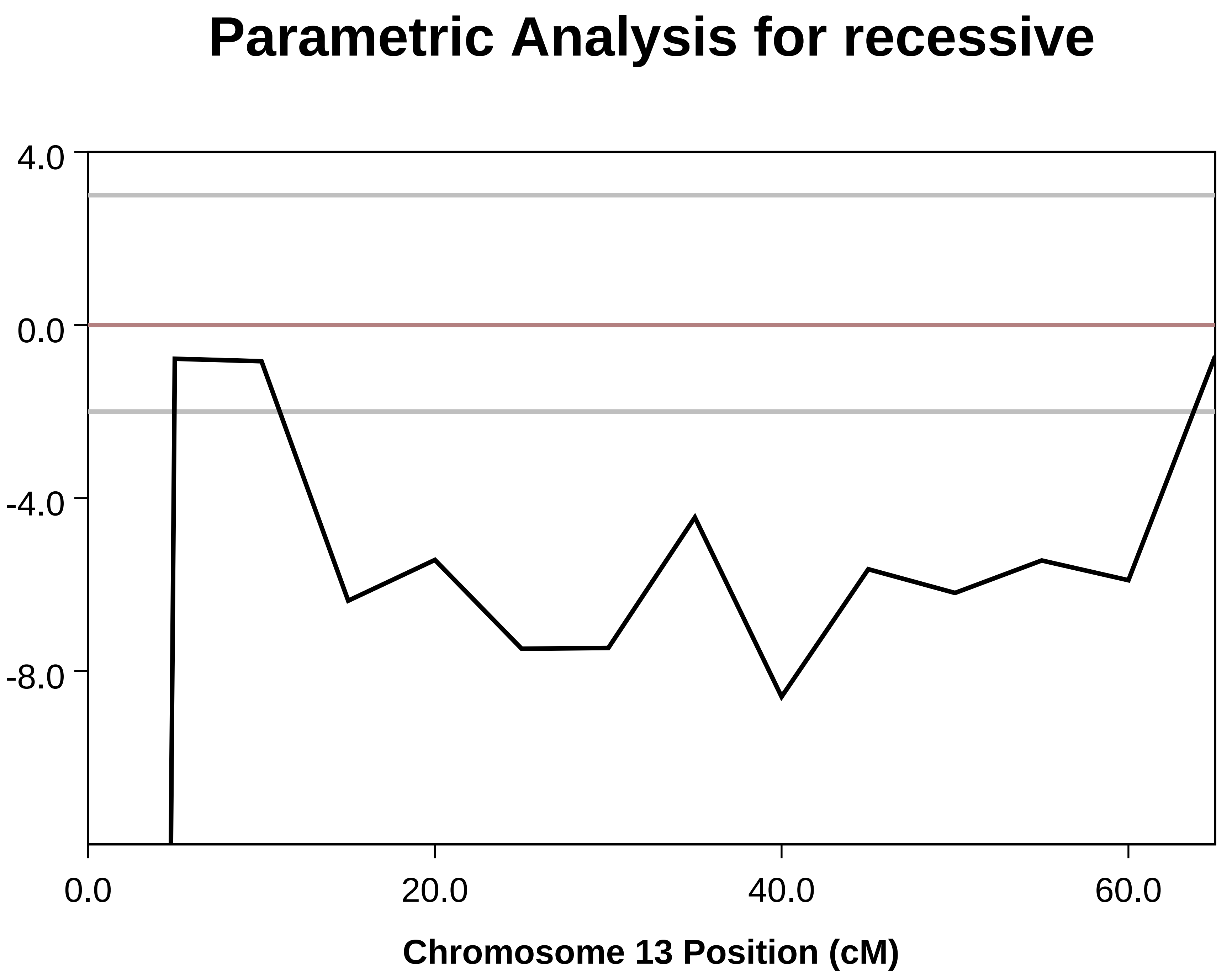
Parametric Analysis for recessive
4.0
0.0
-4.0
-8.0
0.0
20.0
40.0
60.0
Chromosome 13 Position (cM)

Parametric Analysis for recessive

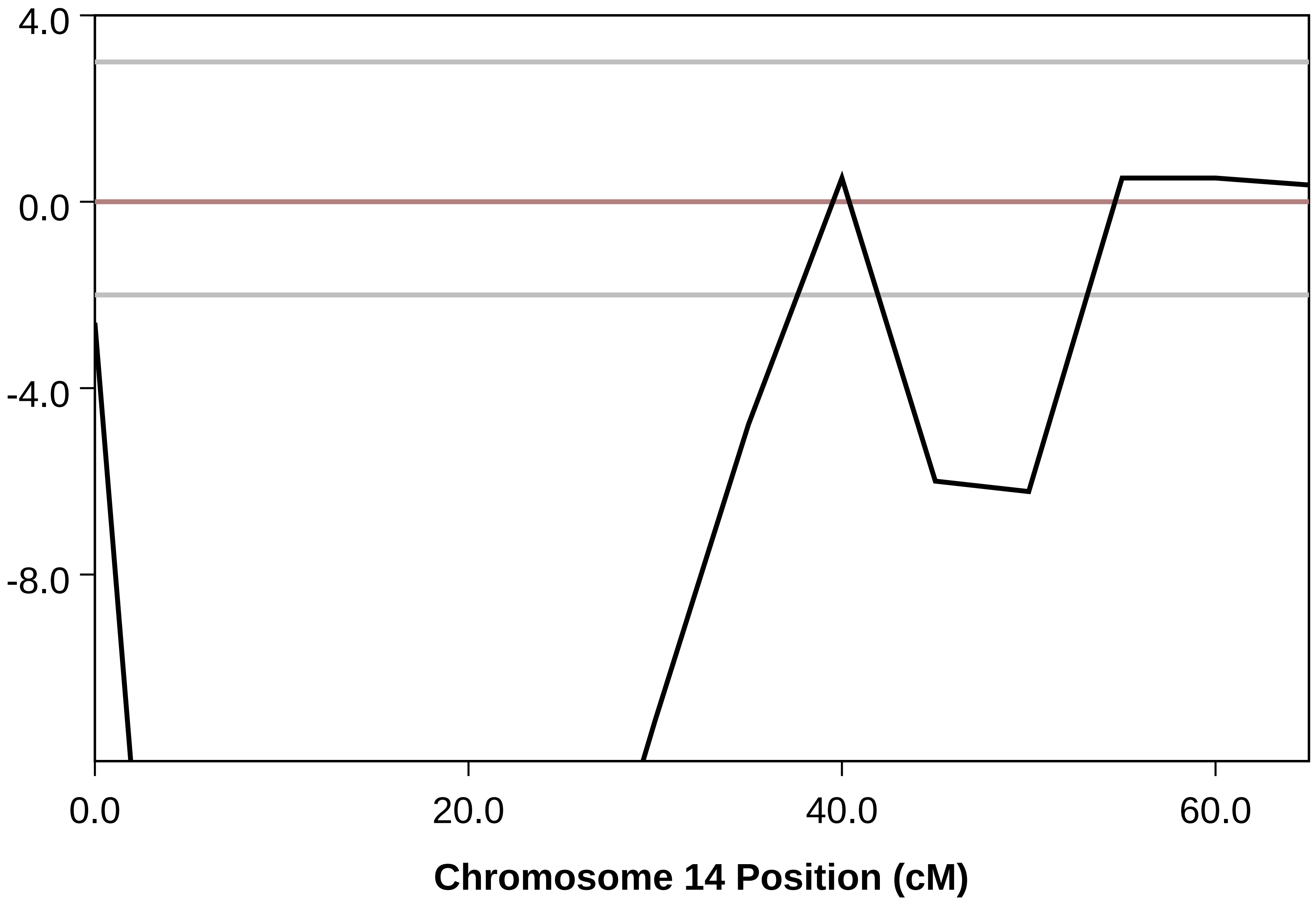

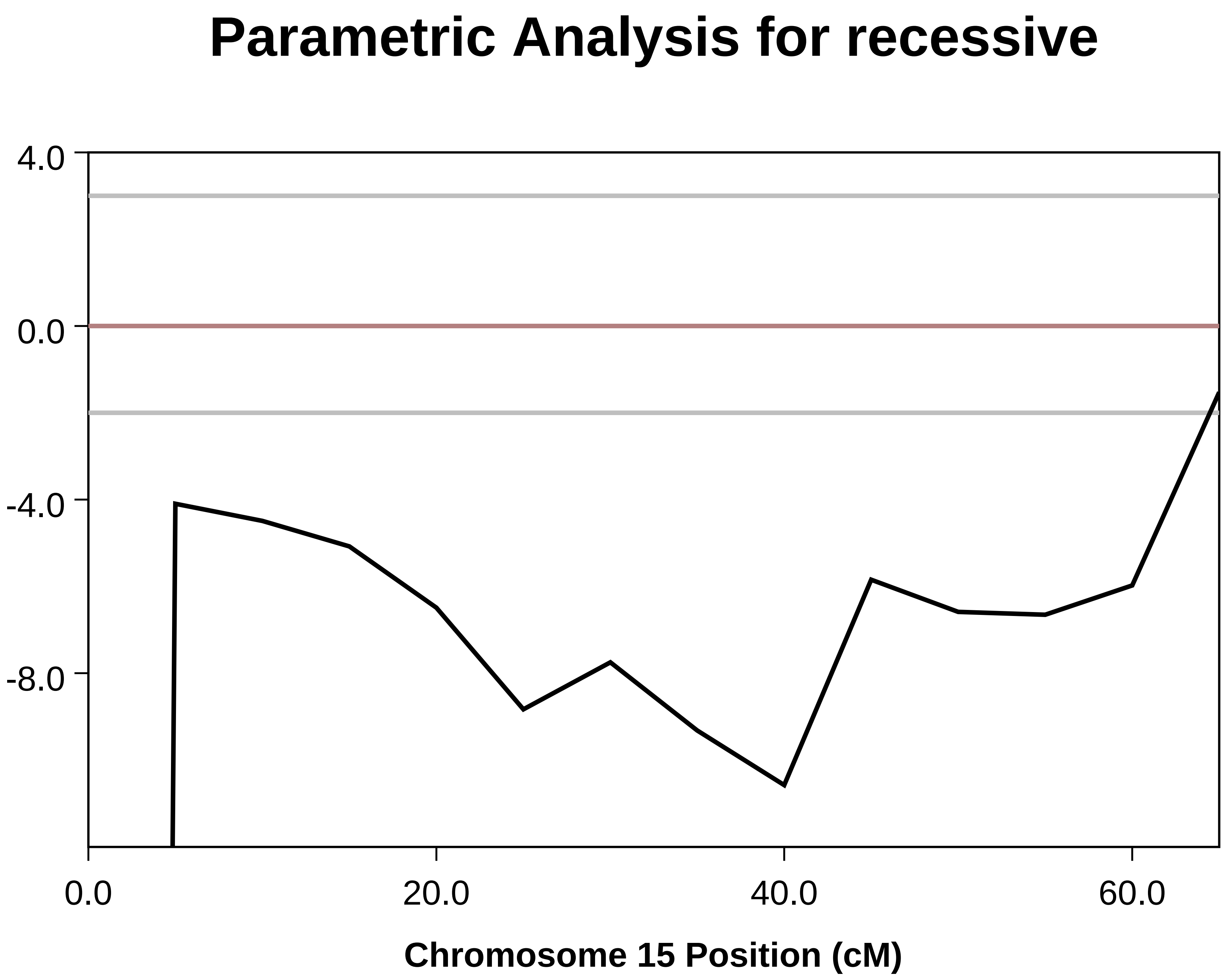
Parametric Analysis for recessive
4.0
0.0
-4.0
-8.0
0.0
20.0
40.0
60.0
Chromosome 15 Position (cM)

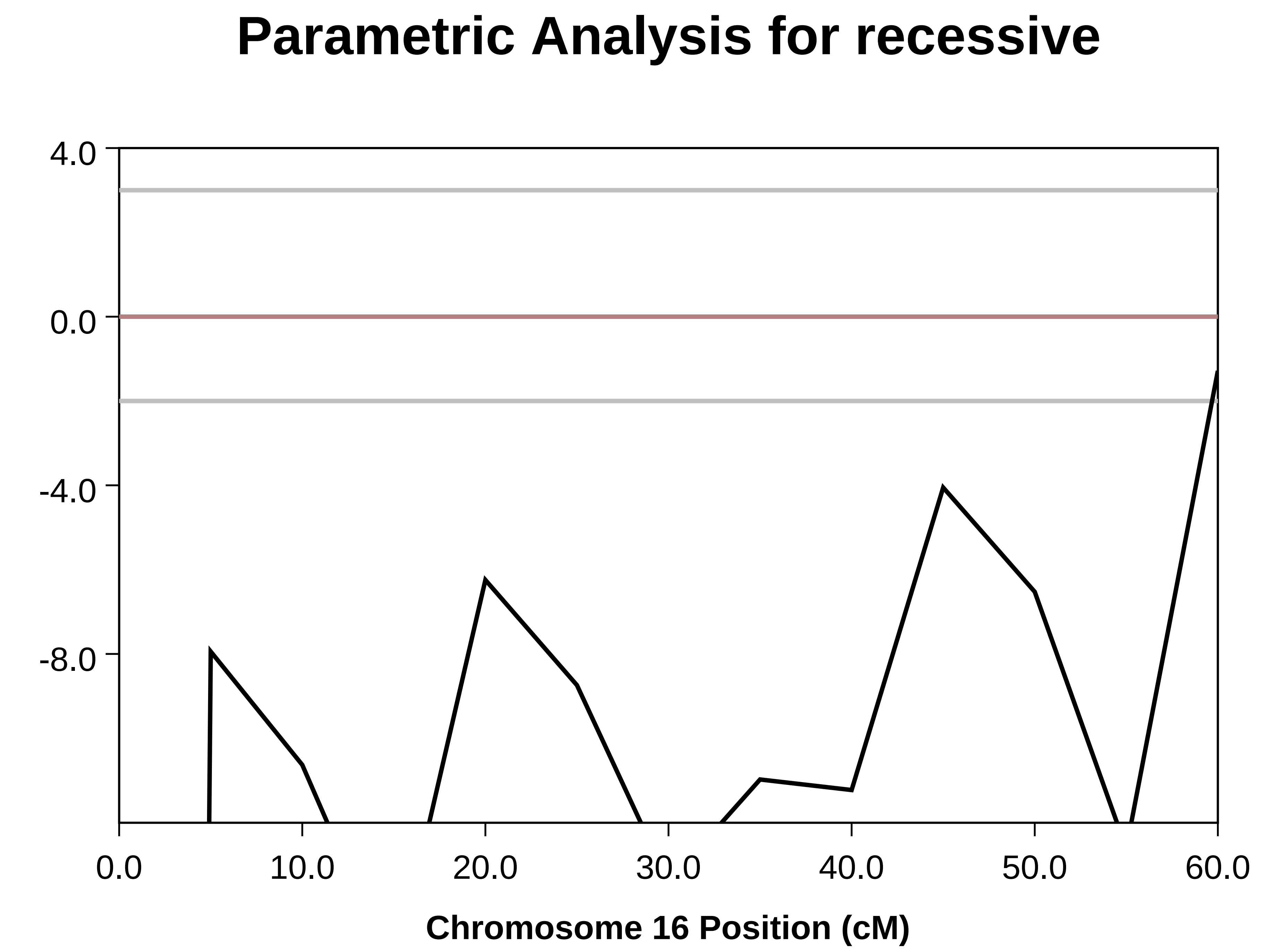
Parametric Analysis for recessive
4.0
0.0
-4.0
-8.0
0.0
10.0
20.0
30.0
40.0
50.0
60.0
Chromosome 16 Position (cM)

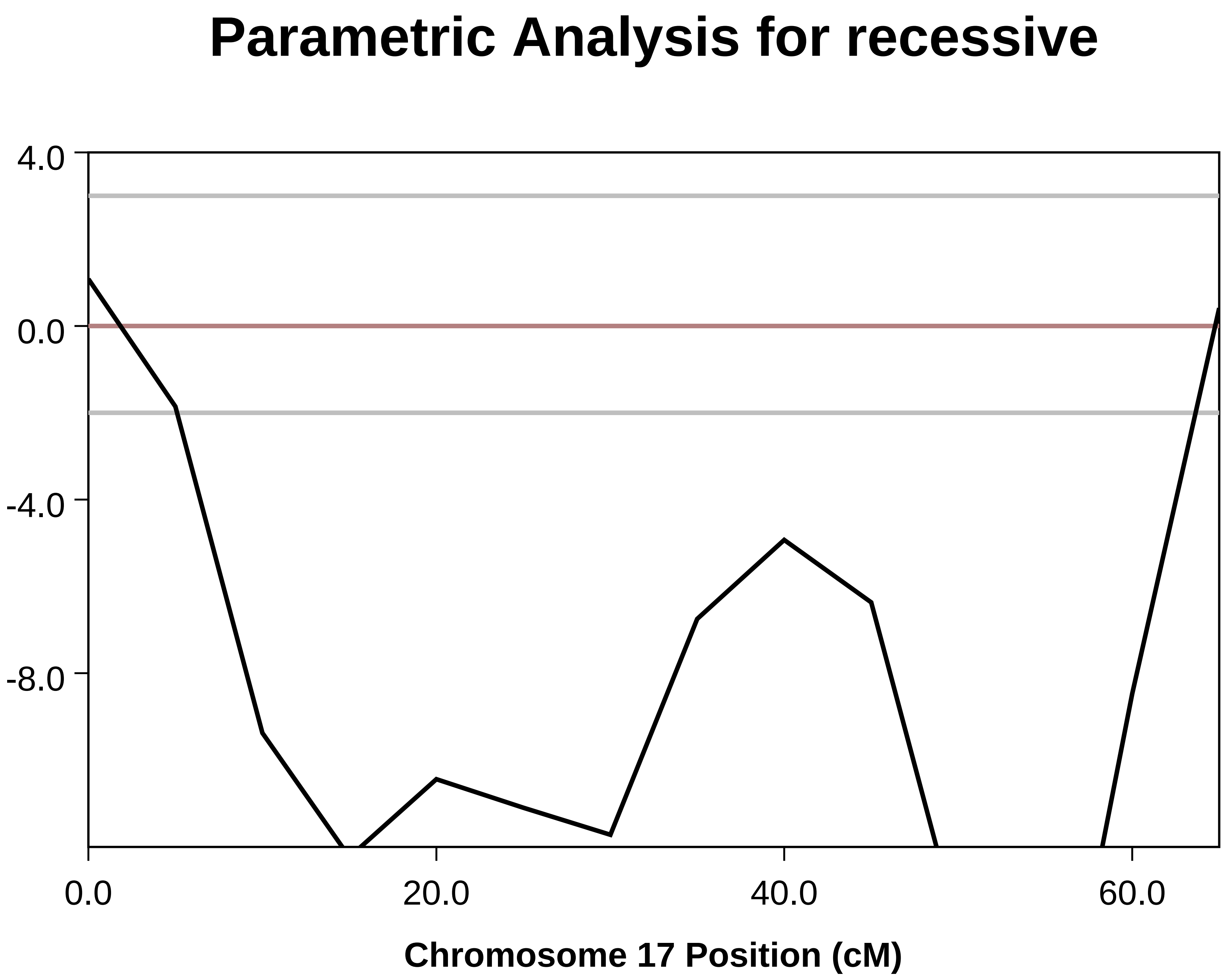
Parametric Analysis for recessive
4.0
0.0
-4.0
-8.0
0.0
20.0
40.0
60.0
Chromosome 17 Position (cM)

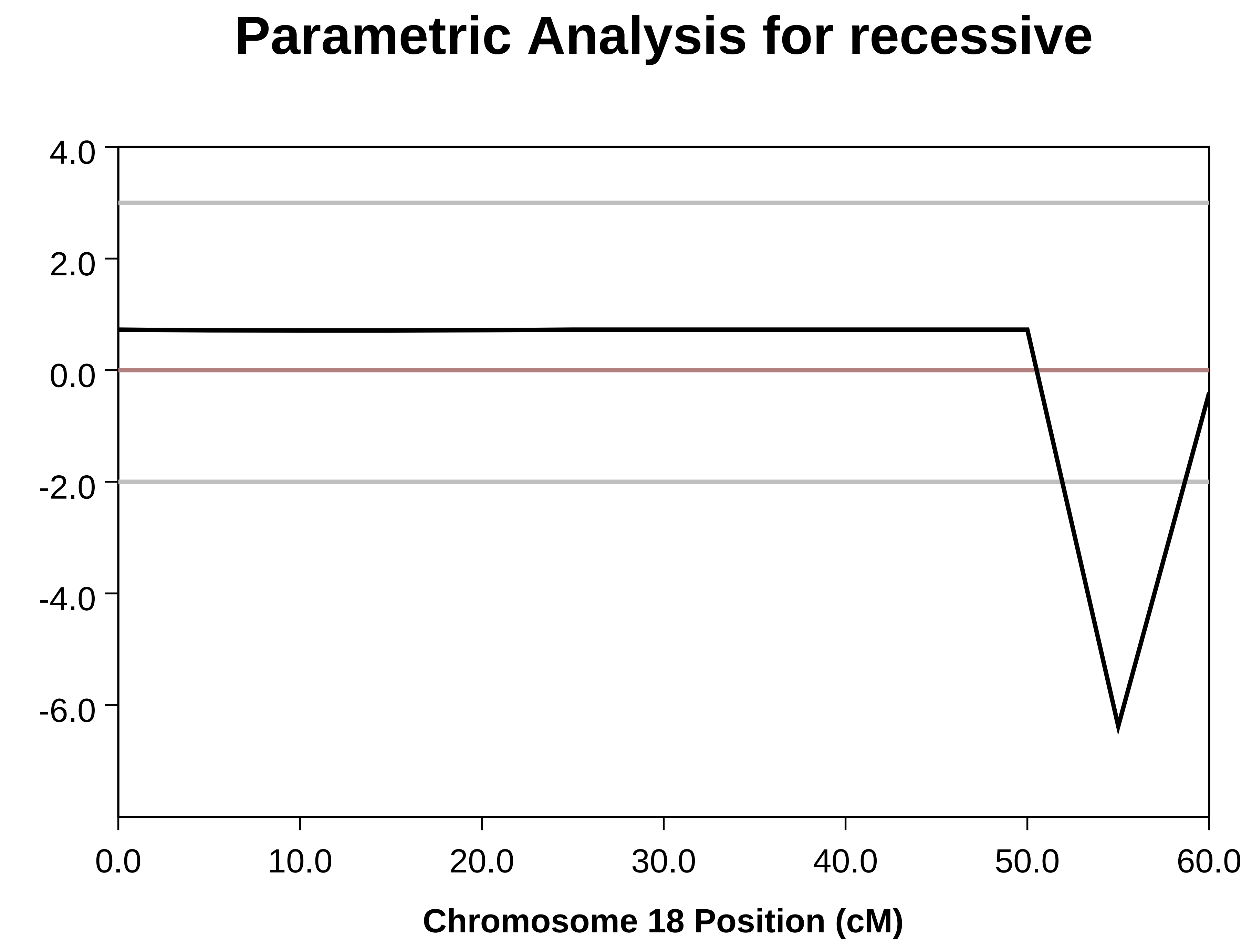
Parametric Analysis for recessive
4.0
2.0
0.0
-2.0
-4.0
-6.0
0.0
10.0
20.0
30.0
40.0
50.0
60.0
Chromosome 18 Position (cM)

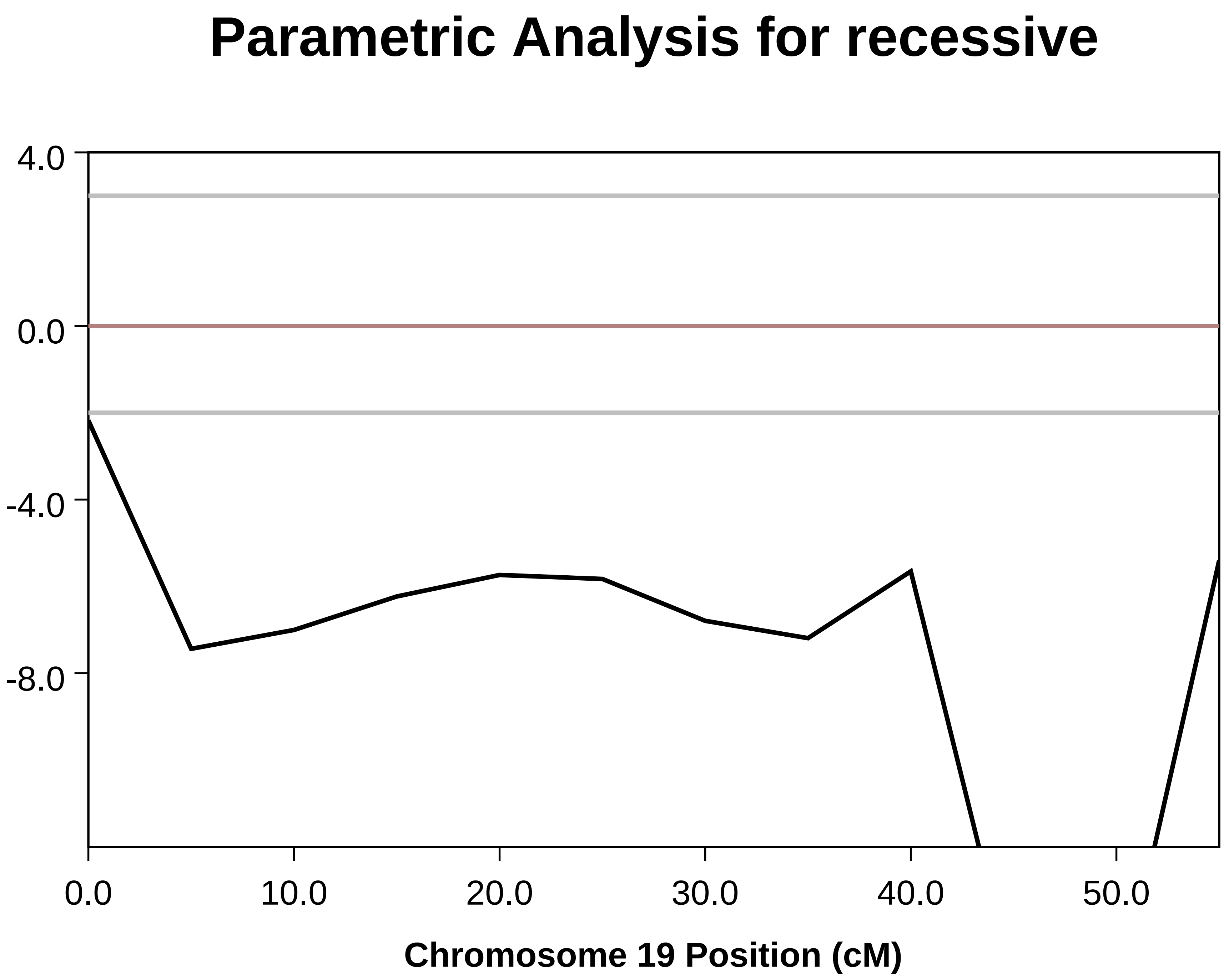
Parametric Analysis for recessive
4.0
0.0
-4.0
-8.0
0.0
10.0
20.0
30.0
40.0
50.0
Chromosome 19 Position (cM)

Parametric Analysis for recessive

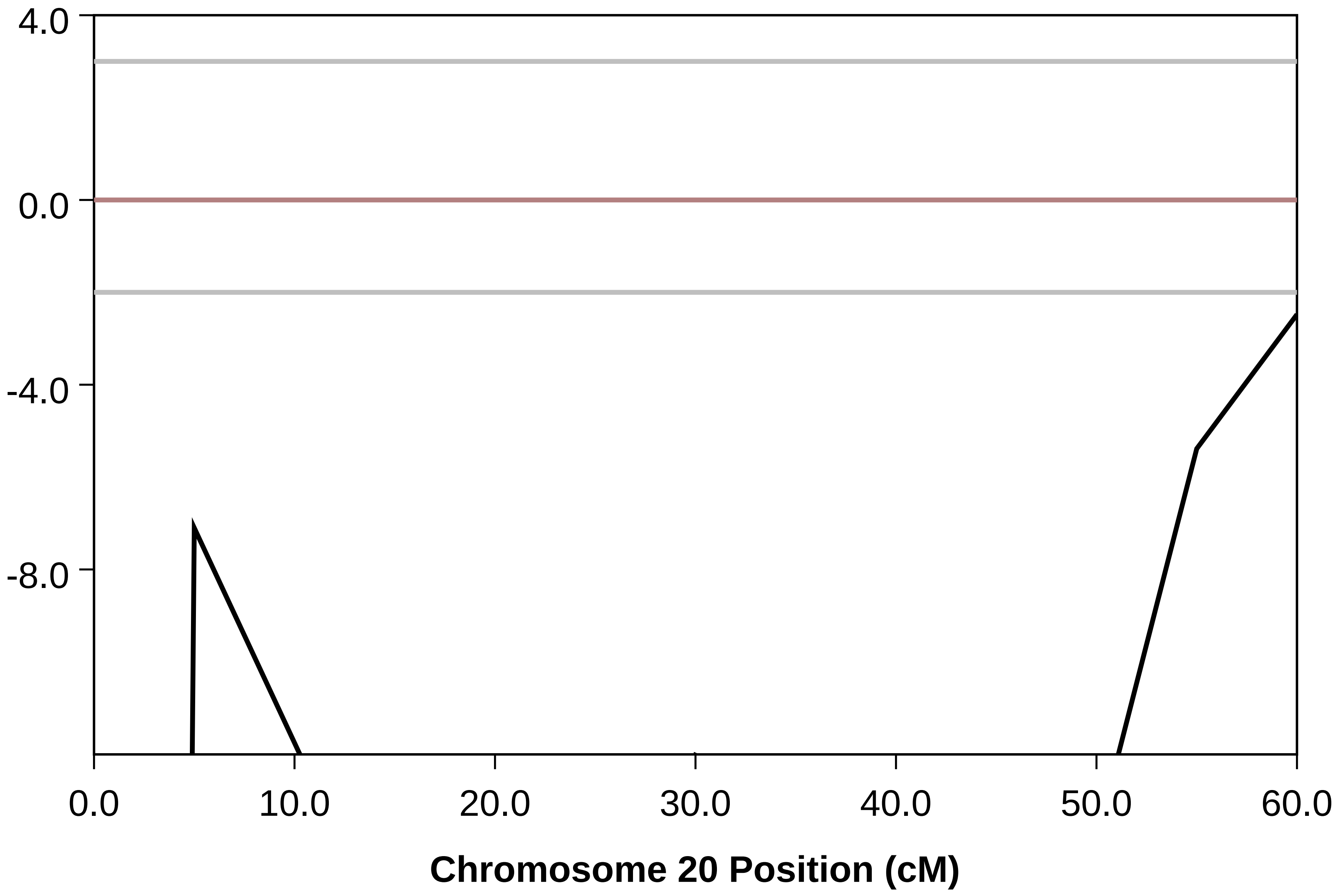

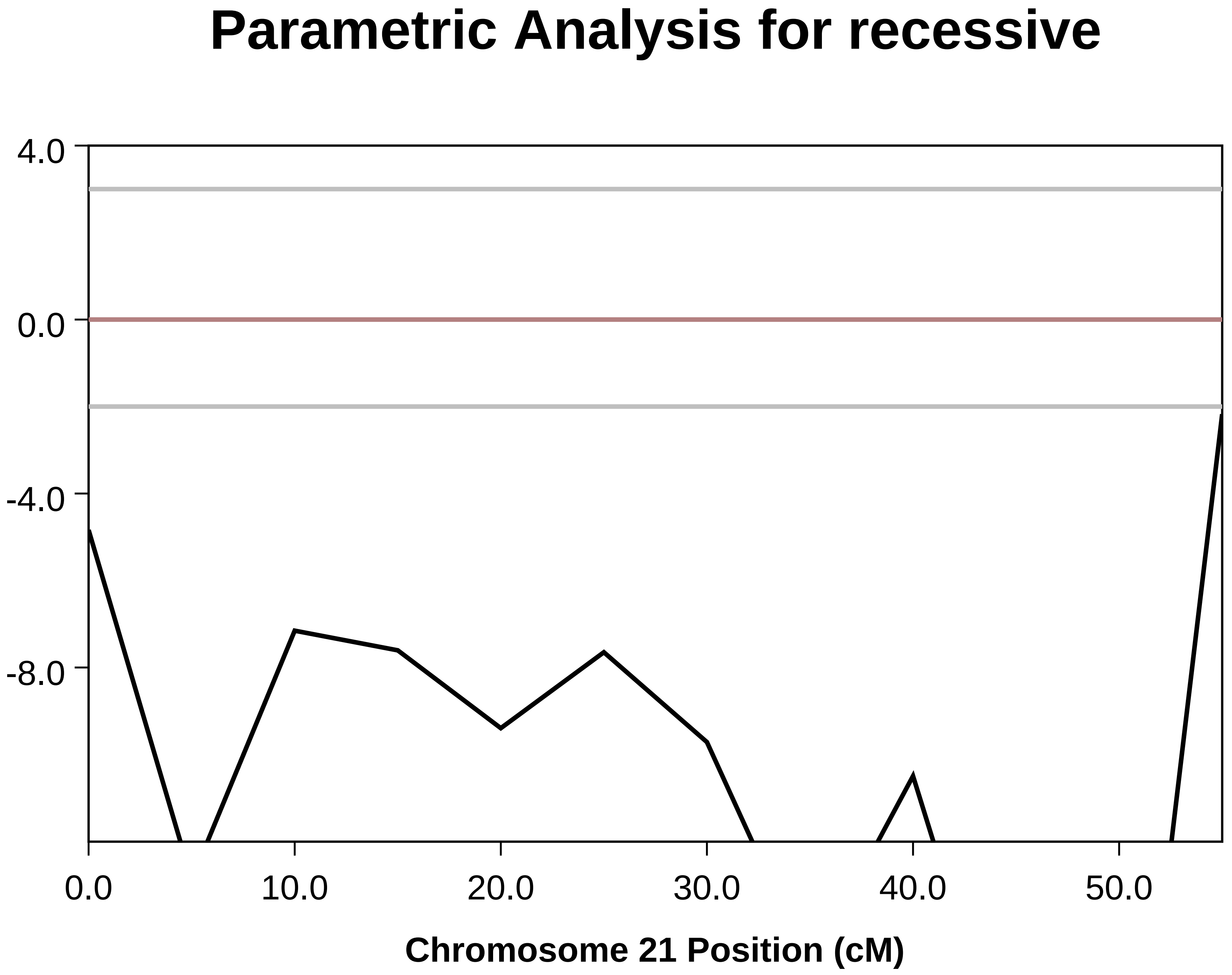
Parametric Analysis for recessive
4.0
0.0
-4.0
-8.0
0.0
10.0
20.0
30.0
40.0
50.0
Chromosome 21 Position (cM)

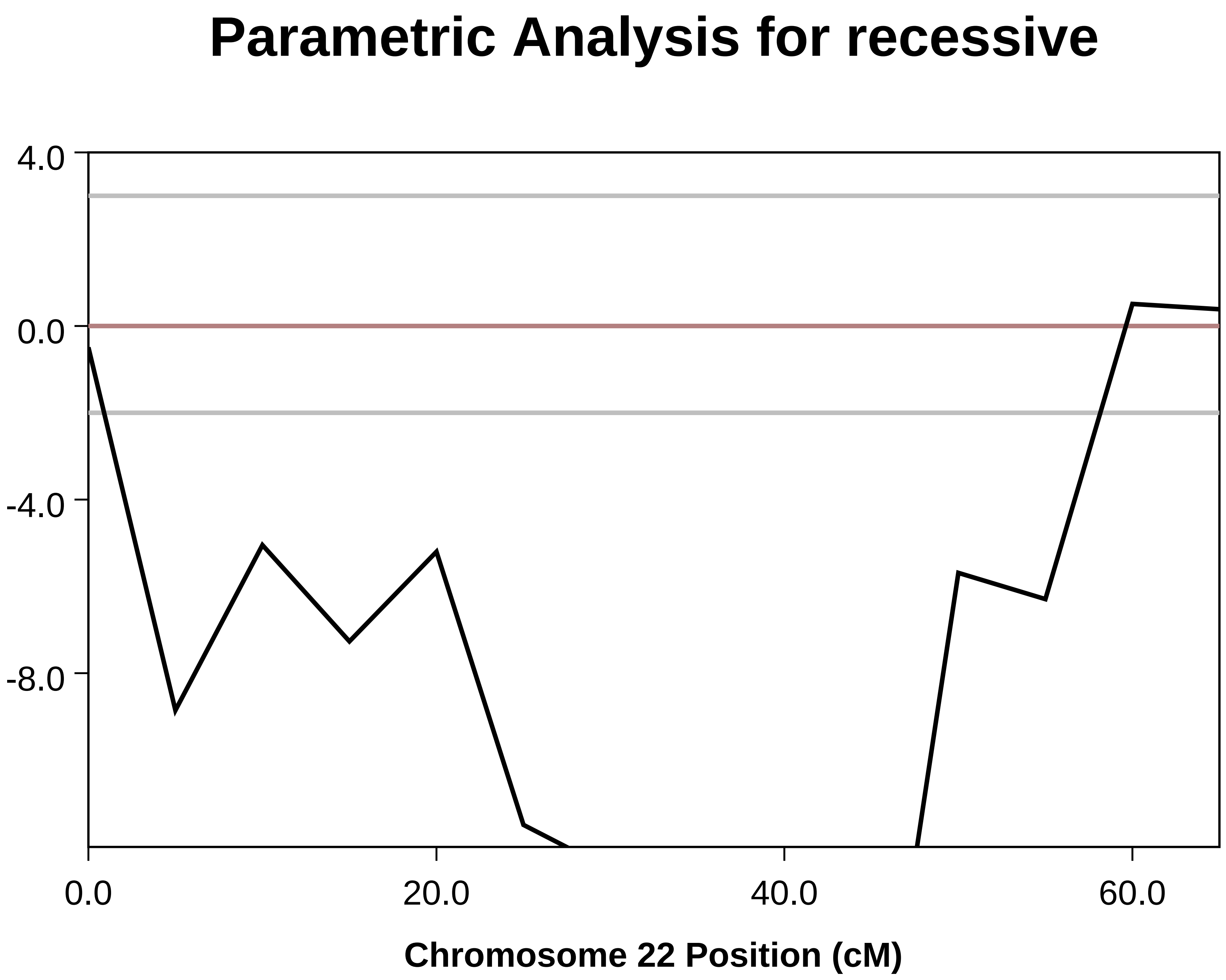
Parametric Analysis for recessive
4.0
0.0
-4.0
-8.0
0.0
20.0
40.0
60.0
Chromosome 22 Position (cM)

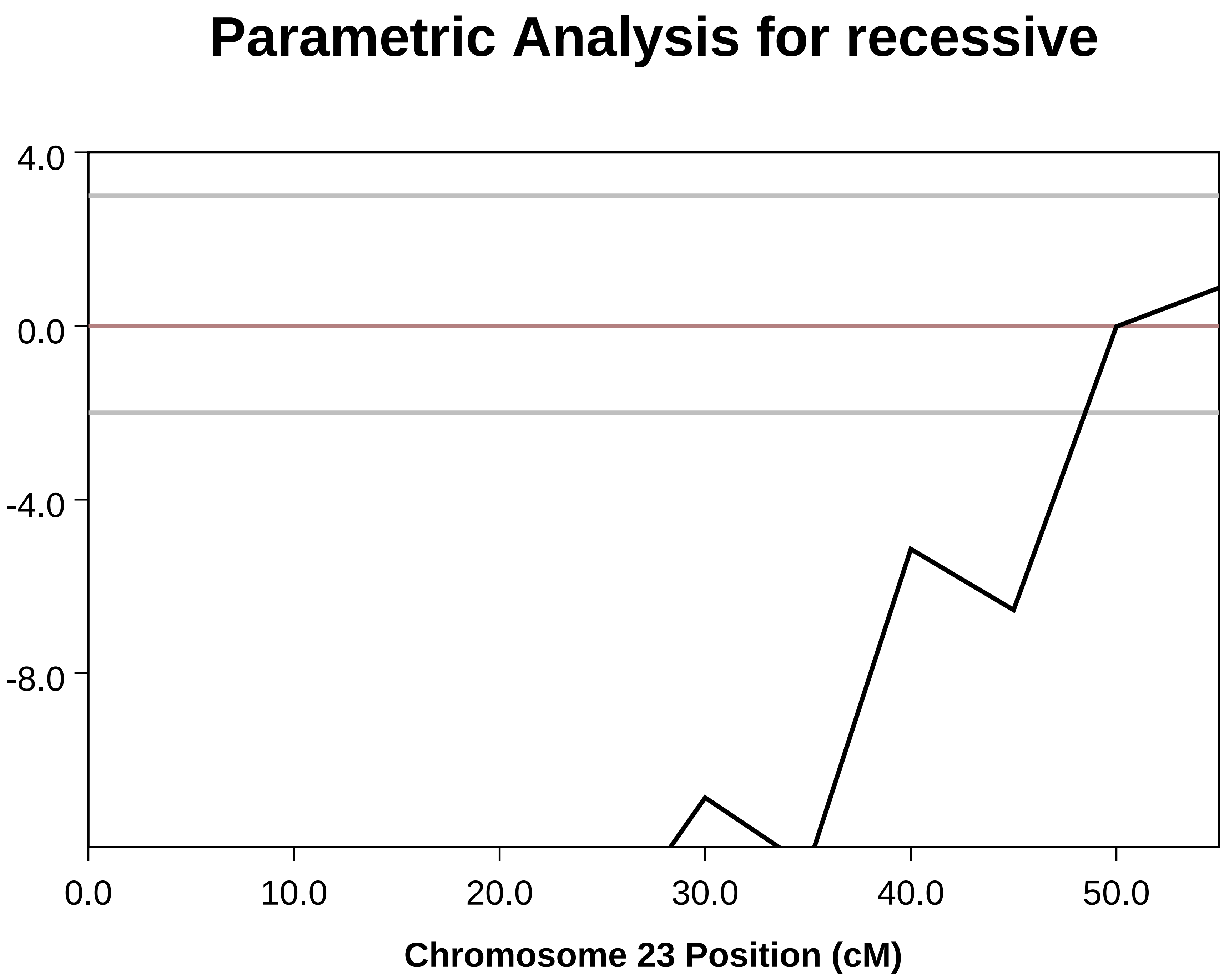
Parametric Analysis for recessive
4.0
0.0
-4.0
-8.0
0.0
10.0
20.0
30.0
40.0
50.0
Chromosome 23 Position (cM)

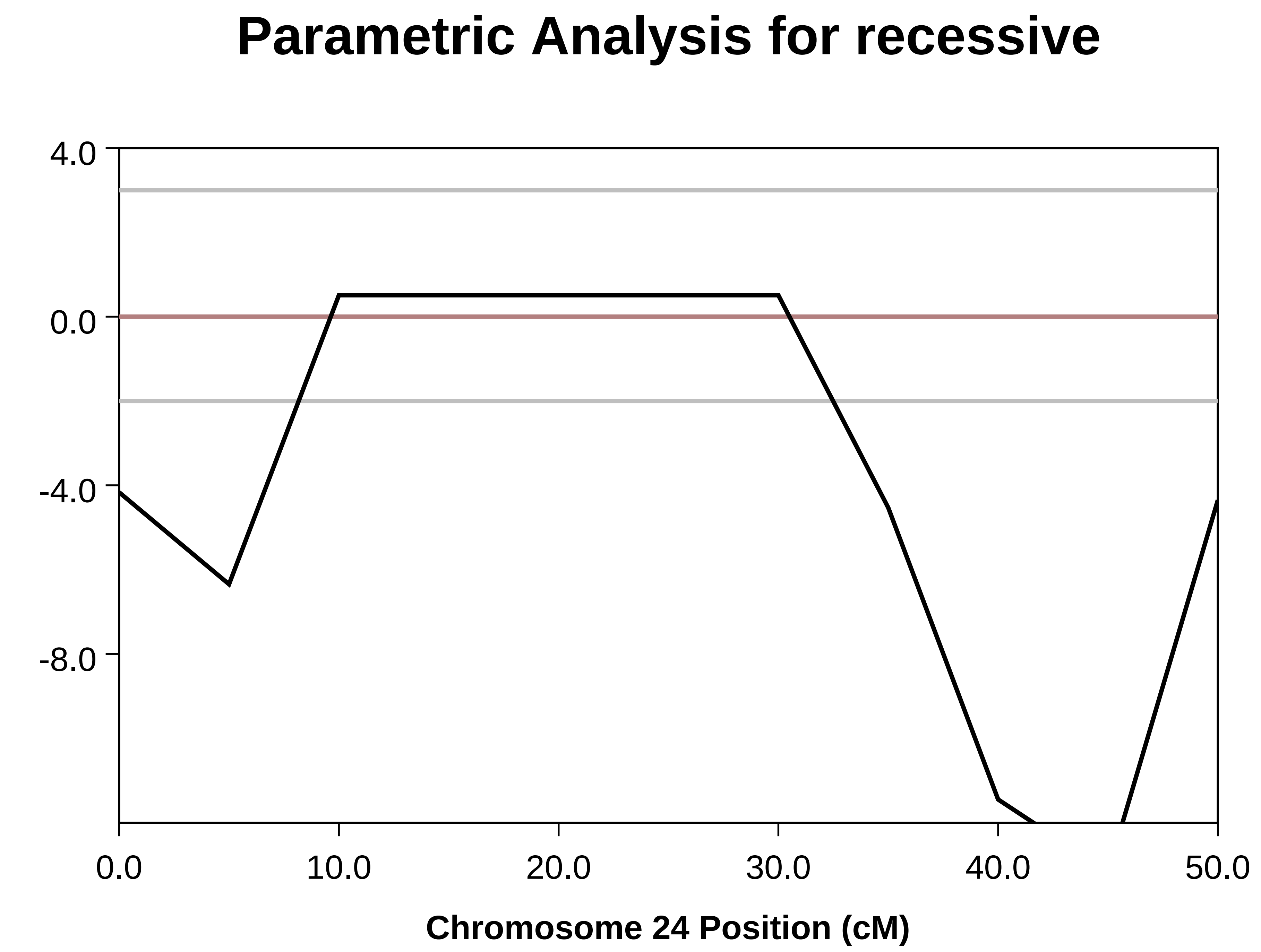

Parametric Analysis for recessive
4.0
0.0
-4.0
-8.0
0.0
10.0
20.0
30.0
40.0
50.0
Chromosome 24 Position (cM)

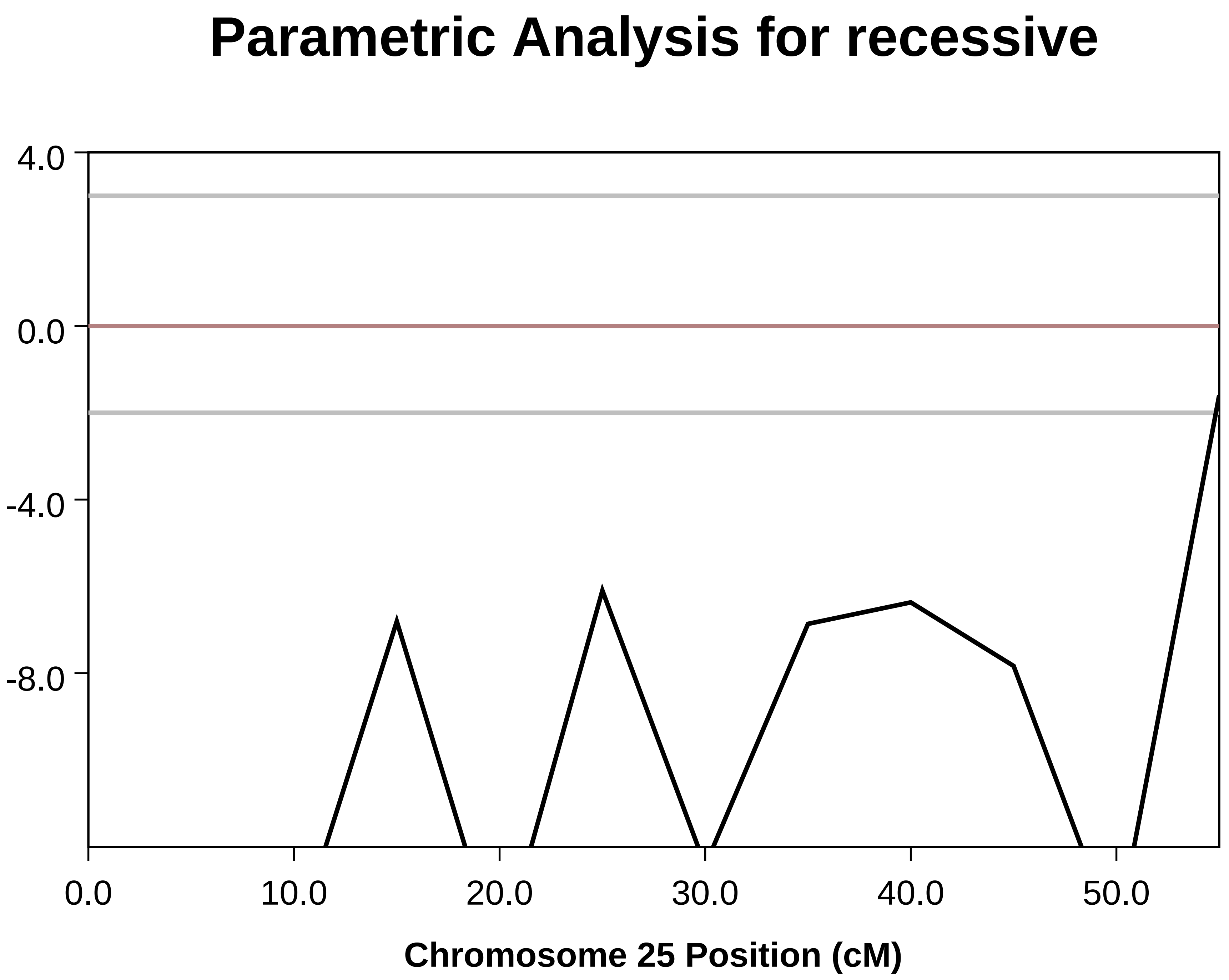

Parametric Analysis for recessive
4.0
0.0
-4.0
-8.0
0.0
10.0
20.0
30.0
40.0
50.0
Chromosome 25 Position (cM)

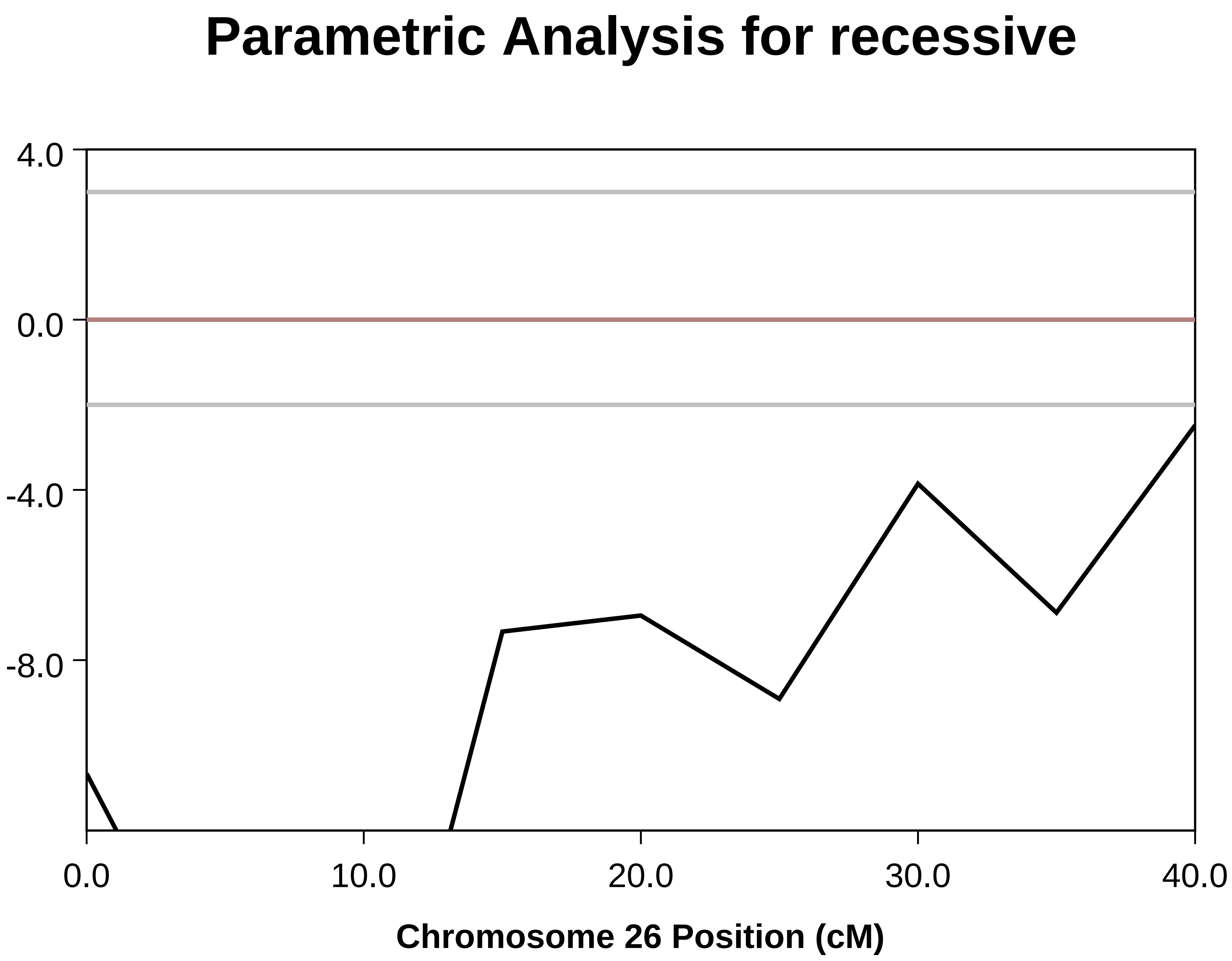

Parametric Analysis for recessive
4.0
0.0
-4.0
-8.0
0.0
10.0
20.0
30.0
40.0
Chromosome 26 Position (cM)

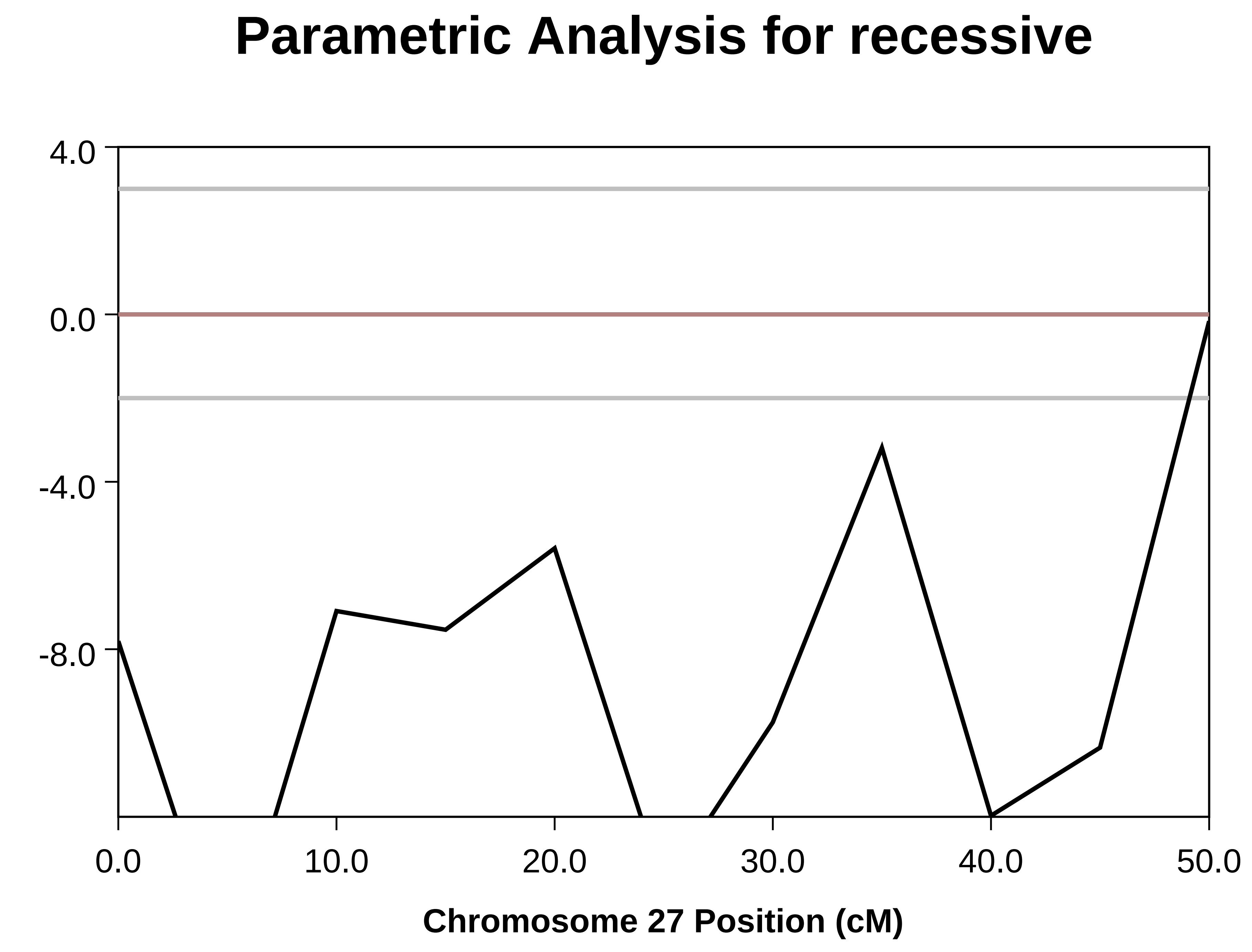
Parametric Analysis for recessive
4.0
0.0
-4.0
-8.0
0.0
10.0
20.0
30.0
40.0
50.0
Chromosome 27 Position (cM)

Parametric Analysis for recessive

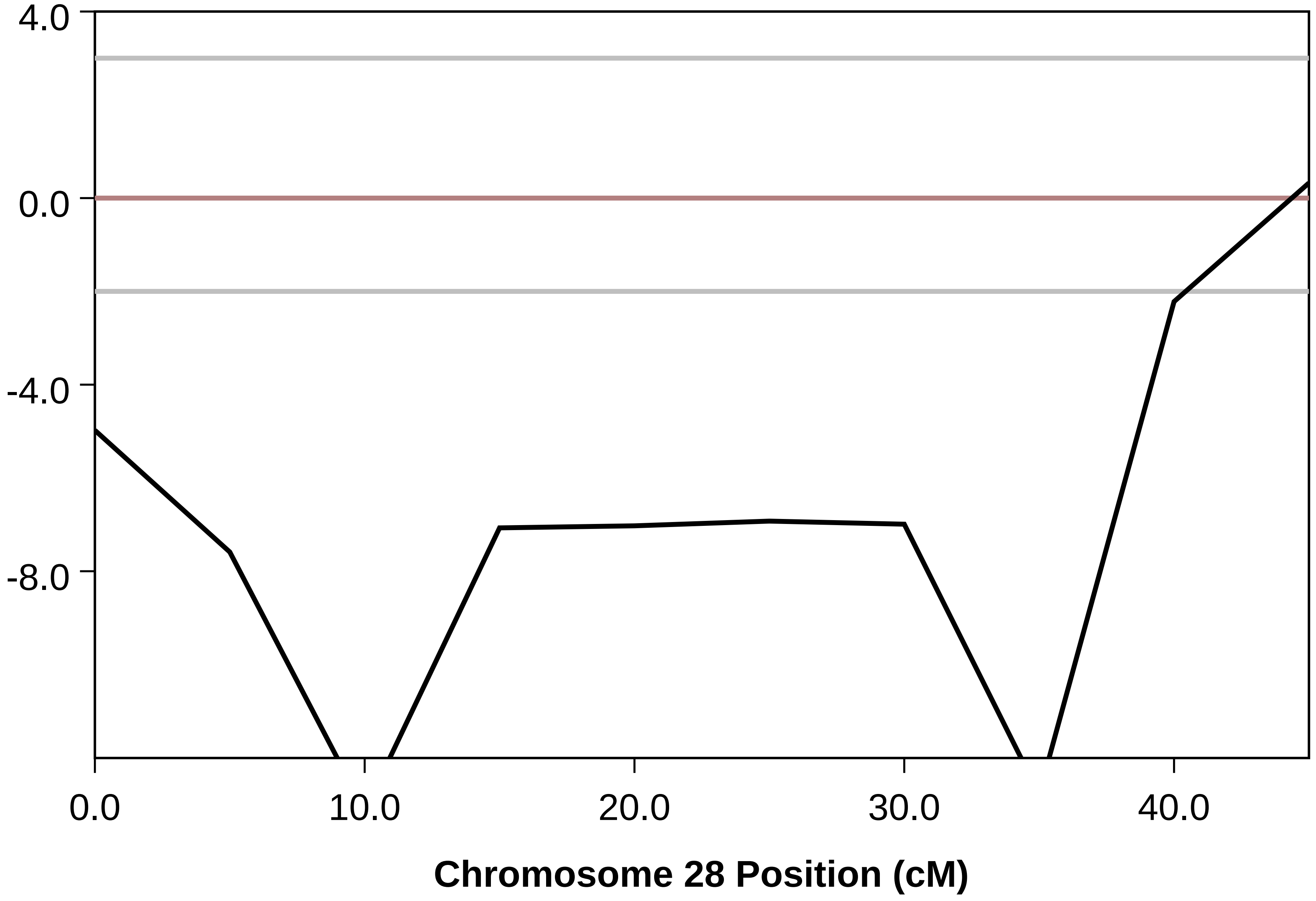

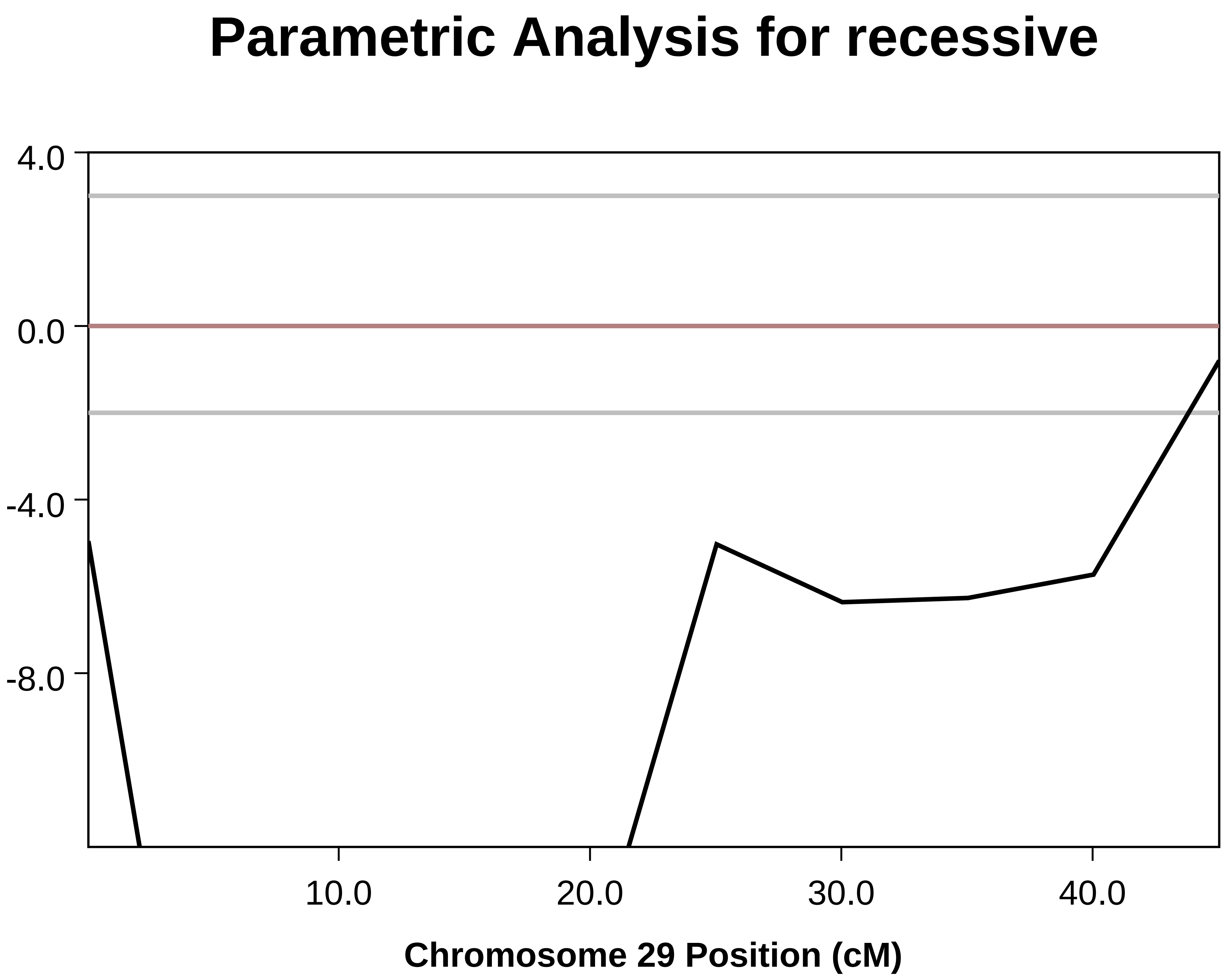
Parametric Analysis for recessive
4.0
0.0
-4.0
-8.0
10.0
20.0
30.0
40.0
Chromosome 29 Position (cM)

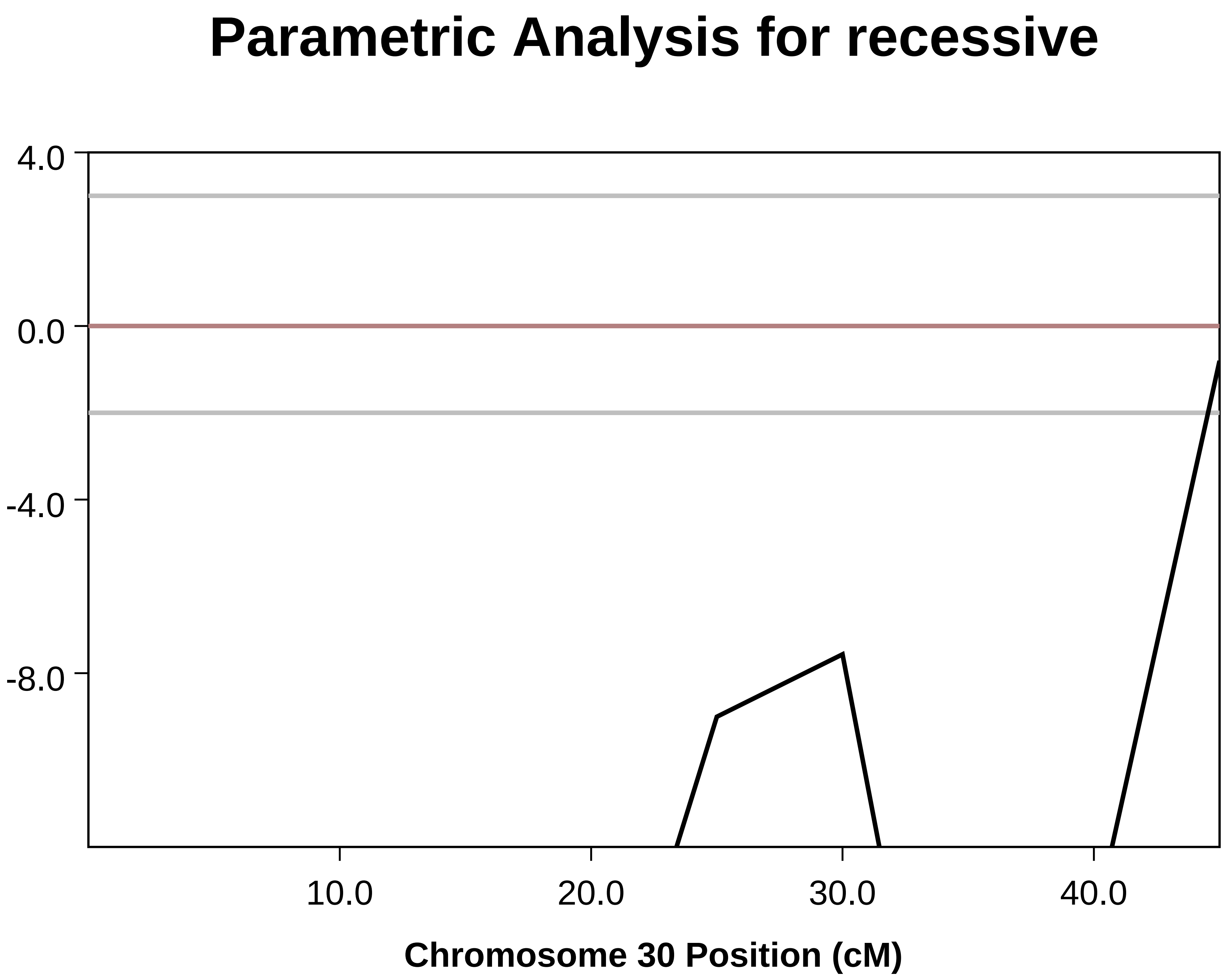
Parametric Analysis for recessive
4.0
0.0
-4.0
-8.0
10.0
20.0
30.0
40.0
Chromosome 30 Position (cM)

Parametric Analysis for recessive

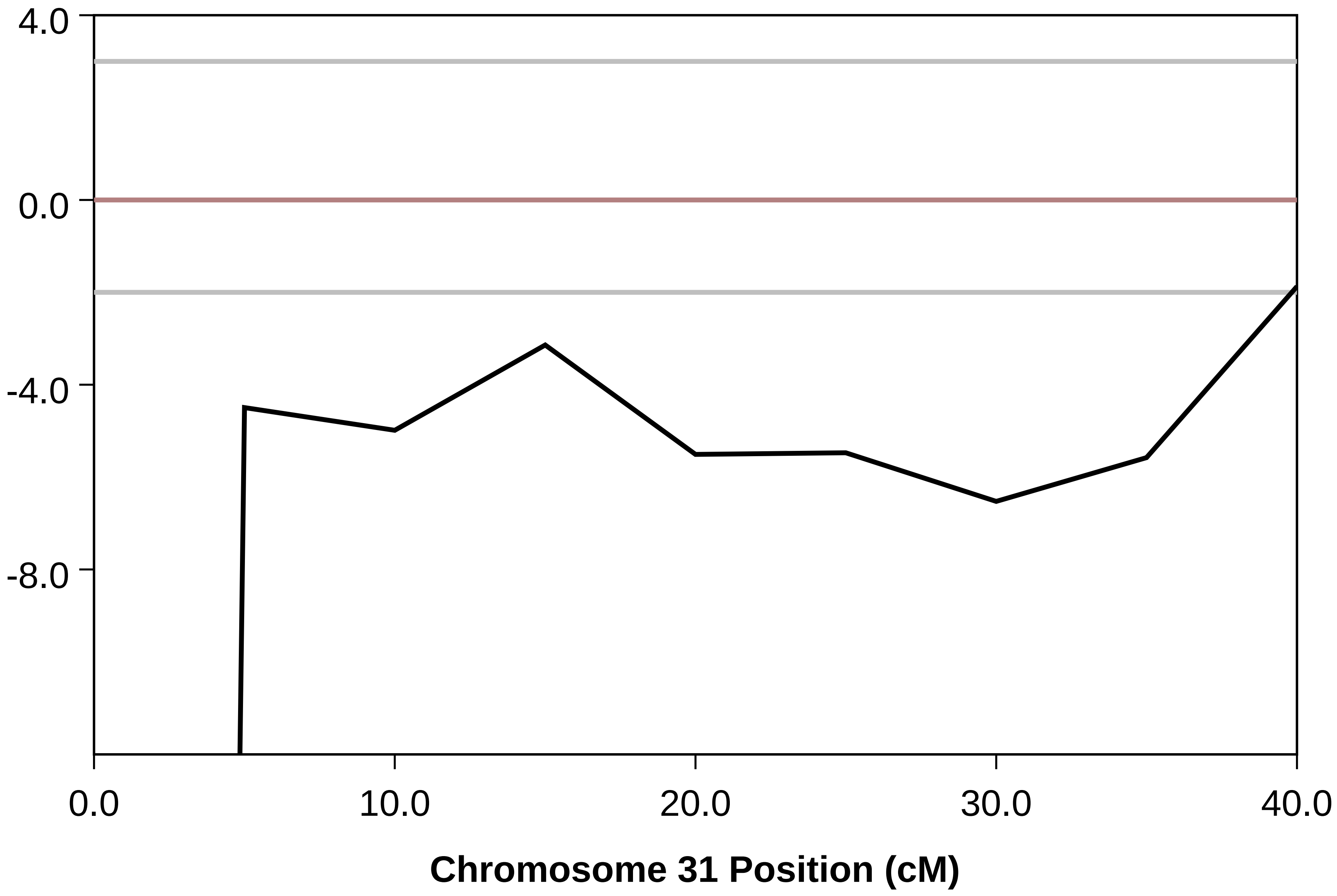

Parametric Analysis for recessive

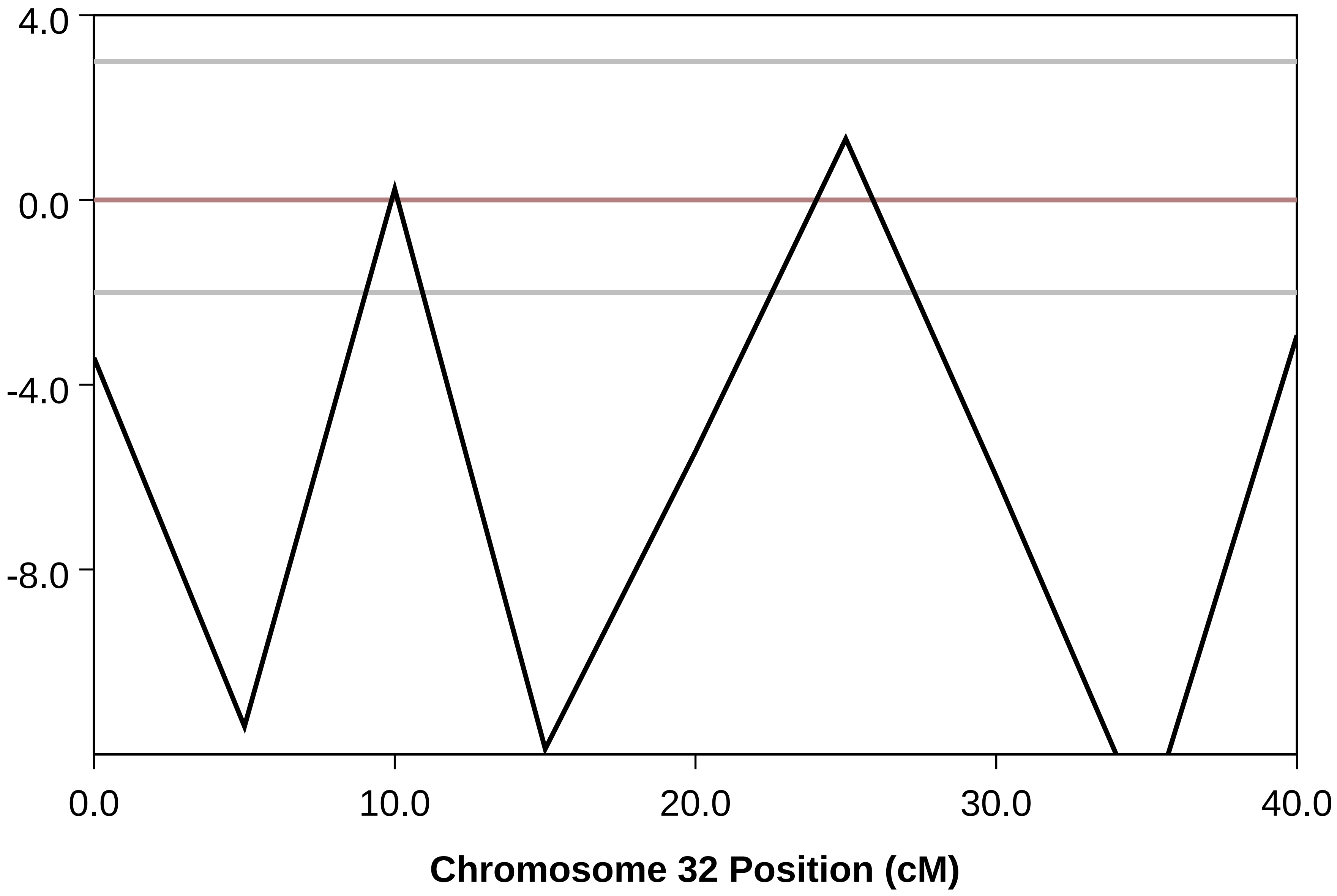

Parametric Analysis for recessive

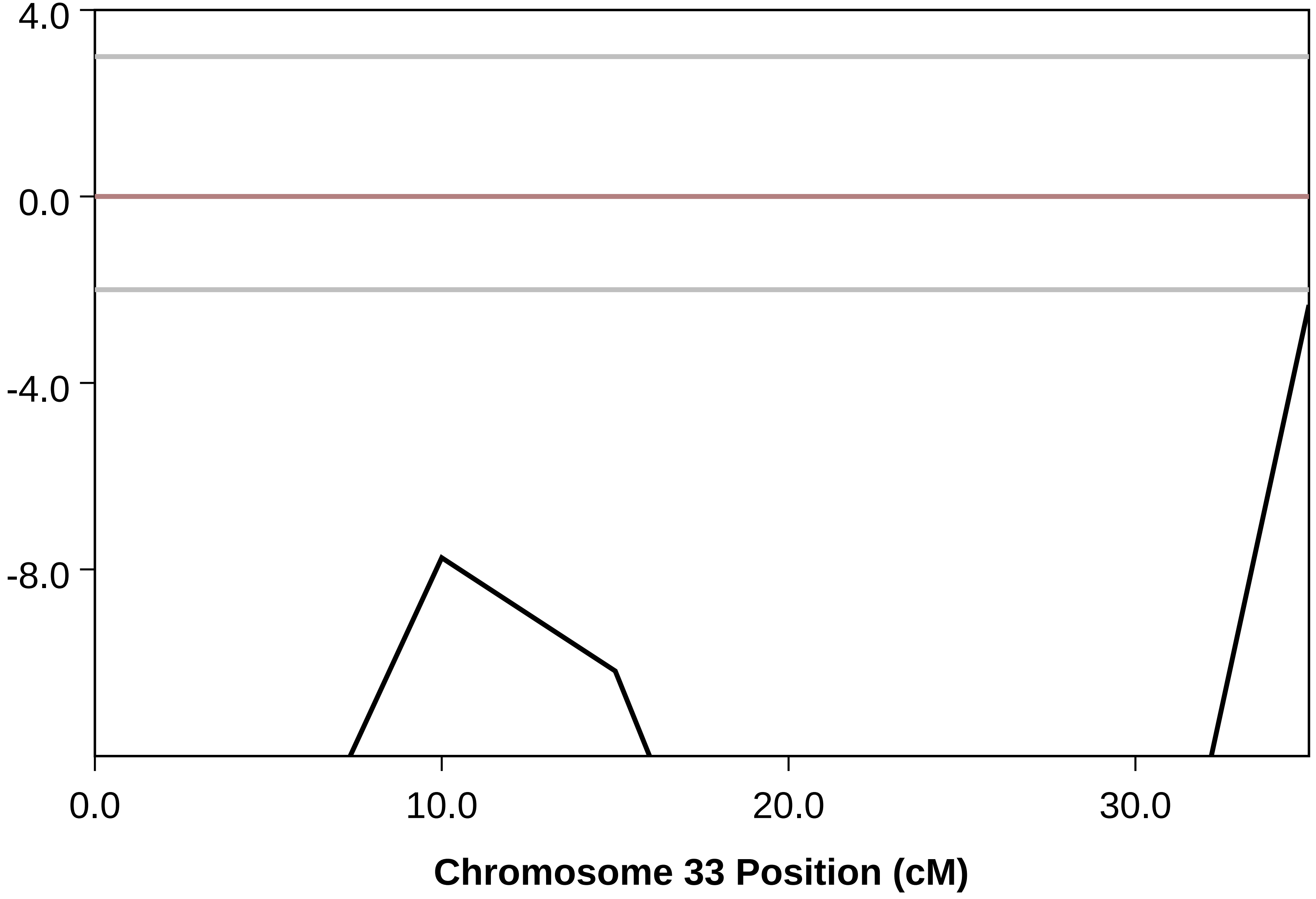

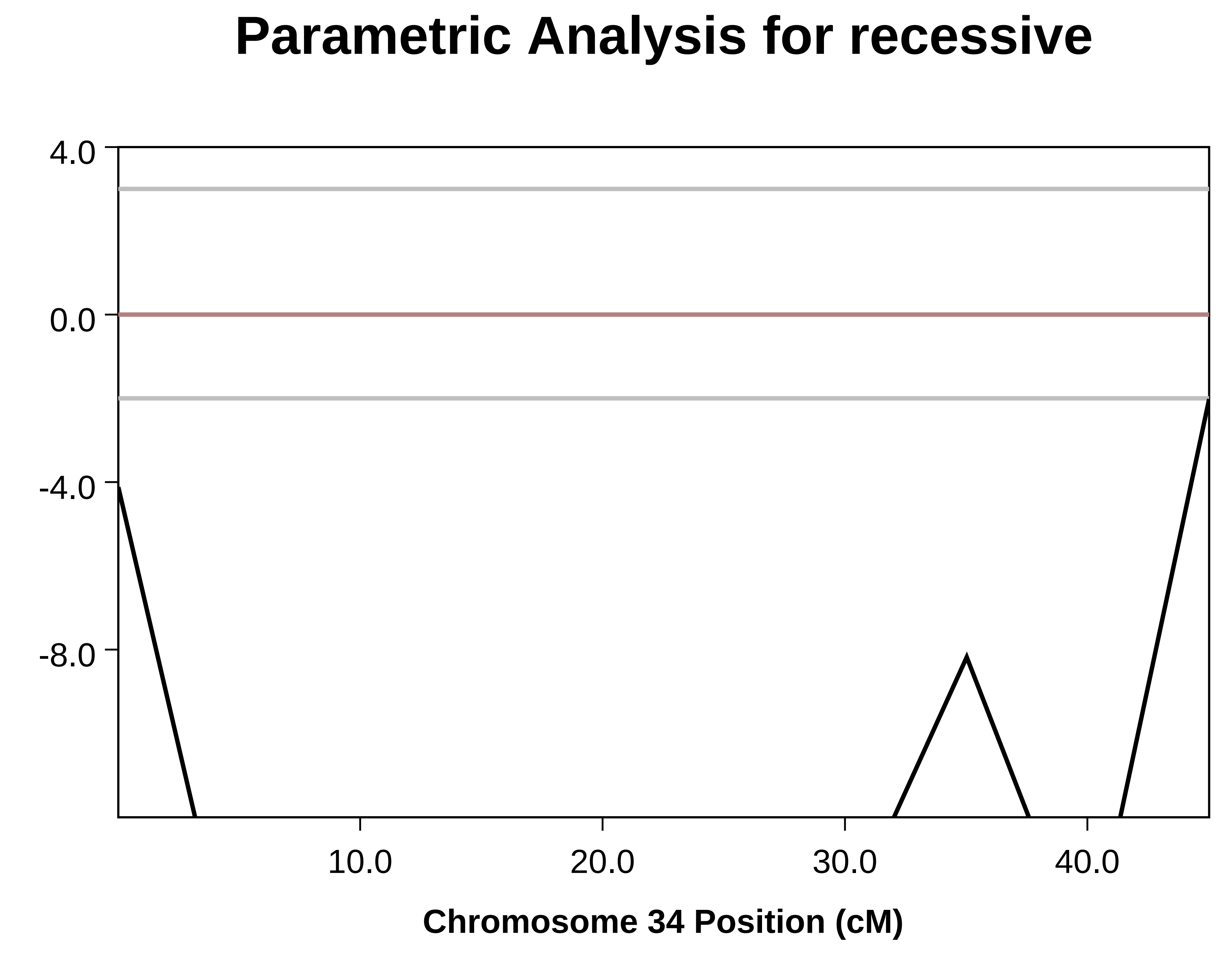
Parametric Analysis for recessive
4.0
0.0
-4.0
-8.0
10.0
20.0
30.0
40.0
Chromosome 34 Position (cM)

Parametric Analysis for recessive

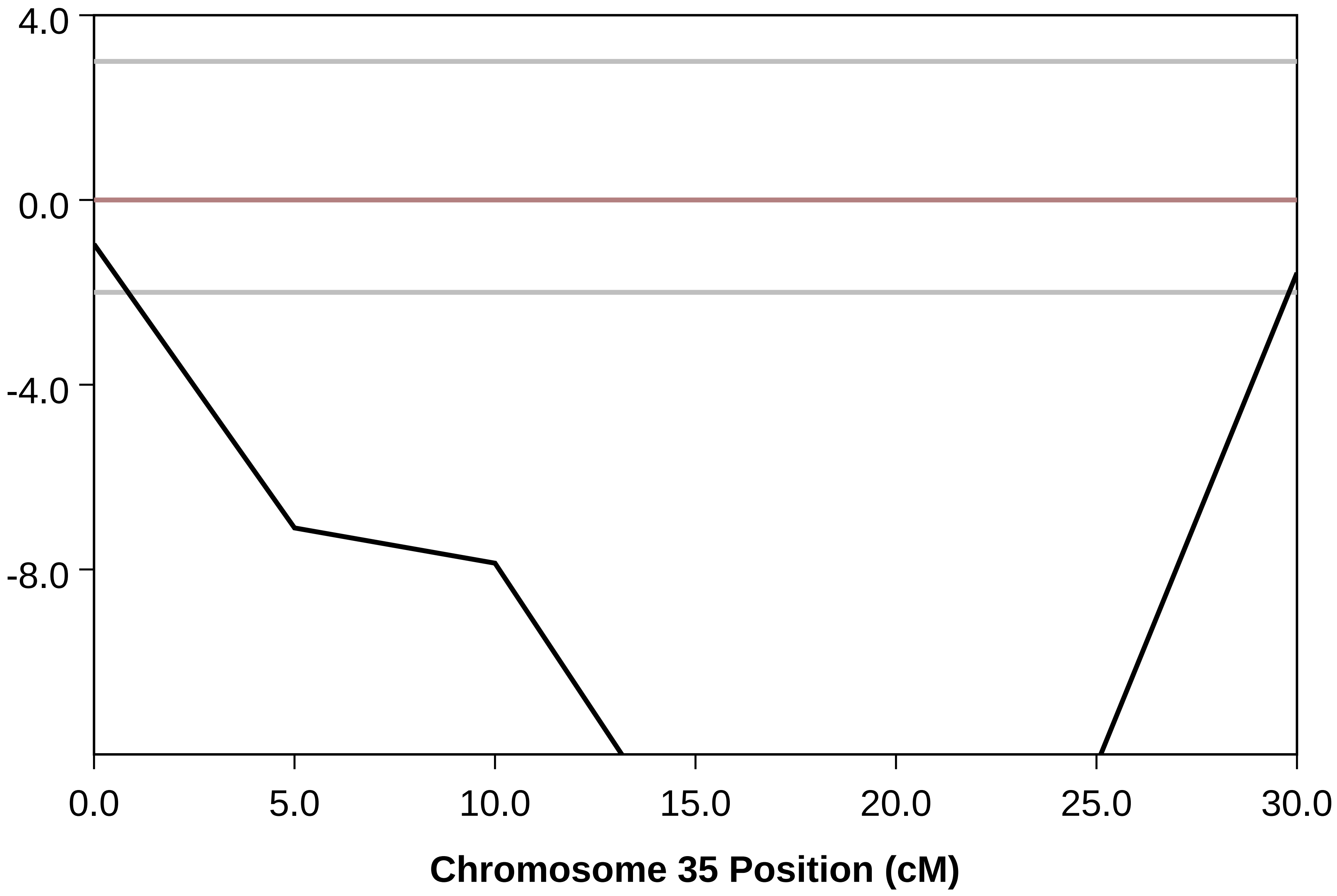

Parametric Analysis for recessive

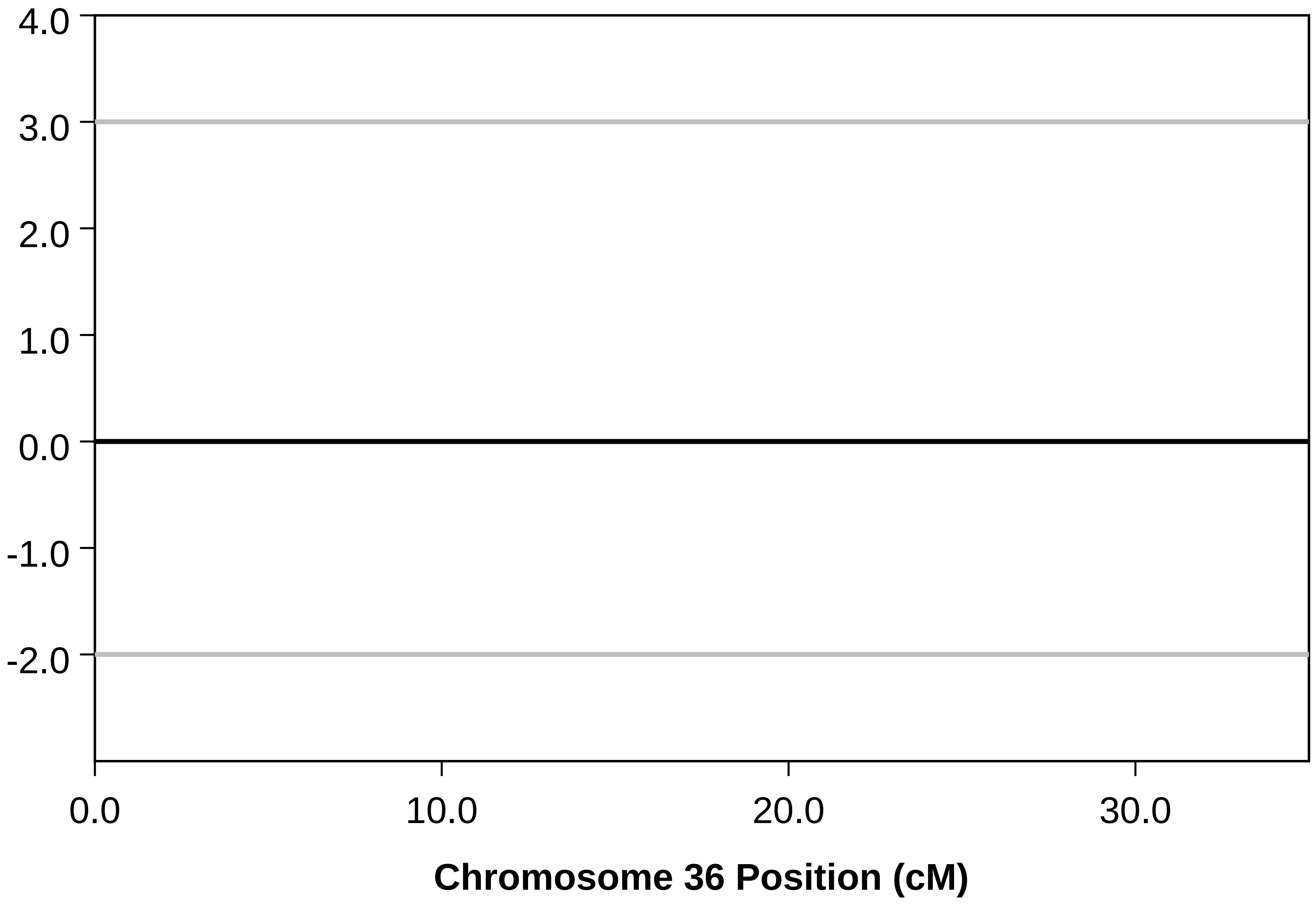

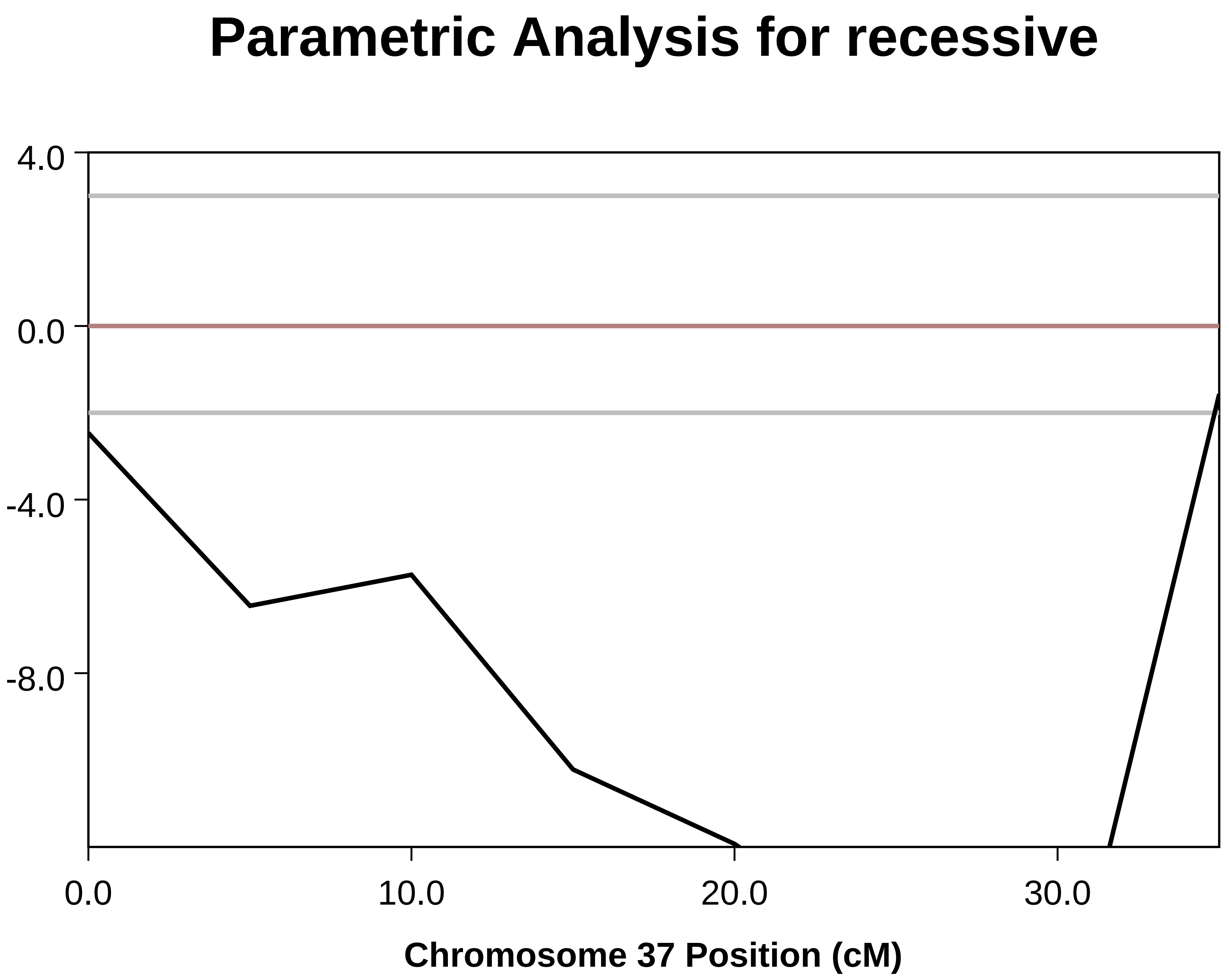
Parametric Analysis for recessive
4.0
0.0
-4.0
-8.0
0.0
10.0
20.0
30.0
Chromosome 37 Position (cM)

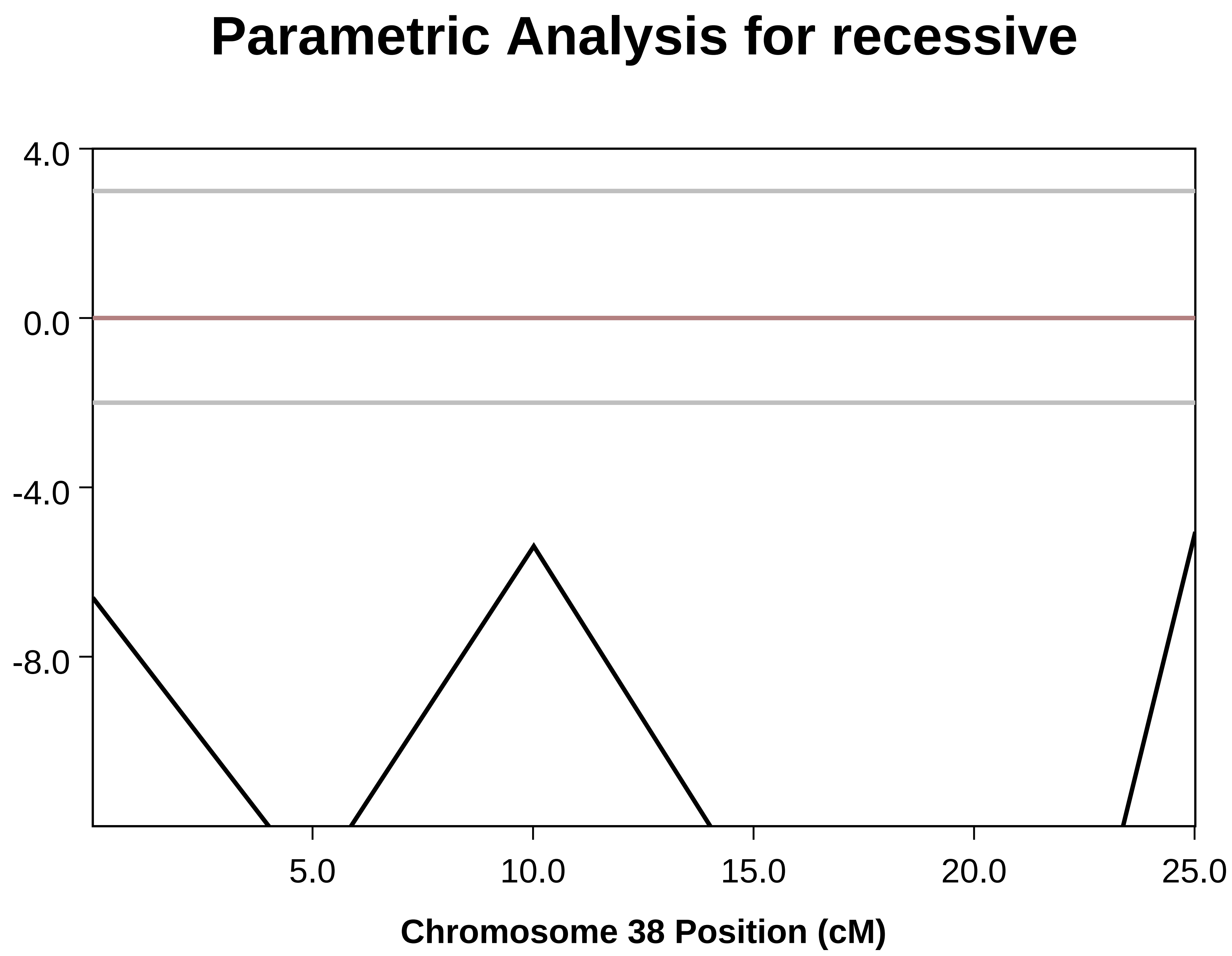
Parametric Analysis for recessive
4.0
0.0
-4.0
-8.0
5.0
10.0
15.0
20.0
25.0
Chromosome 38 Position (cM)

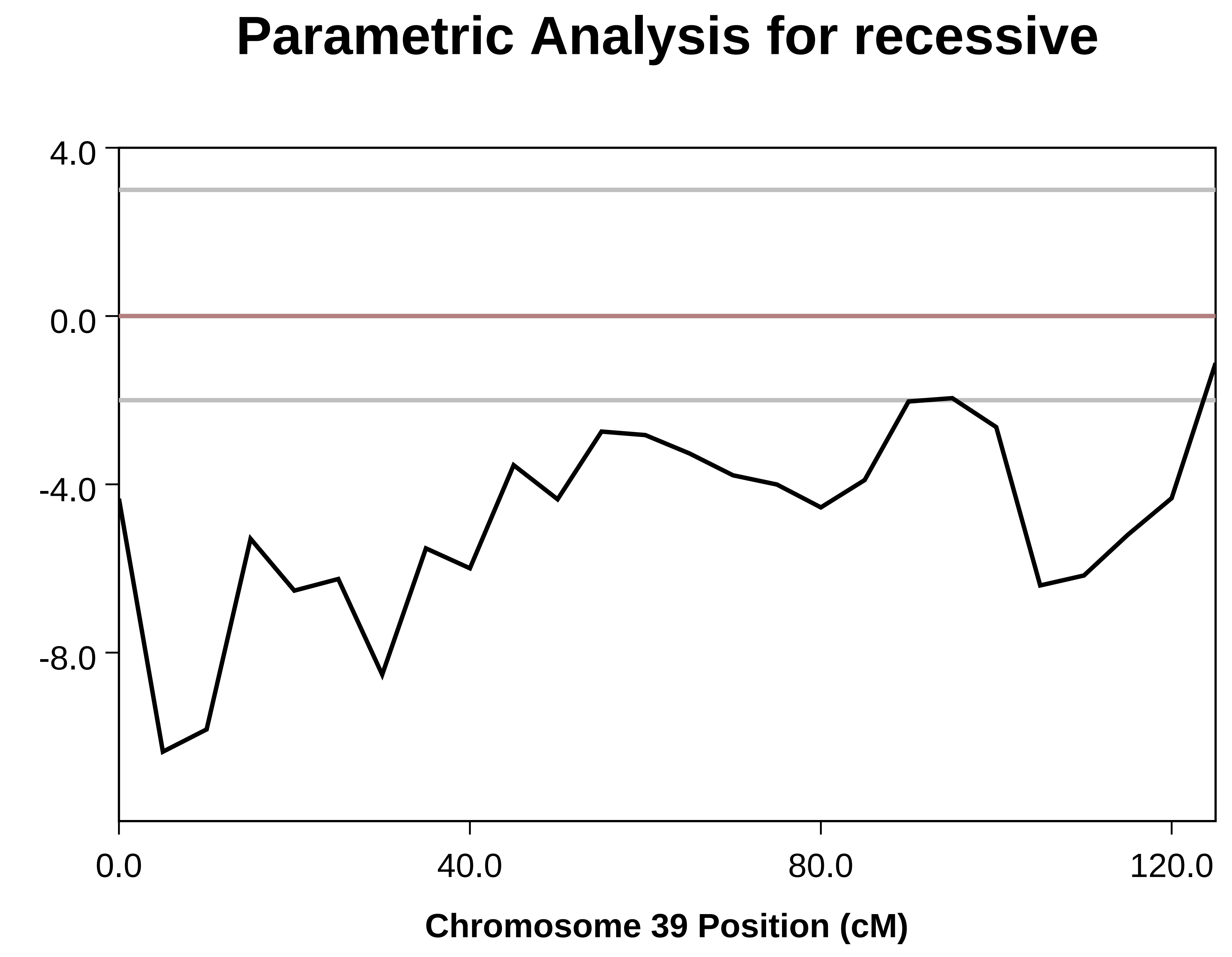

Parametric Analysis for recessive
4.0
0.0
-4.0
-8.0
0.0
40.0
80.0
120.0
Chromosome 39 Position (cM)
