# Supplementary material for: TECPR2 Associated Neuroaxonal Dystrophy in Spanish Water Dogs
Source: PLoS One. 2015 Nov 10;10(11):e0141824. doi: 10.1371/journal.pone.0141824 (PMC4640708; doi:10.1371/journal.pone.0141824)
Supplement: S3 Fig — The arginine residue that is substituted by a tryptophan (p.R1337W) in NAD affected Spanish water dogs is highly conserved in the TECPR1 orthologs. The homology of the sixth and ninth propeller domain of TECPR2 and TECPR1 further indicates the functional relevance of this C-terminal propeller domain. (PDF) [file pone.0141824.s003.pdf]

TECPR2	Dog with mutation	RTVWARC---------PNGDLARWYGVTDKNPAGDYWKKIP
	Dog (*C. lupus*)	.......................R.................
TECPR1	Dog (*C. lupus*)	DQVWVIANKVQGSHGLSRGTVCHRTGVQPREPKGQGWDYGI
	Human (*H. sapiens*)	**************S**************H****H******
	Chimpanzee (*P. troglodytes*)	**************S**************H****H******
	Rhesus macaque (*M. mulatta*)	**************S**************H****H******
	Marmoset (*C. jacchus*)	*******S******S**************H***********
	Gibbon (*N. leucogenys*)	**************S**************H****H******
	Tarsier (*T. syrichta*)	**T****D*********************H***********
	Galago (*O. garnettii*)	**************S**************H***********
	Rat (*R. norvegicus*)	**********************R*M****************
	Mouse (*M. musculus*)	**********************R*M****************
	Hamster (*C.griseus*)	**********************R*M**********S*****
	Chinchilla (*C. lanigera*)	************T*********R******************
	Blind mole rat (*N. galili*)	**************S*******R******************
	Squirrel (*I. tridecemlineatus*)	***
	Egyptian jerboa (*J. jaculus*)	**************S*******R******************
	Guinea pig (*C. porcellus*)	************T*********R******************
	Degu (*O. degus*)	************T*********R******************
	Cattle (*B. taurus*)	*******D******S*******R*****SL***********
	Sheep (*O. aries*)	*******D******S*******R*****SL***********
	Horse (*E. przewalskii*)	**************S**************H***********
	Rabbit (*O. cuniculus*)	*******D******S**************H**R********
	Cat (*F. catus*)	**************S**************************
	Pig (*S. scrofa*)	*******D*********************L****L******
	Ferret (*M. putorius furo*)	****I**D**P**RSM****************M********
	Killer whale (*O. orca*)	**************S*******R******L***********
	Tasmanian devil (*S. harrisii*)	**************S**C***********************
	Canary (*S. canaria*)	*******D******S**C******I****L****LS*****
	Eagle (*H. leucocephalus*)	**L****D******S**C***********M****LS*****
	Pelican (*P. crispus*)	*******D******S**C***********M****LA*****
	Chicken (*G. gallus*)	*******D*********C***********M****LA*****
	Budgerigar (*M. undulatus*)	*******D******S**C***********M****LS*****
	Guppy (*P. reticulata*)	**L*I**D******S**C******L****M*****S*****
	Zebrafish (*D. rerio*)	****I**D**S*HPAE*S******L**K*MQ***LS*****
	Turtle (*C. picta bellii*)	*******D******S**C***********L****LS*****
	Clawed frog (*X. tropicalis*)	**I****D******S**C******L****MQL**HS*****
	Python (*P. bivittatus*)	**I****D******S**C*******R***M****LS*****
	Alligator (*A. mississippiensis*)	*******D******S**C******K****L****LS*****
	Coelacanth (*L. chalumnae*)	****I**D******S**C******V*I**M**I**S*****
	Shark (*C. milii*)	****IT*D******S**C***********L**M*HS*****
	Purple sea urchin (*S. purpuratus*)	*******DQ*D*A----**V**R*D*IASAK*M*KA**K*A
	Saccoglossus kowalevskii	N***IL*A**DA*----*K***R*L**SSDN***TT**L**
	Dog roundworm (*T. canis*)	AS**LLT---------DG*QLFL*I*ICAANIL*EY*YQLD
Peroxin 23	Fruit fly (*D. melanogaster*)	SE**AIS---------LN*II*R*C*ITEEN*A*V**NL**
