# Supplementary material for: TECPR2 Associated Neuroaxonal Dystrophy in Spanish Water Dogs
Source: PLoS One. 2015 Nov 10;10(11):e0141824. doi: 10.1371/journal.pone.0141824 (PMC4640708; doi:10.1371/journal.pone.0141824)
Supplement: S1 Table — (PDF) [file pone.0141824.s005.pdf]

S1 Table: Classification of inherited or idiopathic neuroaxonal dystrophies in humans.

Primary neuroaxonal dystrophies (NAD) represent clinically and genetically heterogeneous neurodegenerative conditions, with prominent spheroid formation in the central and occasionally the peripheral nervous system. Many types of NAD in humans are associated with iron accumulation in the basal ganglia and are classified as neurodegeneration with brain iron accumulation (NBIA) [1]. NAD classification varies among the literature. According to the histopathological similarities to PKAN, MPAN and BPAN should also be considered as NAD [2]. All these conditions were considered as rare diseases. The prevalence of PLAN is unknown. Other types of NAD occur with estimated prevalence of 1-2/1,000,000 for PKAN, 1/1,000,000 for MPAN and less 1/1,000,000 for the remaining types [3]. CNS: central nervous system; ar: autosomal recessive; ad: autosomal dominant; X: X-linked;

**Nomenclature according to genetic classification (and type of NBIA)**	**Underlying mutation and mode of inheritance**	**Subtypes and age of onset**	**Historical or former name**	**Clinical symptoms**	**References/ OMIM entry**
Pantothenate kinase-associated neurodegeneration (PKAN; NBIA1)	*PANK2*<br>Pantothenate Kinase 2<br>ar	classic PKAN: first decade;<br><br>atypical PKAN: later age of onset	"Hallervorden–Spatz disease"	dystonia with predominant oro-lingual-mandibular involvement and spasticity<br><br>atypical PKAN: slower disease progression; psychiatric symptoms may be prominent	[4]-[6]; OMIM 606157
PLA2G6-associated neurodegeneration (PLAN; NBIA2)	*PLA2G6*<br>Phospholipase A2, Group VI<br>ar	infantile NAD: before 2 years; progressive development of iron accumulations<br><br>atypical NAD: late infantile or adult onset; frequently without iron accumulation	"Seitelberger disease": late infantile onset; without iron accumulation	infantile NAD: hypotonia, visual disturbances, motor, and mental retardation.<br><br>atypical NAD: dystonia, dementia, and parkinsonism	[5]-[9]; OMIM 603604
Mitochondrial membrane protein-associated neurodegeneration (MPAN; NBIA5)	*C19orf12*<br>Chromosome 19 Open Reading Frame 12<br>ar	infantile to adulthood (range: 4-30 years)		dysarthria, gait abnormalities, dystonia, and parkinsonism	[6], [10]; OMIM 614297
Beta-propeller associated-neurodegeneration (BPAN, NBIA4)	*WDR45*<br>WD Repeat-Containing Protein 45<br>X	infantile	Static encephalopathy of childhood with neurodegeneration in adulthood (SENDA)	parkinsonism, dystonia, dementia, and global development delay	[6], [11], [12]; OMIM 300526

Late infantile, juvenile and adult NAD	genetically not classified, idiopathic group	late infantile to adulthood		rigidity, ataxia, dysarthria, motor weakness, psychiatric disorders, later dementia	[1]
Hereditary neuroaxonal leukodystophy with spheroids (HDLS)/<br><br>Pigmentary orthochromatic leukodystrophy (POLD)	*CSF1R*<br>Colony Stimulating Factor 1 Receptor<br>ad	late infantile to adulthood (range: 8-78 years)	Subcortical gliosis of Neumann	progressive motor, psychiatric, and cognitive dysfunction leading to dementia; frequent epilepsy	[13]-[16]<br>OMIM 164770
Polycystic lipomembranous osteodysplasia with sclerosing leukoencephalopathy (PLOS;)	*TYROBP*<br>TYRO Protein Tyrosine Kinase Binding Protein<br><br>*TREM2*<br>Triggering Receptor Expressed on Myeloid Cells 2<br>ar	adulthood (20-30 years)	Nasu Hakola disease	repeated bone fractures caused by bone cysts, psychotic symptoms rapidly progressing to presenile dementia	[17], [18]<br>OMIM 604142<br><br>OMIM 605086
Giant axonal neuropathy	*GAN*<br>Gigaxonin<br>ar	varies from soon after birth to up to ten years of age		peripheral neuropathy with progressive weakness and hyporeflexia; in case of CNS lesions: nystagmus, ataxia, spasticity, dysmetria, and dysarthria	[19], [20]<br>OMIM 605379

Supplementary references:

1. Lowe JS, Leigh N. Disorders of movement and system and degeneration. In: Graham DI, Lantos PL, editors. Greenfield's Neuropathology. New York: Oxford University press; 2002. p. 390.
2. Kruer MC. The neuropathology of neurodegeneration with brain iron accumulation. Int Rev Neurobiol. 2013:110: 165-194.
3. Orphanet: an online database of rare diseases and orphan drugs. Copyright, INSERM 1997. Available at http://www.orpha.net Accessed (29 November 2014).
4. Hallervorden J, Spatz H. Eigenartige Erkrankung im extrapyramidalen System mit besonderer Beteiligung des Globus pallidus und der Substantia nigra. Z Gesammte Neurolog Psychiatr. 1922:79: 254-302.

5. Gregory A, Polster BJ, Hayflick SJ. Clinical and genetic delineation of neurodegeneration with brain iron accumulation. J Med Genet. 2009:46: 73-80.
6. Levi S, Finazzi D. Neurodegeneration with brain iron accumulation: update on pathogenic mechanisms. Front Pharmacol. 2014:5: 99.
7. Gilman S, Barrett RE. Hallervorden-Spatz disease and infantile neuroaxonal dystrophy. Clinical characteristics and nosological considerations. J Neurol Sci. 1973:19: 189-205.
8. Schneider SA, Hardy J, Bhatia KP. Syndromes of neurodegeneration with brain iron accumulation (NBIA): an update on clinical presentations, histological and genetic underpinnings, and treatment considerations. Mov Disord. 2012:27: 42-53.
9. Colombelli C, Aoun M, Tiranti V. Defective lipid metabolism in neurodegeneration with brain iron accumulation (NBIA) syndromes: not only a matter of iron. J Inherit Metab Dis. 2015:38: 123-136.
10. Hogarth P, Gregory A, Kruer MC, Sanford L, Wagoner W, Natowicz MR, et al. New NBIA subtype: genetic, clinical, pathologic, and radiographic features of MPAN. Neurology. 2013:80: 268-275.
11. Kimura Y, Sato N, Sugai K, Maruyama S, Ota M, Kamiya K, et al. MRI, MR spectroscopy, and diffusion tensor imaging findings in patient with static encephalopathy of childhood with neurodegeneration in adulthood (SENDA). Brain Dev. 2013:35: 458-461.
12. Saitsu H, Nishimura T, Muramatsu K, Kodera H, Kumada S, Sugai K, et al. De novo mutations in the autophagy gene WDR45 cause static encephalopathy of childhood with neurodegeneration in adulthood. Nat Genet. 2013:45: 445-449.
13. Wider C, Van Gerpen JA, DeArmond S, Shuster EA, Dickson DW, Wszolek ZK. Leukoencephalopathy with spheroids (HDLS) and pigmentary leukodystrophy (POLD). A single entity? Neurology. 2009:72: 1953-1959.
14. Kleinfeld K, Mobley B, Hedera P, Wegner A, Sriram S, Pawate S. Adult-onset leukoencephalopathy with neuroaxonal spheroids and pigmented glia: report of five cases and a new mutation. J Neurol. 2013:260: 558-571.
15. Kinoshita M, Kondo Y, Yoshida K, Fukushima K, Hoshi K, Ishizawa K, et al. Corpus callosum atrophy in patients with hereditary diffuse leukoencephalopathy with neuroaxonal spheroids: an MRI-based study. Intern Med. 2014:53: 21-27.
16. Riku Y, Ando T, Goto Y, Mano K, Iwasaki Y, Sobue G, et al. Early pathologic changes in hereditary diffuse leukoencephalopathy with spheroids. J Neuropathol Exp Neurol. 2014:73: 1183-1190.
17. Paloneva J, Kestilä M, Wu J, Salminen A, Böhling T, Ruotsalainen V, et al. Loss-of-function mutations in TYROBP (DAP12) result in a presenile dementia with bone cysts. Nat Genet. 2000:25: 357-361.
18. Paloneva J, Manninen T, Christman G, Hovanes K, Mandelin J, Adolfsson R, et al. Mutations in two genes encoding different subunits of a receptor signaling complex result in an identical disease phenotype. Am J Hum Genet. 2002:71: 656-662.
19. Yang Y, Allen E, Ding J, Wang W. Giant axonal neuropathy. Cell Mol Life Sci. 2007:64: 601-609.
20. Gordon N. Giant axonal neuropathy. Dev Med Child Neurol. 2004:46: 717-719.
