# Supplementary material for: TECPR2 Associated Neuroaxonal Dystrophy in Spanish Water Dogs
Source: PLoS One. 2015 Nov 10;10(11):e0141824. doi: 10.1371/journal.pone.0141824 (PMC4640708; doi:10.1371/journal.pone.0141824)
Supplement: S2 Table — (PDF) [file pone.0141824.s006.pdf]

S2 Table: GeneBank accession numbers used for the TECPR2 and TECPR1 alignments.

Species	GeneBank accession number TECPR2	GeneBank accession number TECPR1/Peroxin 23
C. lupus	XP_005623885.1	XP_546986.4
H. sapiens	XP_005268303.1	AAH53591.1
P. troglodytes	XP_009426768.1	XP_009451946.1
M. mulatta	AFJ70931.1	AFE77616.1
C. jacchus	JAB46771.1	JAB48654.1
N. leucogenys	XP_003276268.1	XP_004090980.1
T. syrichta	XP_008062282.1	XP_008071091.1
O. garnettii	XP_003799373.1	XP_003798974.1
R. norvegicus	XP_008763192.1	NP_001032268.1
M. musculus	NP_001276439.1	NP_081686.1
C. griseus	XP_007624662.1	EGW02538.1
C. lanigera	XP_005410807.1	XP_005397968.1
N. galili	XP_008823812.1	XP_008822244.1
I. tridecemlineatus	XP_005341060.1	XP_005339780.1
J. jaculus	XP_004665590.1	XP_004666407.1
C. porcellus	XP_003463177.2	XP_003469934.1
O. degus	XP_004645455.1	XP_004629763.1
B. taurus	NP_001179619.1	XP_010824155.1
O. aries	XP_004018206.1	XP_004021346.1
E. caballus/E. przewalskii	XP_001491516.3	XP_008514290.1
O. cuniculus	XP_008246877.1	XP_008249440.1
F. catus	XP_003988050.1	XP_003998502.1
S. scrofa	XP_003482366.1	XP_003124363.2
M. putorius furo	XP_004796722.1	XP_004775015.1
O. orca	XP_004262466.1	XP_004269089.1
S. harrisii	XP_003756583.1	XP_003761538.1
S. canaria	XP_009099981.1	XP_009090096.1
H. leucocephalus	XP_010582268.1	XP_010568535.1
P. crispus	XP_009481196.1	XP_009476684.1
G. gallus	XP_421376.4	E1BZR9.1
M. undulatus	XP_005149617.1	XP_005145341.1
P. reticulata	XP_008396922.1	XP_008436384.1
D. rerio	XP_005158848.1	XP_002661342.3
C. picta bellii	XP_008166397.1	XP_008162871.1
X. tropicalis	XP_002936632.2	NP_001107288.1
P. bivittatus	XP_007435199.1	XP_007425221.1
A. mississippiensis	XP_006270459.1	XP_006265339.1
L. chalumnae	XP_006010486.1	XP_006000845.1
C. milii	XP_007886502.1	XP_007891753.1
S. purpuratus		XP_796102.3
S. kowalevskii		XP_006811775.1
T. canis		KHN83943.1
D. melanogaster		AAF49038.2
